# Genetics, Morphology, Advertisement Calls, and Historical Records Distinguish Six New Polyploid Species of African Clawed Frog (*Xenopus*, Pipidae) from West and Central Africa

**DOI:** 10.1371/journal.pone.0142823

**Published:** 2015-12-16

**Authors:** Ben J. Evans, Timothy F. Carter, Eli Greenbaum, Václav Gvoždík, Darcy B. Kelley, Patrick J. McLaughlin, Olivier S. G. Pauwels, Daniel M. Portik, Edward L. Stanley, Richard C. Tinsley, Martha L. Tobias, David C. Blackburn

**Affiliations:** 1 Department of Biology, Life Sciences Building Room 328 McMaster University, Hamilton, Ontario, Canada; 2 Biomedical Sciences, Ontario Veterinary College, University of Guelph, Guelph, Ontario, Canada; 3 Department of Biological Sciences, University of Texas at El Paso, El Paso, Texas, United States of America; 4 Institute of Vertebrate Biology, Czech Academy of Sciences, Kvetna 8, Brno, Czech Republic; 5 Department of Zoology, National Museum, Prague, Czech Republic; 6 Department of Biological Sciences, Columbia University, New York, New York, United States of America; 7 Department of Biology, Papadakis Integrated Sciences Building, Drexel University, Philadelphia, Pennsylvania, United States of America; 8 Département des Vertébrés Récents, Instítut Royal des Sciences Naturelles de Belgique, Brussels, Belgium; 9 Museum of Vertebrate Zoology, University of California, Berkeley, California, United States of America; 10 California Academy of Sciences, San Francisco, California, United States of America; 11 School of Biological Sciences, University of Bristol, Bristol, United Kingdom; Radboud University Nijmegen, NETHERLANDS

## Abstract

African clawed frogs, genus *Xenopus*, are extraordinary among vertebrates in the diversity of their polyploid species and the high number of independent polyploidization events that occurred during their diversification. Here we update current understanding of the evolutionary history of this group and describe six new species from west and central sub-Saharan Africa, including four tetraploids and two dodecaploids. We provide information on molecular variation, morphology, karyotypes, vocalizations, and estimated geographic ranges, which support the distinctiveness of these new species. We resurrect *Xenopus calcaratus* from synonymy of *Xenopus tropicalis* and refer populations from Bioko Island and coastal Cameroon (near Mt. Cameroon) to this species. To facilitate comparisons to the new species, we also provide comments on the type specimens, morphology, and distributions of *X*. *epitropicalis*, *X*. *tropicalis*, and *X*. *fraseri*. This includes significantly restricted application of the names *X*. *fraseri* and *X*. *epitropicalis*, the first of which we argue is known definitively only from type specimens and possibly one other specimen. Inferring the evolutionary histories of these new species allows refinement of species groups within *Xenopus* and leads to our recognition of two subgenera (*Xenopus* and *Silurana*) and three species groups within the subgenus *Xenopus* (*amieti*, *laevis*, and *muelleri* species groups).

## Introduction

African clawed frogs (genus *Xenopus*, Pipidae, subfamily Xenopodinae) are found across sub-Saharan Africa. These frogs prefer slow moving or stagnant water as tadpoles and as adults, although migration between bodies of water occurs [1]. A consequence of their aquatic way of life is that a suite of morphological features distinguishes them from other anurans. These features include a streamlined and flattened body, a vocal organ specialized for underwater sound production, lateral-line organs, claws on the first three (inner) toes, and fully webbed toes. Species distributions can be expansive, as in the case of *X*. *laevis* which occurs over much of southern Africa [2] with introduced populations on other continents. Other species have miniscule distributions, such as that of *X*. *longipes*, which occurs exclusively in one lake. These frogs have been used as food [3], for human pregnancy tests [4], and as a model organisms for a range of biological investigations [5, 6]. The species *Xenopus tropicalis* has recently become extensively used for biological research because of its relatively short time to maturation, smaller size, and diploid genome [6]. The first amphibian genome to be sequenced was that of *X*. *tropicalis* [7] and genome sequencing of *X*. *laevis* is underway [8].

### Evolutionary relationships, allopolyploidization, and hybridization

Monophyly of African clawed frogs is well supported, for example [9], and this clade within the family Pipidae is referred to as the subfamily Xenopodinae [5]. African clawed frogs are distinguished from most other amphibian lineages by a remarkably high incidence of polyploid species, reviewed in [10]. For nearly three decades, these species have been placed in two genera, *Xenopus* and *Silurana* [5], corresponding to clades differing in morphology [11, 12] and in the number of chromosomes of their diploid ancestors (20 for *Silurana* and 18 for *Xenopus*). However, a previously proposed paraphyletic relationship between *Silurana* and *Xenopus* with respect to other pipid genera based on morphology [11] has not been supported by recent molecular phylogenetic studies that recover monophyly of Xenopodinae with respect to other pipid genera [9, 13, 14]. Additionally, the community relying on these as animal models for laboratory studies usually refers to all of these species as *Xenopus*, for example [7] and a previous summary of *Xenopus* systematics placed these into two distinct subgenera, *Silurana* and *Xenopus* [15]. We therefore continue the tradition established by Kobel *et al*. [15] by recognizing *Silurana* as a subgenus of the genus *Xenopus*. The subgenus *Silurana* comprises two described species, the diploid *X*. *tropicalis* and the tetraploid *X*. *epitropicalis*, and two additional tetraploid species [10], which we describe and resurrect here. Based on a recent taxonomic revision of *X*. *laevis* [2], the subgenus *Xenopus* comprises 20 described species, including eleven tetraploids (*X*. *borealis*, *X*. *clivii*, *X*. *fraseri*, *X*. *gilli*, *X*. *laevis*, *X*. *largeni*, *X*. *muelleri*, *X*. *petersii*, *X*. *poweri*, *X*. *pygmaeus*, and *X*. *victorianus*), seven octoploids (*X*. *amieti*, *X*. *andrei*, *X*. *boumbaensis*, *X*. *itombwensis*, *X*. *lenduensis*, *X*. *wittei*, and *X*. *vestitus*), and two dodecaploids (*X*. *longipes* and *X*. *ruwenzoriensis*). Here, we describe four additional tetraploids and two additional dodecaploids, and resurrect another tetraploid species from synonymy with *X*. *tropicalis*; all of these are from Central and West Africa.

For the most part, analysis of the molecular evolutionary history of African clawed frogs has relied on a portion of the mitochondrial DNA genome spanning most of the mitochondrial *12S* and *16S* rDNA genes and all the intervening *tRNA* ^*val*^, a portion of the mitochondrial cytochrome oxidase I gene, and cloned homeologs of the autosomal genes *RAG1* and *DMRT1* [16–23]. Using this approach, a recent study that included genetic data from four of the new species described here proposed that tetraploidization occurred at least once in subgenus *Silurana* and once in subgenus *Xenopus*, octoploidization occurred at least three times in subgenus *Xenopus*, and that dodecaploidization occurred at least three times in subgenus *Xenopus* [21]. Phylogenetic analyses indicate that all of these genome duplication events were definitively by allopolyploidization rather than autopolyploidization, except tetraploidization in subgenus *Xenopus*. The ancestral (2*n* = 18) diploid(s) is/are not available for comparison, and possibly extinct, although allopolyploidization seems to be the most likely mechanism of tetraploidization based on its role in other genome duplications in this group [10]. Alternative scenarios for allotetraploidization involving more than one allopolyploidization event in *Xenopus* are discussed in Supplementary Information of Bewick et al. [18]. Not all examples of hybridization in African clawed frogs are associated with genome duplication, and naturally occurring hybrids that have the same ploidy level as the parental species have been reported between *X*. *laevis* and *X*. *muelleri*, between *X*. *laevis* and *X*. *gilli*, and between *X*. *victorianus* and *X*. *borealis* [24–28]. Additional species pairs have been observed in the same body of water, but no hybrids were detected, including *X*. *clivii* and *X*. *largeni* [19, 29], *X*. *victorianus* and *X*. *wittei* [30], and *X*. *vestitus* and *X*. *wittei* [30].

### Objectives

The principal objective of this study is to describe several new species of African clawed frog and to provide information about the evolutionary history of these species. Because many species of *Xenopus* are highly similar in external morphology, we take a multi-faceted approach, first by using molecular phylogenetics to identify distinct lineages and then using vocal characteristics, karyotypes, and both external and internal morphology (including measurements and skeletal anatomy) to provide diagnoses for lineages that we recognize as species. We explicitly compare our data to those for other described species of *Xenopus* that are closely related, morphologically similar, or distributed in the same region. In several cases, we also provide more detail on the distribution and morphology of previously proposed species, and in one case resurrect a taxon long synonymized with *Xenopus tropicalis*. By examining type material of *Xenopus fraseri*, we substantially revise the previous concepts for this species and suggest that this is among the most poorly known of all living *Xenopus* species. We provide updates on evolutionary relationships and genome duplication events as well as refinements of previously proposed species groups. For the species groups for which we describe new species, we also provide short summaries of their evolution, diversity, and distinguishing characteristics.

## Materials and Methods

### Specimens

Our analyses are based on extensive collections of the genus *Xenopus* that are currently available in museum collections. Many of these specimens derive from our field research (BJE, EG, VG, DBK, PJM, OSGP, DMP, RCT, MLT, and DCB), including those with associated vocalization data and tissue samples used for genetic analyses. We made a special effort to include type specimens in our morphological studies and to compare them with the new taxa described below. Institutional codes for collections follow Sabaj Pérez [31]. When available, coordinates for localities utilize the WGS84 datum; others are estimates from Google Earth. Specimens examined, including those for which DNA sequence data were collected (see below), but not morphological measurements, are listed in S1 Table. Some genetic samples were collected from living individuals or from an individual for which a voucher specimen was not preserved or was lost; these samples are archived in the laboratory of BJE and are available upon request.

### Molecular analyses

To provide a framework for identifying distinct lineages and determining their geographic distributions, we collected sequence data from mitochondrial and autosomal DNA for all species of African clawed frogs except *X*. *fraseri*, including the ones described below as new species. From the mitochondrial DNA genome, 506–2,432 base pairs (bp) were sequenced from portions of the *12S* and *16S* rDNA genes and the intervening *tRNA* ^*val*^ gene using primers from Evans et al. [23]. Additionally, for at least one individual per species except *X*. *fraseri*, ~586 bp of the cytochrome oxidase I gene were sequenced using primers from Ivanova *et al*. [32]. Sequence data from a total of 228 individuals were included in the mitochondrial DNA analyses, including at least one representative from every species in the genus (except *X*. *fraseri*), including those previously undescribed. From the nuclear genome, 785–4,208 bp from cloned or directly sequenced homeologs of the recombination activating genes 1 and 2 (*RAG1* and *RAG2* respectively) were sequenced. Sequence data from a total of 78 homeologs from 26 individuals were included in the autosomal DNA analyses, again including at least one representative from almost every species in the genus, including those previously undescribed. For the autosomal DNA analysis, sequence data were not collected from *X*. *poweri*, *X*. *petersii*, or *X*. *victorianus*, each of which is closely related to *X*. *laevis*, or from *X*. *fraseri*, a species for which we lack a genetic sample (see species account below). Additional information on specimens for which molecular data were obtained is provided in S1 Table. All new sequence data collected for this study are deposited in GenBank (accession numbers KT728008 –KT728192). Accession numbers of other data in these analyses are listed in previous papers [16, 20–23]. Specimens for which DNA sequence was obtained from mitochondrial and/or the autosomal genes are listed in S2 Table.

Separate phylogenetic analyses were performed for the mitochondrial data and for the concatenated autosomal data using BEAST version 1.8.2 [33]. For both of these analyses we used a normally distributed prior of 36 million years with a standard deviation of 6 million years for the age of Xenopodinae following the “DNA-Morph-Fossil; tips + 3 node calibration” analysis of Cannatella [34]. For both analyses, the general time reversible model of evolution with a proportion of sites assumed invariant and gamma-distributed rate heterogeneity (GTR+I+ Γ) was selected by the Akaike Information Criterion using jModelTest2 [35, 36]. Both analyses assumed a relaxed log-normal molecular clock and a coalescent constant-population size model of tree shape. Sequence data from *Pipa pipa* was used as an outgroup, although neither analysis enforced monophyly of Xenopodinae. In order to assess whether the Bayesian analysis had converged on the posterior distributions of parameter values, we inspected trends, distributions and the effective sample size (ESS) of parameters from each analysis using Tracer version 1.5 [37]. Based on inspection of the parameter values and likelihoods of the BEAST runs, a burn-in of 1,000,000 generations was applied to each of four independent runs for the mtDNA analysis and a burn-in of 300,000 generations was applied to each of two independent runs for the concatenated *RAG1* + *RAG2* analysis. The ESS of all parameters for was over 200 for both analyses. The posterior distribution of each of these sets of trees was then summarized using Tree Annotator version 1.8.2 [33] as a maximum clade credibility tree using median values for node ages. For comparative purposes, each of these analyses was also performed using a normally distributed prior of 65 million years with a standard deviation of 7 million years for the time of diversification of extant Xenopodinae, following “*BEAST Analysis 3” in Bewick *et al*. [9]; these latter analyses are included as Supplemental material (S1 and S2 Figs).

Because the evolutionary history of this group is characterized by multiple instances of allopolyploidization, reviewed in [10], we cloned and sequenced duplicated homeologs of the recombination activating gene 1 (*RAG1*) and also either directly sequenced portions of the recombination activating gene 2 (*RAG2*), or cloned and sequenced co-amplified homeologs of this gene. *RAG2* is present in a single copy in tetraploids of subgenus *Xenopus* due to a gene loss of one homeolog [16]. The non-deleted gene family of *RAG2* is linked to the β copy of *RAG1* in the “S” subgenome of *X*. *laevis* based on the top BLAST [38] hit to version 9.1 of the draft *X*. *laevis* genome assembly on xenbase.org [8]; these sequences were therefore concatenated for phylogenetic analysis. Because the α copy of *RAG2* was lost from the other “L” subgenome prior to diversification of tetraploids of subgenus *Xenopus* [16], these homeologous data were treated as missing (that is, they were coded as gaps in the portion of the alignment with the *Xenopus* β homeolog of *RAG2* and the *Silurana* α and β homeologs of *RAG2*).

### Cytogenetics

Karyotypes were performed either using methods described in Evans *et al*. [21] or Pokorná *et al*. [39].

### Morphology

Descriptions of the new species are based on examination of preserved specimens and comparisons to most of the relevant type material. BJE collected measurement data for male and female specimens of both new and previously described species; measurements for two syntypes of *X*. *calcaratus* were taken by VG and F. Tillack at Museum für Naturkunde, Berlin. For type specimens, these measurements include a subset of those detailed by Tinsley [40], including snout–vent length (SVL), head width at level of subocular tentacle, snout length, eye diameter, interocular distance (the distance between the inner bases of the circum-orbital plaques), lower forelimb length, and crus length. We additionally measured the length of the foot (ankle to longest toe). When possible, sex of individuals was inferred on the basis of presence of nuptial pads on the forearms and absence of a protruding cloaca for males, or presence of a protruding cloaca for females. These data are provided in S1 Table.

One of us (DMP) counted the number of lateral-line plaques present in five distinct rows on adult specimens, represented in parentheses as sum of lateral-line rows of Shelton [41]: orbital (supra-orbital + post-orbital + infra-orbital); oral (maxillary + mandibular + tentacular); medial (upper lateral + occipital); lateral (parietal + middle lateral); and ventral (posterior lower + anterior lower). In some cases the individual specimens measured by BJE differ from those used by DMP to count lateral-line plaques, though in most cases the individuals counted are a subset of those measured. These data are provided in S1 Table.

High-resolution x-ray Computed Tomography (CT) scans of ethanol-preserved specimens were produced using a Nano-focus phoenix v|tome|x s240 x-ray CT scanner at the GE Inspection Technologies, LP Technical Solutions Center in San Carlos, CA. CT-scanning provides a non-destructive way of visualizing internal morphology of specimens in three dimensions. These scans were produced from 1000 x-ray images taken of the specimen as it rotated 360º. For each scan, the current and voltage of the x-ray source and the capture-time of the detector were modified to optimize the resolution and gray-scale range (the range of discernibly different densities) of the images. At each angle, three x-ray images were taken and then averaged to reduce noise. When necessary, specimens were scanned in several sections and recompiled afterwards to maximize the resolution (S3 Table). The raw x-rays were then processed using GE’s proprietary datos|x software, which converted them into a series of tomogram images. These “slices” were then compiled, viewed and analysed using VG Studio Max 2.2.1 (Volume Graphics, Heidelberg, Germany). For each scan, the whole skeleton, skull, and key axial bones were reconstructed separately to facilitate comparisons. Additional information on these scans is provided in S3 Table.

The heads of two *Xenopus calcaratus* syntypes, ZMB 8255 and ZMB 74681 (formerly also ZMB 8255), were scanned by Kristin Mahlow at the Museum für Naturkunde, Berlin using a Phoenix nanotom X-ray|s tube at 80kV and 200μA, generating 1000 projections with 750ms per scan. The effective voxel size is thus 12µm. Cone beam reconstruction was performed using the datos|x 2.2.1 reconstruction software (GE Sensing & Inspection Technologies GMBH phoenix|x-ray).

### Vocalization

Previous studies indicated that the male advertisement call is acoustically distinct for each species, but male and female release call features overlap across species [42, 43]. We thus recorded and analyzed male advertisement calls as described in Evans *et al*. [21] and Tobias *et al*. [42]. Data were obtained or are previously available from all species except *X*. *longipes* and *X*. *fraseri*. Briefly, male advertisement calls were evoked by injection of human chorionic gonadotropin (50–200 international units depending on body size; Sigma, Oakville, Ontario, Canada). Vocalizations were recorded ~6 hours after injection after placing a sexually unreceptive female in the same aquarium as the male. Recordings of most of the new species were obtained in small ~10 liter plastic aquaria that were two-thirds full of water using a High Tech hydrophone connected to a laptop computer via a PreSonus AudioBox 22VSL Audio/midi interface (Baton Rouge, LA, USA). Most of the vocalizations from other species to which the new vocalizations were compared were recorded in a laboratory setting as detailed in Tobias *et al*. [42]. Recordings were analyzed as detailed in Tobias *et al*. [42].

Vocalizations of African clawed frogs are composed of a pulse or a series of sound pulses. Following Tobias *et al*. [42], we collected and compared information for each species including the number of pulses within a call, the rate that pulses were produced within a call (the inter-pulse interval or IPI), the two dominant frequencies of pulses (including the lower one, DF1, and the higher one, DF2), and the degree of intensity modulation (IM), defined as the fold change in intensity of the minimum intensity pulse to the maximum intensity pulse divided by the intensity of the minimum intensity pulse. The sound pulses that make up male advertisement calls in *Xenopus* species have two dominant frequencies; the comparative magnitude of the amplitude of the amplitude of each dominant frequency varies [42]. Following Tobias *et al*. [42], we therefore refer to the lower dominant frequency as dominant frequency 1 and the higher dominant frequency as dominant frequency 2. We categorized these calls into four categories (click-type, burst-type, trill-type, biphasic) based on the criteria described in Tobias et al. [42]. For example, click type calls consist of only one pulse, burst-type and trill-type calls have more than one click but burst-type calls have fewer (2–14) than trill-type (43–127). Biphasic calls have two rather than one temporal pattern.

### Nomenclatural Acts

The electronic edition of this article conforms to the requirements of the amended International Code of Zoological Nomenclature, and hence the new names contained herein are available under that Code from the electronic edition of this article. This published work and the nomenclatural acts it contains have been registered in ZooBank, the online registration system for the ICZN. The ZooBank LSIDs (Life Science Identifiers) can be resolved and the associated information viewed through any standard web browser by appending the LSID to the prefix "http://zoobank.org/". The LSID for this publication is: urn:lsid:zoobank.org:pub: F9F51F48-7477-4AEB-90B8-4388142D1577. The electronic edition of this work was published in a journal with an ISSN, and has been archived and is available from the following digital repositories: PubMed Central, LOCKSS.

## Results

Because of the paucity of anatomical information on diverse species of *Xenopus*, we provide summaries for the genus, each subgenus, and two species groups. In addition, we provide accounts for specific species, including six new species that we describe below.

### Taxonomic Accounts

#### Genus *Xenopus* Wagler, 1827 [44]

All species in the genus *Xenopus* have size dimorphism (females larger than males), fully webbed feet, a dorsoventrally compressed body, relatively smooth skin, and lateral-line organs. The tadpoles are suspension feeders that are morphologically similar across species and notable for their slit-like anteriorly directed mouth, a pair of spiracles, conspicuous barbels, and lack of keratinized mouthparts. The two subgenera (*Silurana* and *Xenopus*) are distinguished by a number of morphological, genetic, karyotype, and host-parasite characters (see below).

Species of *Xenopus* have compressed bodies that are oblong and ovoid in dorsal view. The head is subtriangular, and the rostrum projects just beyond the lower jaw, though species vary in the degree to which the rostrum is blunt or pointed. The canthus rostralis is typically flat to weakly concave, the loreal region is generally flat, and the internarial region varies from flat to weakly concave. All species lack a tongue and have a single opening to the Eustachian tubes. The floor of the mouth is typically wrinkled and covered in small pustules. The posteroventrally directed choanae are large, rounded, and largely or entirely visible in ventral view. Premaxillary and maxillary teeth are present, but vomerine teeth are nearly always absent. The nares are prominent ellipsoid slits directed dorsally; a small sheet of skin projects from the margins of each naris, the extent and morphology of which varies among species but often features a laterally projecting nubbin. The size of the eye relative to the head varies among species as does the extent to which the eye is covered by the lower eyelid. In preservative, the pupil is typically round. Each eye is encircled by lateral-line plaques located on a raised ring of skin, though the degree to which this ring is observable varies based on specimen preservation. A subocular tentacle extends from the lateral margin of each eye; the length of this tentacle varies among species and is absent in two species (*X*. *gilli* and *X*. *largeni*). All species lack an externally visible tympanic annulus. The skin is generally smooth, although it can be covered by small spicules (especially those in the subgenus *Silurana*). A prominent feature of all adult *Xenopus* is the lateral-line system; the individual plaques (each comprising multiple sensory organs) resemble stitches. Distinct lateral-line rows extend across the skin of the head and dorsal, lateral, and ventral body. In both males and females, the medial surfaces of the manual digits are covered by small black punctiform spicules. In males, these are accentuated into nuptial pads comprising sheets of darkly pigmented spicules on the manual digits as well as on the upper arm, forearm, and sometimes axillary region. The forelimbs are typically moderately robust and have elongate manual digits that lack webbing (in contrast to other African pipids, the dwarf clawed frogs *Hymenochirus* and *Pseudhymenochirus*). The relative length of the manual digits varies among species, though in all species these digits typically terminate in small bulb-like tips. Being primarily aquatic frogs, these species have large hind limbs with fully webbed feet (i.e., extending to either the toe tip or base of the keratinous claw). The extent to which the pedal webbing is pigmented varies among species. As in most other frogs, the digits of the foot are longer than those of the hand, with the fourth toe being the longest and the first the shortest. As their common name of African clawed frogs suggests, all species have dark brown or black keratinous claws on the first three pedal digits; similar to the hand, those pedal digits that lack keratinous claws terminate in bulb-like tips. Many species have a keratinous claw on the prehallux, which in combination with other characters, can be diagnostic of particular species groups. The hands and feet lack subarticular tubercles, though scattered pustules are found on the plantar surface of some species. Female *Xenopus* are often identifiable by their protruding cloacal lobes, the number and/or fusion of which varies among species. In life, the coloration of most species ranges from grays to browns, sometimes with patterning or markings that are indicative of particular species.

Morphological differentiation among adult *Xenopus* varies from substantial to subtle, with few species having unique distinguishing characteristics. With a few exceptions, body size (SVL) and lateral-lines are insufficient for differentiating most of these species (Tables 1–3). *Xenopus gilli* is distinguished by its unique dorsal pattern consisting of longitudinal dark brown blotches separated by pale brown coloration. *Xenopus longipes* is distinguished by feet that are large relative to its small body size. The four species previously recognized as *Xenopus laevis*, for example [15], which are now designated *X*. *laevis*, *X*. *petersii*, *X*. *poweri*, and *X*. *victorianus* [2], are distinguished both by the large body size of adults, especially the population of *X*. *laevis* from the Cape Region in South Africa [45], and by the large size of the eyes relative to body size. *Xenopus muelleri* and *X*. *fraseri* are the only described extant species in the genus with vomerine teeth [46–48], though see comments below regarding the new tetraploid species of the *muelleri* species group.

**Table 1:** Measurements in millimeters of selected type specimens, including types of new species described here.

Species	Museum	Sex	Type	SVL	IO	EO	MO	FL	HW	SFE	KA	EF	ER	OLL	MLL	LLL	VLL
*subgenus Silurana*																	
*X*. *calcaratus*	ZMB 8255	F	Lectotype	53.3	7.0	9.9	11.4	33.5	9.7	3.0	25.3	17.3	-	-	-	18	-
*X*. *calcaratus*	ZMB 74681	F	Paralectotype	49.9	6.5	9.4	11.3	31.6	9.3	2.9	23.2	16.2	-	-	-	18	-
*X*. *epitropicalis*	BMNH 1982.462	F	Holotype	67.6	6.5	10.4	12.3	32.4	10.7	3.6	24.6	18.6	12	11	21	23	19
*X*. *mellotropicalis*	NCSM 76797	M	Holotype	48.4	5.6	9.0	10.1	26.2	9.3	2.5	22.0	18.0	11	12	17	17	13
*X*. *mellotropicalis*	NCSM 78871	F	Paratype	46.3	5.4	8.2	9.2	27.1	8.3	2.4	21.4	15.2	11	13	17	19	18
*X*. *mellotropicalis*	NCSM 78881	-	Paratype	31.3	4.3	6.6	7.2	19.8	6.3	2.2	15.0	11.5	-	-	-	-	-
*X*. *mellotropicalis*	MHNG 2644.058	M	Paratype	45.7	5.0	9.3	8.2	27.0	7.2	2.5	15.2	16.7	-	-	-	-	-
*subgenus Xenopus*																	
*amieti group*																	
*X*. *allofraseri*	CAS 207765	F	Holotype	48.0	6.2	10.5	13.0	27.3	10.3	3.2	18.4	17.2	-	-	-	-	-
*X*. *allofraseri*	MCZ A-148161	F	Paratype	33.5	4.6	7.7	9.7	22.1	7.8	2.1	15.8	13.4	14	12	16	20	-
*X*. *allofraseri*	MCZ A-148162	M	Paratype	36.6	5.0	8.2	10.2	23.0	8.5	2.9	16.6	14.0	13	15	15	20	-
*X*. *allofraseri*	MCZ A-148163	F	Paratype	42.5	5.4	9.2	42.2	24.3	8.9	2.6	17.5	15.0	12	14	16	21	-
*X*. *allofraseri*	MCZ A-148164	F	Paratype	38.8	5.4	8.9	11.8	23.6	8.8	2.8	18.0	14.8	15	14	17	23	17
*X*. *allofraseri*	MCZ A-148166	M?	Paratype	28.3	4.2	7.2	8.9	18.6	7.3	2.1	13.5	12.3	11	11	15	18	-
*X*. *allofraseri*	MCZ A-148167	M	Paratype	34.8	4.6	8.1	10.8	22.7	8.4	2.6	16.7	15.3	12	12	16	21	-
*X*. *allofraseri*	MCZ A-148168	M	Paratype	30.3	4.6	7.7	9.0	20.4	7.4	2.2	14.7	13.1	11	10	15	20	-
*X*. *allofraseri*	MCZ A-148169	F	Paratype	31.7	4.5	7.6	9.1	22.2	7.5	1.9	16.2	13.9	13	13	17	22	-
*X*. *allofraseri*	MCZ A-148170	F	Paratype	27.4	4.1	7.0	8.6	17.8	6.8	2.0	12.9	11.4	13	13	16	18	-
*X*. *allofraseri*	MCZ A-148172	M	Paratype	37.8	4.6	8.3	10.3	23.3	8.2	2.2	16.8	15.4	13	13	16	19	-
*X*. *allofraseri*	MCZ A-148173	F	Paratype	34.5	4.5	7.8	9.6	21.2	7.6	2.0	15.7	13.9	12	12	15	19	-
*X*. *allofraseri*	MCZ A-148174	M	Paratype	34.7	4.9	8.4	10.7	22.9	8.1	2.0	16.1	14.8	13	13	15	18	-
*X*. *allofraseri*	MCZ A-148175	F	Paratype	32.7	4.4	7.6	9.4	21.6	7.9	2.2	15.2	14.0	13	12	14	20	-
*X*. *allofraseri*	MCZ A-148176	M	Paratype	35.5	4.9	8.0	10.0	22.6	7.8	2.0	16.6	14.7	12	12	16	20	17
*X*. *allofraseri*	MCZ A-148177	F	Paratype	41.7	5.9	9.9	11.7	24.1	8.8	2.4	17.9	15.7	14	11	15	20	-
*X*. *allofraseri*	MCZ A-148178	F	Paratype	43.2	5.8	9.9	12.2	24.4	9.5	2.6	18.4	15.8	12	12	15	23	-
*X*. *amieti*	MHNG 2030.80	F	Holotype	49.0	5.8	10.1	11.8	24.2	9.0	2.9	18.9	14.1	13	15	19	24	23
*X*. *amieti*	MHNG 2030.82	M	Paratype	35.0	4.9	8.3	9.9	21.0	7.6	2.3	15.2	14.2	17	13	25	24	23
*X*. *amieti*	MHNG 2030.81	M?	Paratype	34.9	4.7	8.3	10.6	21.8	8.7	2.9	14.9	13.9	15	11	25	26	27
*X*. *andrei*	MHNG 2088.32	F	Holotype	38.4	4.4	8.2	9.6	19.5	6.9	2.2	15.6	14.2	10	13	16	16	17
*X*. *boumbaensis*	MHNG 2088.31	F	Holotype	52.5	6.0	10.0	14.6	28.5	9.6	3.3	22.3	18.0	13	12	14	18	17
*X*. *eysoole*	MCZ A-138016	F	Holotype	39.1	5.2	-	10.3	-	8.4	2.5	16.4	15.0	14	11	20	23	17
*X*. *eysoole*	MCZ A-138017	F	Paratype	37.4	5.4	8.6	10.6	23.7	8.7	2.7	16.9	13.9	13	11	20	20	18
*X*. *eysoole*	MCZ A-148094	M	Paratype	41.7	5.6	-	11.6	-	8.2	2.7	17.2	16.4	13	10	21	18	-
*X*. *eysoole*	MCZ A-148095	F	Paratype	51.7	6.5	-	13.6	-	10.5	3.5	20.3	17.3	10	10	20	19	13?
*X*. *eysoole*	MCZ A-148127	F	Paratype	51.5	6.0	-	13.2	-	9.6	3.5	20.4	12.4	12	10	20	23	13?
*X*. *eysoole*	MCZ A-148128	F	Paratype	47.5	6.1	-	12.5	-	8.7	3.3	19.4	16.0	12	10	19	22	-
*X*. *eysoole*	MCZ A-148129	M	Paratype	39.1	5.2	-	11.2	-	8.1	2.9	16.9	11.1	13	10	19	21	-
*X*. *eysoole*	MCZ A-148130	M	Paratype	41.0	5.1	-	11.4	-	9.0	2.9	17.7	15.3	13	10	17	20	-
*X*. *eysoole*	MCZ A-148131	F	Paratype	49.6	5.5	-	13.3	-	9.0	3.1	19.0	16.4	13	11	18	18	14
*X*. *fraseri*	BMNH 1947.2.24.78	M?	Lectotype	37.2	5.1	8.7	11.5	28.4	8.1	2.8	18.4	15.5	13	13	21	21	24
*X*. *fraseri*	BMNH 1947.2.24.79	M?	Paralectotype	32.5	4.4	7.6	9.0	22.7	7.6	2.8	20.5	12.3	-	15	21	25	-
*X*. *itombwensis*	MCZ A-138192	M	Holotype	31.5	4.0	7.0	9.4	22.0	7.3	2.5	14.1	13.2	-	-	-	-	-
*X*. *kobeli*	MCZ A-148037	F	Holotype	42.1	5.8	9.2	12.0	25.4	8.7	3.1	18.6	15.6	11	11	14	16	-
*X*. *kobeli*	MCZ A-148036	F	Paratype	42.7	5.3	8.5	11.7	25.6	8.6	2.6	17.7	14.9	12	10	16	17	13
*X*. *kobeli*	MCZ A-148059	F	Paratype	43.5	5.5	9.1	11.7	24.4	8.7	2.5	17.6	14.8	12	11	17	22	14
*X*. *kobeli*	MCZ A-148035	M	Paratype	34.9	4.6	7.6	8.9	20.2	7.7	2.3	15.1	12.5	11	10	16	16	-
*X*. *kobeli*	MCZ A-148065	M	Paratype	33.4	4.5	7.6	8.9	19.9	7.0	1.8	14.8	13.6	13	-	16	20	-
*X*. *kobeli*	MCZ A-148066	M	Paratype	33.7	4.7	8.3	9.5	21.6	8.1	2.3	14.9	14.2	11	12	19	21	-
*X*. *kobeli*	MCZ A-148038	F	Paratype	44.9	4.9	8.2	11.3	23.9	8.9	3.2	18.5	15.0	13	10	16	19	-
*X*. *kobeli*	MCZ A-148039	F	Paratype	40.4	5.5	8.9	10.6	25.3	8.7	2.5	17.9	15.7	11	10	15	18	13
*X*. *kobeli*	MCZ A-148060	M	Paratype	33.4	4.5	7.8	9.0	21.1	7.5	2.4	14.5	14.1	11	-	15	20	-
*X*. *kobeli*	MCZ A-148061	M	Paratype	35.4	4.6	7.6	8.8	19.8	7.4	2.1	15.1	13.4	12	10	16	20	-
*X*. *kobeli*	MCZ A-148062	M	Paratype	37.9	4.7	8.1	9.7	23.0	7.8	2.6	16.8	14.7	14	-	18	21	-
*X*. *kobeli*	MCZ A-148063	F	Paratype	46.6	5.6	9.3	12.4	25.1	10.7	2.9	19.0	16.3	13	12	16	21	21
*X*. *lenduensis*	MCZ A-139853	M	Holotype	37.7	5.2	-	-	-	8.4	2.9	16.6	14.6	-	-	-	-	-
*X*. *longipes*	MHNG 2497.10	F	Holotype	34.0	4.2	7.9	8.9	22.3	6.8	2.1	12.3	12.1	12	-	13	16	-
*X*. *longipes*	MHNG 2496.76	F	Paratype	30.9	4.0	7.0	7.8	19.3	6.2	1.9	11.6	11.1	11	9	13	19	17
*X*. *longipes*	MHNG 2496.77	F	Paratype	28.7	4.9	7.3	8.6	18.7	6.2	2.1	10.9	10.2	-	-	-	-	-
*X*. *longipes*	MHNG 2496.94	F	Paratype	30.5	3.7	7.5	8.6	19.8	5.9	2.0	11.4	10.0	10	10	13	21	14?
*X*. *longipes*	MHNG 2496.95	F	Paratype	31.7	3.9	7.5	8.2	18.9	6.3	2.3	11.6	10.9	11	8	13	21	-
*X*. *longipes*	MHNG 2496.96	F	Paratype	31.0	4.0	7.9	9.0	20.4	7.3	2.3	11.8	11.6	11	8	8	17	-
*X*. *parafraseri*	MCZ A-148034	F	Holotype	40.7	4.7	8.1	9.9	22.9	8.2	2.4	16.2	13.8	11	10	19	17	14
*X*. *parafraseri*	CAS 253368	-	Paratype	31.2	4.0	7.2	8.5	20.8	6.7	2.2	14.6	12.6	-	-	-	-	-
*X*. *parafraseri*	CAS 253332	F	Paratype	32.0	3.8	6.7	8.6	22.7	6.9	1.8	14.7	13.2	-	-	-	-	-
*X*. *parafraseri*	CAS 253333	F	Paratype	38.1	4.5	7.6	10.0	23.8	7.5	2.3	16.8	13.4	-	-	-	-	-
*X*. *parafraseri*	CAS 253589	F	Paratype	41.0	5.0	8.5	9.2	24.9	7.6	2.5	17.2	13.1	-	-	-	-	-
*X*. *parafraseri*	CAS 253590	F	Paratype	41.8	5.3	9.0	10.7	24.7	7.9	2.4	17.3	13.4	-	-	-	-	-
*X*. *parafraseri*	CAS 253609	F	Paratype	38.4	4.4	7.7	10.0	24.9	7.4	2.4	17.5	13.7	-	-	-	-	-
*X*. *parafraseri*	CAS 253768	F	Paratype	36.2	4.8	7.8	10.2	21.6	7.9	2.5	16.3	13.2	-	-	-	-	-
*X*. *parafraseri*	CAS 253591	M	Paratype	33.1	3.8	7.0	9.0	21.6	6.8	2.2	14.4	13.4	-	-	-	-	-
*X*. *parafraseri*	CAS 253769	M	Paratype	38.4	4.9	8.1	9.6	24.3	8.1	2.6	16.4	14.4	-	-	-	-	-
*X*. *ruwenzoriensis*	MHNG 2238.15	F	Lectotype	37.1	4.4	7.6	9.3	22.0	6.8	2.5	16.0	12.3	11	10	18	19	18
*X*. *ruwenzoriensis*	MHNG 2238.18	F	Paralectotype	39.5	4.5	7.3	9.7	20.7	7.7	2.7	15.4	12.2	11	11	14	17	-
*X*. *ruwenzoriensis*	MHNG 2238.20	F	Paralectotype	36.1	4.3	8.0	9.1	19.7	6.7	2.6	14.6	12.9	11	12	17	19	18
*X*. *ruwenzoriensis*	MHNG 2238.19	M?	Paralectotype	37.6	4.2	8.1	8.4	19.5	7.1	2.7	16.1	13.3	14	12	17	19	22
*laevis group*																	
X. poweri*	MHNG 1017.74	M?	Holotype	50.1	6.0	10.6	13.1	28.7	10.3	2.9	21.8	19.5	15	13	21	27	19
X. poweri*	MHNG 1017.82	F	Paratype	46.9	5.8	10.0	12.9	29.1	9.0	3.0	19.8	18.3	13	11	20	22	24
X. poweri*	MHNG 1017.81	M	Paratype	50.8	5.6	10.9	14.0	31.0	10.3	2.9	21.4	20.5	15	12	21	24	20
X. poweri*	MHNG 1017.79	M?	Paratype	54.2	6.2	11.1	13.6	30.1	10.6	3.6	21.6	19.8	13	16	20	18	21
X. victorianus**	MCZ A-14616	-	Holotype	35.2	4.5	9.3	9.2	21.7	7.6	2.3	14.0	13.1	-	-	-	-	-
*muelleri group*																	
*X*. *fischbergi*	CAS 255060	F	Holotype	51.7	6.2	11.6	13.5	27.3	3.1	2.5	18.4	18.2	-	-	-	-	-
*X*. *fischbergi*	CAS 255059	F	Paratype	61.0	7.4	13.8	16.9	34.5	10.6	3.4	26.0	21.7	-	-	-	-	-
*X*. *fischbergi*	CAS 255061	F	Paratype	62.6	7.8	13.3	14.9	38.4	11.1	2.9	24.6	24.3	-	-	-	-	-

Abbreviations refer to the Museum number (Museum), type status of specimen (Type), snout vent length (SVL), intraocular distance (IO), extraocular distance (EO), mouth width (MO), foot length (FL), head width at the level of the subocular tentacle (HW), snout to front eye length (SFE), knee to ankle length (KA), elbow to longest finger length (EF), and counts of eye ridges (ER), oral lateral lines (OLL), medial lateral lines (MLL), lateral lateral lines (LLL), and ventral lateral lines (VLL). For Origin, the Democratic Republic of the Congo is abbreviated DRC. Dashes indicate missing data; question marks indicate uncertainty. Data from *X*. *tropicalis* types are not listed because they are not adults. Data are lacking for types not listed.

* Holotype or paratypes of subspecies *X*. *laevis sudanensis*, a probable synonym of *X*. *poweri* (Furman et al. 2015)

** Holotype of subspecies *X*. *laevis bunyoniensis*, a probable synonym of *X*. *victorianus* (Furman et al. 2015)

**Table 2:** Summary of snout vent length (SVL) in milimeters for African clawed frogs.

species	aveF	maxF	stdevF	#F	aveM	maxM	stdevM	#M
subgenus *Silurana*								
*X*. *calcaratus*	44	59	12	19	-	-	-	-
*X*. *epitropicalis*	56	68	5	12	43	49	2	12
*X*. *mellotropicalis*	53	58	6	14	45	51	3	12
*X*. *tropicalis*	51	59	10	18	42	44	2	16
subgenus *Xenopus*								
*amieti* group								
*X*. *allofraseri*	34	48	7	17	35	38	3	6
*X*. *amieti*	45	49	2	6	37	40	2	10
*X*. *andrei*	48	53	4	11	35	37	2	12
*X*. *boumbaensis*	36	53	9	31	34	37	3	13
*X*. cf. *boumbaensis*	45	48	4	11	35	37	1	14
*X*. *eysoole*	48	52	6	9	40	42	2	4
X. fraseri*	-	-	-	-	35	37	3	2
*X*. *itombwensis*	36	38	3	2	31	37	2	12
*X*. *kobeli*	42	47	3	7	35	38	2	7
*X*. *lenduensis*	48	56	5	27	40	46	3	15
*X*. *longipes*	30	34	2	8	26	29	1	8
*X*. *parafraseri*	37	42	3	14	34	38	4	3
*X*. *pygmaeus*	33	36	3	2	28	28	1	2
X. ruwenzoriensis*	38	48	2	4	37	39	2	6
*X*. *wittei*	49	54	3	17	41	45	3	15
*X*. *vestitus*	49	54	6	38	40	43	2	17
*laevis* group								
*X*. *gilli*	51	61	6	65	39	42	2	22
*X*. *laevis*	86	119	15	43	63	83	11	66
*X*. *poweri*	59	71	6	16	46	56	11	22
*X*. *victorianus*	60	68	10	10	50	57	3	14
*muelleri* group								
*X*. *borealis*	57	75	16	20	40	48	7	8
*X*. *clivii*	62	78	13	24	50	59	7	25
*X*. *fischbergi*	56	63	8	6	51	52	2	2
*X*. *muelleri*	73	81	6	19	57	61	3	13
*X*. *largeni*	45	54	5	9	41	44	1	13

Data include average SVL for females and males (aveF, aveM, respectively), the maximum SVLs (maxF, maxM, respectively), standard deviations (stdevF and stdevM, respectively), and the number of individuals measured (#F, #M). Data from *X. vestitus* are from Tinsley (1973). Dashes indicate missing data for listed species; data are entirely missing for *X. petersii*.

* sex of maximum-sized individual was ambiguous but listed in male column.

**Table 3:** Summary of lateral line counts

Species	ER	OLL	MLL	LLL	VLL
subgenus *Silurana*					
*X*. *calcaratus*	10.4 (9–13); 17	10.3 (9–12); 17	16.4 (15–19); 17	18.4 (15–21); 17	17.4 (14–21); 17
*X*. *epitropicalis*	12.0 (12–12); 1	11.0 (11–11); 1	21.0 (21–21); 1	23.0 (23–23); 1	19.0 (19–19); 1
*X*. *mellotropicalis*	11.0 (12–12); 4	11.3 (10–13); 4	17.25 (16–19); 4	19.0 (16–19); 4	14.3 (10–18); 4
*X*. *tropicalis*	10.3 (8–12); 15	10.7 (9–12); 15	17.9 (16–21); 15	19.9 (17–22); 15	18.3 (16–21); 15
subgenus *Xenopus*					
*ameiti* group					
*X*. *allofraseri*	12.7 (11–15); 18	12.6 (10–15); 18	15.5 (13–17); 18	20.3 (18–23); 18	17.0 (17–17); 2
*X*. *amieti*	13.2 (11–17); 12	10.5 (8–15); 20	18.6 (14–25); 25	20.9 (18–26); 25	19.1 (13–27); 11
*X*. *andrei*	10.0 (10–10); 1	13.0 (13–13); 1	16.0 (16–16); 1	16.0 (16–16); 1	17.0 (17–17); 1
*X*. *boumbaensis*	12.7 (10–16); 31	12.8 (11–15); 31	17.2 (14–21); 31	21.5 (18–25); 31	20.6 (17–25); 31
*X*. *eysoole*	12.5 (10–14); 13	10.6 (9–13); 13	19.8 (17–23); 13	20.4 (18–23); 13	15.5 (13–18); 4
*X*. *fraseri*	13.0 (13–13); 1	14.0 (13–15); 2	21.0 (21–21); 2	23.0 (21–25); 2	24.0 (24–24); 1
*X*. *kobeli*	10.7 (10–12); 9	16.2 (14–19); 12	19.3 (16–22); 12	15.3 (13–21); 4	15.3 (13–21); 4
*X*. *longipes*	11.4 (10–13); 11	8.8 (8–10); 4	13.3 (8–16); 11	17.1 (14–21); 11	17.0 (17–17); 1
*X*. *parafraseri*	10.7 (9–13); 33	10.6 (8–13); 32	15.9 (11–20); 35	18.2 (15–21); 35	16.1 (14–19); 19
*X*. *ruwenzoriensis*	11.8 (11–14); 4	11.3 (10–12); 4	16.5 (14–18); 4	18.5 (17–19); 4	19.3 (18–22); 3
*laevis* group					
*X*. *poweri*	13.7 (13–15); 9	13.2 (11–16); 9	21.7 (19–28); 9	24.4 (18–29); 9	21.8 (19–25); 6
muelleri *group*					
*X*. *borealis*	13.5 (10–16); 45	15.0 (11–18); 44	25.2 (21–31); 45	26.8 (19–31); 45	24.8 (18–29); 31
*X*. *muelleri*	13.6 (11–16); 17	14.4 (11–18); 17	28.1 (24–32); 17	28.6 (24–34); 17	26.0 (17–32); 17
*X*. *fischbergi*	13.5 (12–15); 4	14.8 (11–16); 4	25.3 (24–26); 4	28.0 (26–31); 4	22.7 (22–24); 3

Eye ridges (ER), oral lateral lines (OLL), medial lateral lines (MLL), lateral lateral lines (LLL), and ventral lateral lines (VLL) of representative species. Numbers indicate the average, range in parentheses, and the number of individuals counted. Data are lacking for species not listed.

#### Subgenus *Silurana* Gray, 1864 [49]

**Fig 1:**
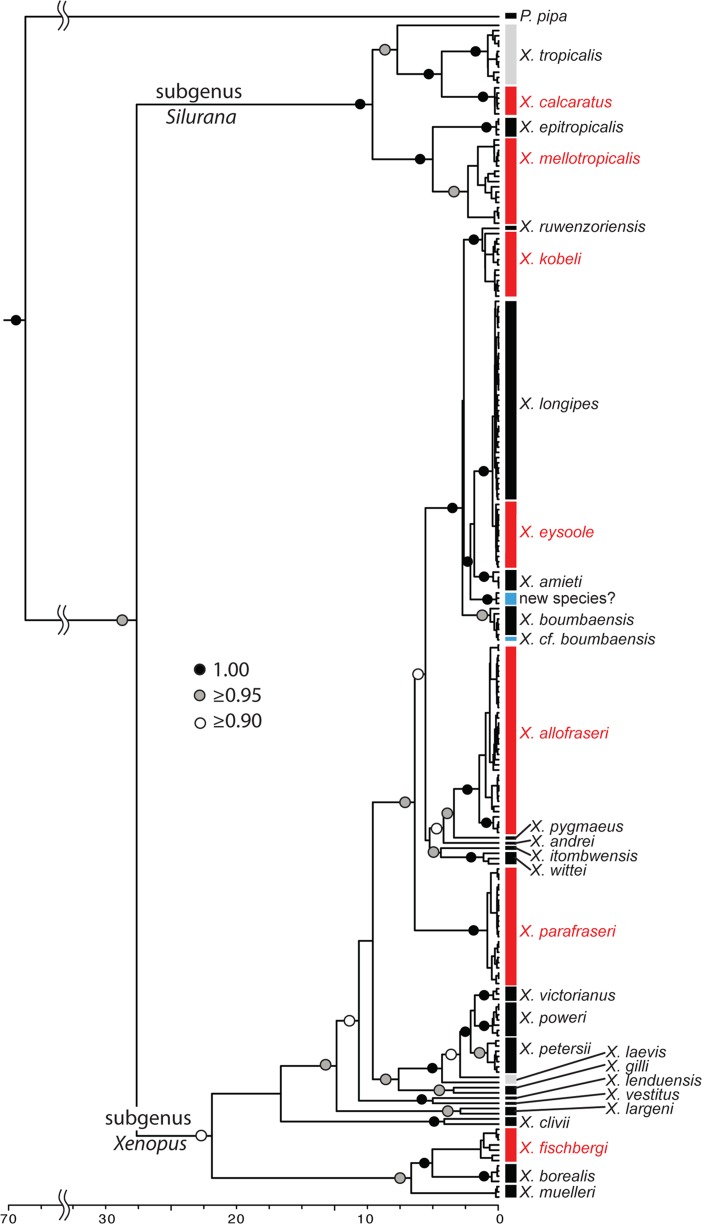
Mitochondrial DNA chronogram using calibration from [34]. New and resurrected species detailed here are indicated in red, possible additional new species are indicated with blue, and species with paraphyletic mtDNA are indicated with gray. Dots subtending nodes indicate posterior probabilities as indicated by the key with some values over terminal clades omitted for clarity. The scale is in units of millions of years.

**Fig 2:**
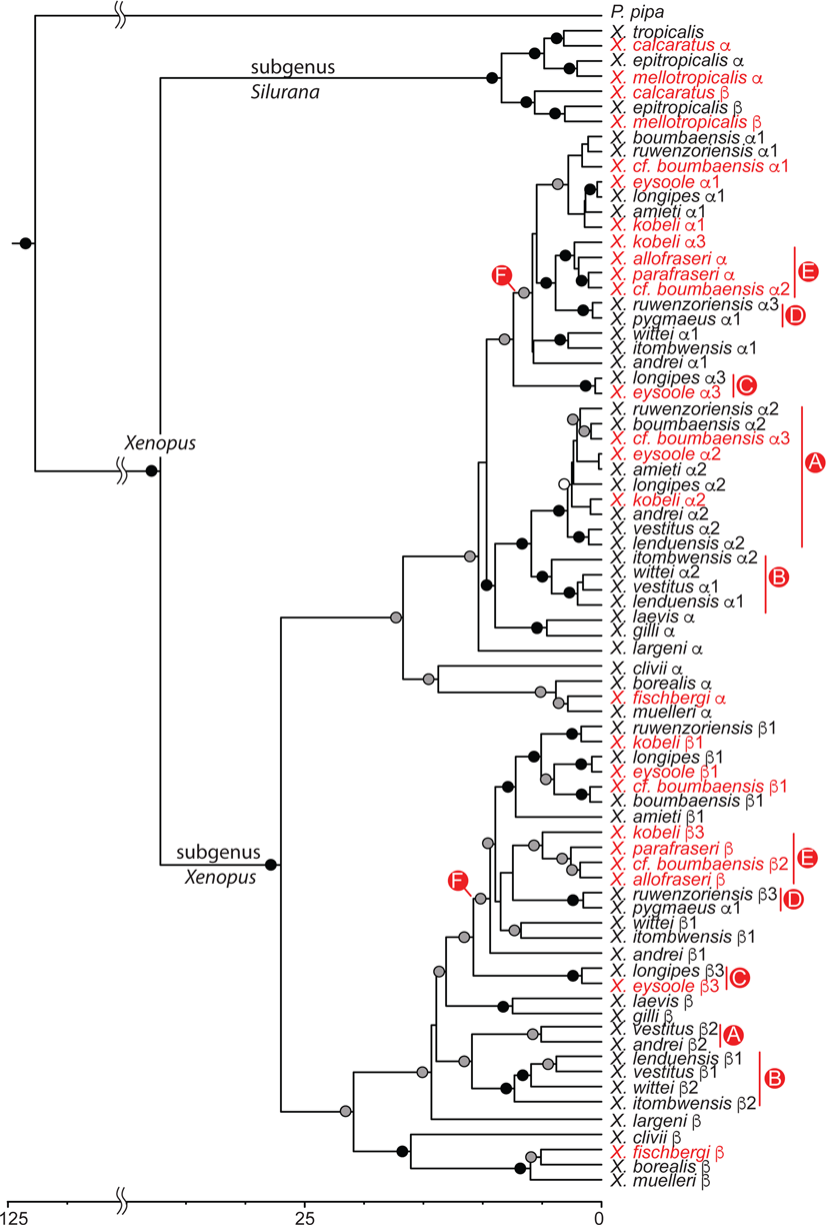
RAG1/ RAG2 chronogram using calibration from [34]. Labeling follows Fig 1 with the addition that tetraploid homeologs are indicated with α and β and, for octoploid and dodecaploids, each of these homeologs classes is further divided into two or three categories indicated by numbers. Homeologs of new and resurrected species are in red. Letters in circles indicate homeologous lineages inferred to be descended from six tetraploid ancestral species A–F. Data are lacking from *X*. *petersii*, *X*. *poweri*, and *X*. *victorianus*, which form a clade with *X*. *laevis*, and from *X*. *fraseri*.

**Fig 3:**
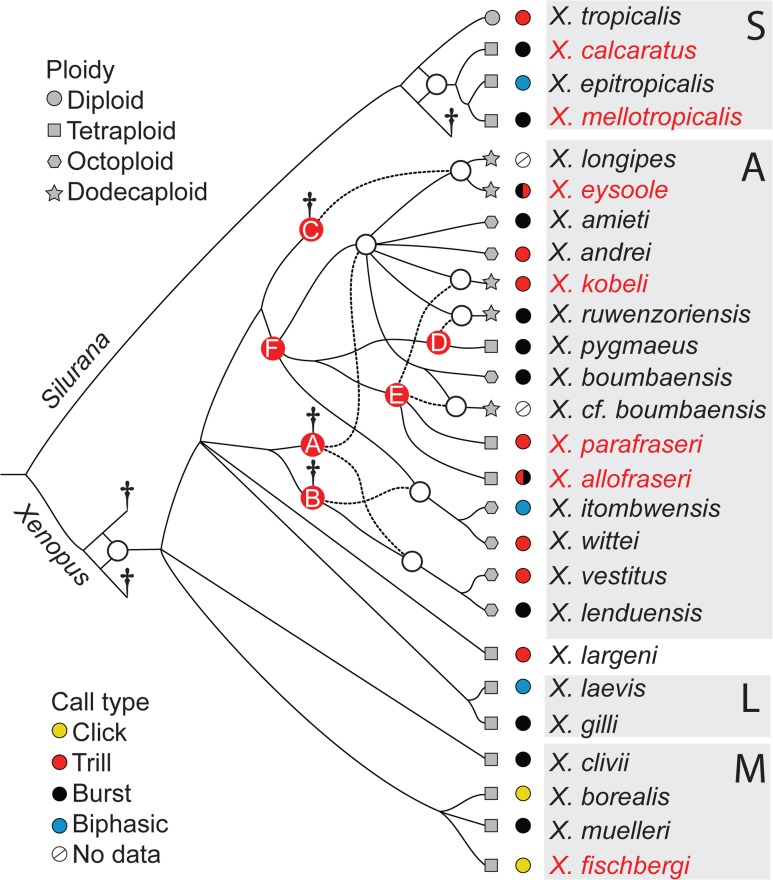
Summary phylogeny. A summary phylogeny inferred by comparing the mitochondrial and autosomal gene trees depicted in Figs 1 and 2. New and resurrected species described here are in red. S, A, L, and M refer to subgenus *Silurana*, and the *amieti*, *laevis*, and *muelleri* species groups within subgenus *Xenopus* respectively. Dotted lines indicate paternal ancestral lineages. Circles over internal nodes indicate allopolyploidization events; shapes on branch tips indicate ploidy of extant species; colored next to these shapes circles indicate call type inferred from this study and Tobias et al. [42]. Letters over red dots refer to ancestors whose homeologous lineages are labled in Fig 2. Daggers indicate lost ancestors, including up to three diploid species (assuming allotetraploidization in subgenus *Xenopus*) and at least three tetraploid ancestors (A, B, and C).

**Fig 4:**
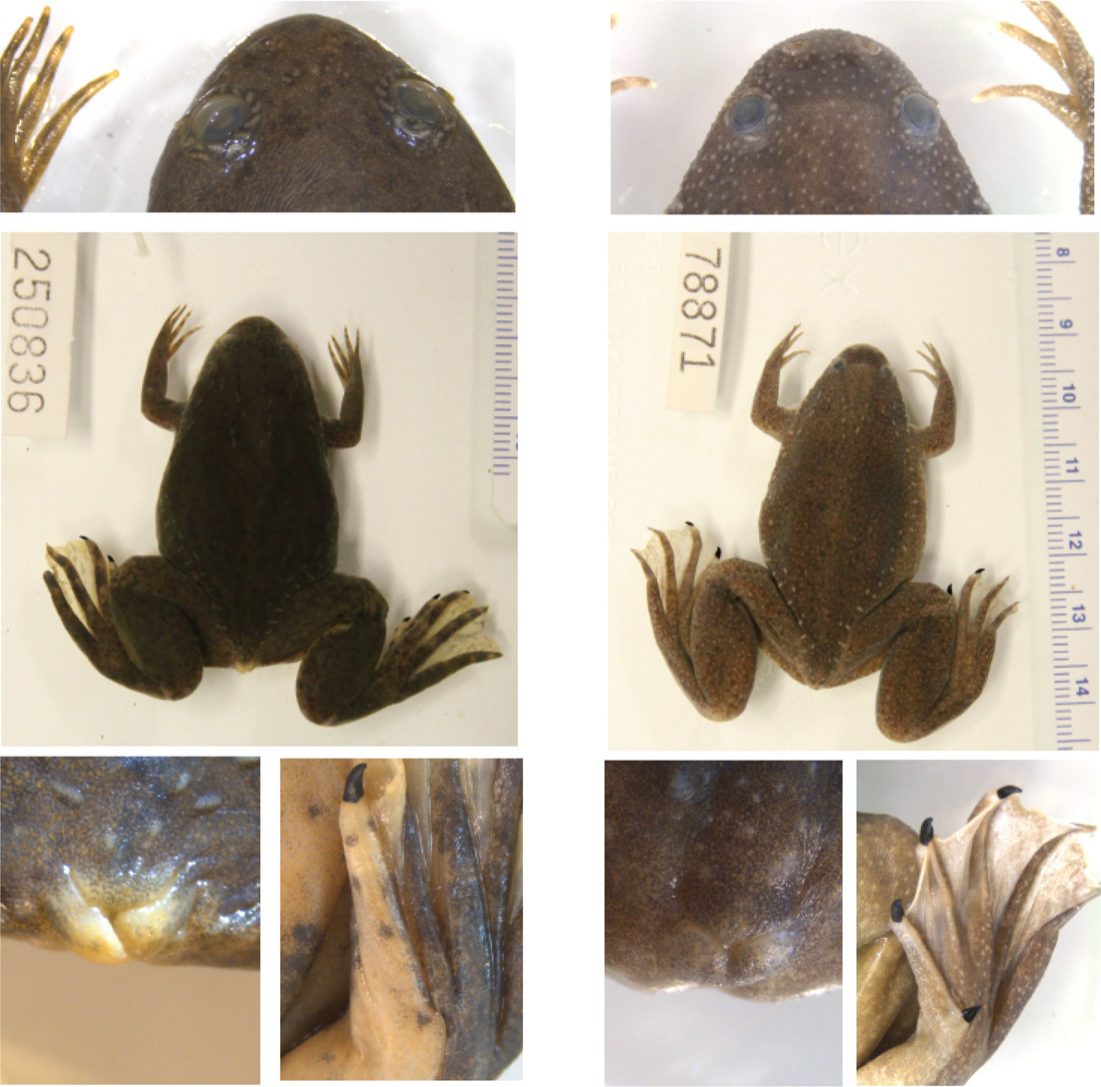
External morphology of subgenera *Silurana* and *Xenopus*. Comparison of external morphology of subgenera *Xenopus* (right, specimen of *X*. *victorianus* CAS 250836 [DCB-202]) and *Silurana* (left, *X*. *calcaratus* CAS 207759) including in *Silurana* rougher skin, relatively smaller eyes, relatively shorter subocular tentacle (in comparison to sympatric *Xenopus* species), and relatively less of eye covered by lower eyelid (top), relatively shorter feet (middle), and ventrally fused cloacal lobes, claw on prehallux, and lack of skin ridge on first pedal digit from the prehallus (bottom).

**Fig 5:**
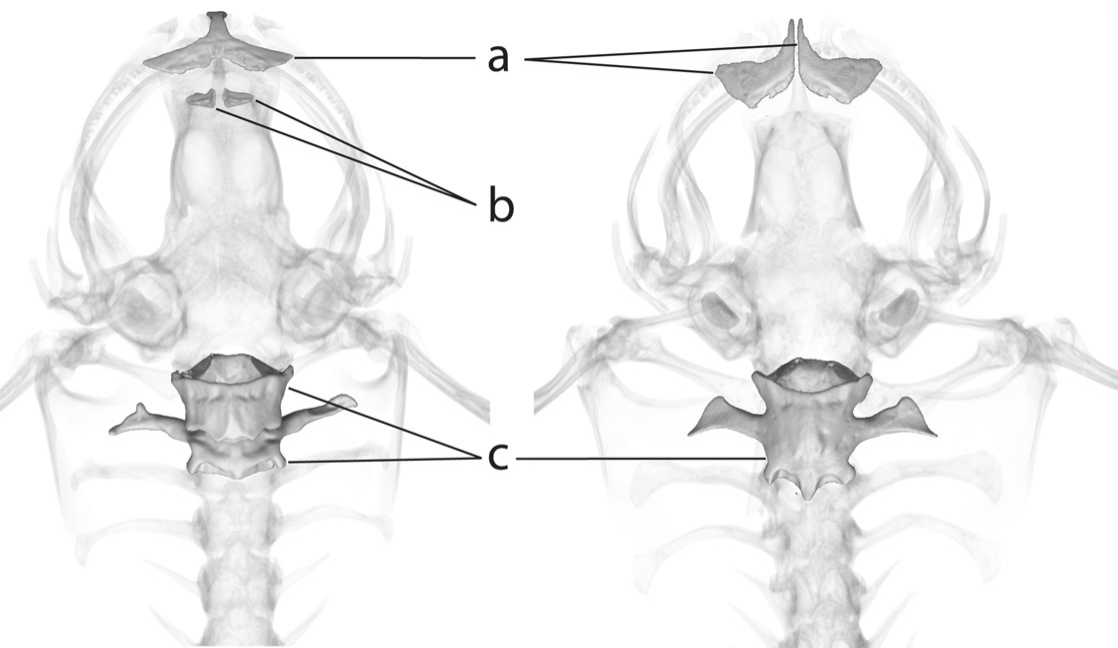
Internal morphology of subgenera *Silurana* and *Xenopus*. Comparison of osteology of subgenera *Xenopus* (left, holotype specimen of *Xenopus amieti*, MHNG 2030.80 and *Silurana* (right, *X*. *calcaratus*, CAS 207759). Differences include (a) paired nasal bones in subgenus *Silurana* but not subgenus *Xenopus*, (b) absence of the vomer bones in the palate of subgenus *Silurana* but not subgenus *Xenopus* and (c) fusion of the first two presacral vertebrae in subgenus *Silurana* but not subgenus *Xenopus*.

Genetic data from the mitochondrial and nuclear genomes strongly support a clade of species that is sister to all other living species of *Xenopus* (Figs 1–3) [9, 16, 20–23]. We use *Silurana* as a subgenus following Kobel et al. [15]. *Silurana* contains four species from West and Central Africa: *X*. *tropicalis*, *X*. *epitropicalis*, and two other species described below (one new, one resurrected). In general, these medium-sized species in the subgenus *Silurana* are distinguished from species in the subgenus *Xenopus* by the following combination of external morphological features (Fig 4): (1) cloacal lobes fused ventrally; (2) keratinous claws on prehallux as well as the first three toes; (3) many small spicules across the dorsum; (4) lack of a dermal ridge extending along the first toe from the prehallux; (5) many scattered tubercles on the plantar surface; (6) relatively short feet; (7) relatively small eyes; (8) relatively little of the eye covered by the lower eyelid; (9) relatively shorter subocular tentacle in comparison to the sympatric species in the subgenus *Xenopus*; (10) generally fewer plaques in each row of the lateral-line system than in the subgenus *Xenopus*, though the ranges can overlap between taxa; (11) tadpoles with relatively long barbels and generally fewer small melanophores [50]. In addition, species of *Silurana* are diagnosable by features that require molecular or internal morphological study, reviewed in [12], including a haploid karyotype of 10 chromosomes [51], fusion of the first two presacral vertebrae [11], paired (unfused) nasal bones [11], and absence of the vomer bones in the palate [11], and thus also vomerine teeth (Fig 5).

These subgenera are further distinguished by the parasites they host. For example, the monogenean *Protopolystoma*, the digenean *Dolfuschella*, and the tapeworm *Cephalochlamys* are represented by multiple species in subgenus *Xenopus*, but do not infect species of the subgenus *Silurana*, and the monogenean *Gyrdicotylus* and the digeneans *Oligolecithus* and *Progonimodiscus* each have different species specific to each host subgenus [52]. The camallanid nematodes occur in both host subgenera, but their phylogenetic relationships suggest independent colonization of each [53]. We recognize this clade as the subgenus *Silurana* within the genus *Xenopus* with an aim of promoting consistency with the large body of research focused on the model organism in *Xenopus tropicalis*.

Species within the subgenus *Silurana* are readily distinguished from one another using nucleotide sequences from mitochondrial DNA or autosomal DNA, and by using a combination of karyotype and vocalization data. Below, we describe one new species (*Silurana* new tetraploid 1 *sensu* Evans *et al*. [23]) and for *Silurana* new tetraploid 2 *sensu* Evans *et al*. [23] we resurrect the name *Xenopus calcaratus* Peters [54] from synonymy with *X*. *tropicalis*.

### Species in subgenus *Silurana*

*Xenopus tropicalis* is diploid, whereas the other three species are tetraploid. Two species, *X*. *calcaratus* and the new species described below, are distinguished from *X*. *tropicalis* and *X*. *epitropicalis* by having burst-type calls, with *X*. *calcaratus* being distinguished by having fewer sound pulses (Table 4) [42]. Other spectral features are similar between these two species with burst-type calls including the dominant frequency 1 (both ~500–600 Hz), inter-pulse interval (~3–15 msec), and intensity modulation (~9–19). *Xenopus tropicalis* and *X*. *epitropicalis* are distinguished from the other two species by having, respectively, a trill-type and a biphasic call [42]. The vocalizations of all four species have a similar dominant frequency and all species in subgenus *Silurana* have only one dominant frequency, which is more broadband and lower than species in subgenus *Xenopus* [42]. *Xenopus epitropicalis* is distinguished by having longer interpulse intervals (~22 msec) than the other species (~10 msec); *X*. *tropicalis* is distinguished by higher intensity modulation (~38) compared to the other species (which is ~10, Table 4) [42]. Similar to subgenus *Xenopus*, body size and lateral-lines are insufficient for differentiating species of subgenus *Silurana* (Tables 1–3). Our studies of variation of body size and the number of lateral-line plaques around the eye suggest that these are not useful for diagnosing species of *Silurana* in contrast to previous suggestions [15, 55]. The number of lateral-line plaques around the eye for each species exhibits overlapping variation (Table 3). We provide a detailed description of *X*. *calcaratus* and the new species, and short descriptions regarding the two existing species, *X*. *epitropicalis* and *X*. *tropicalis*.

#### *Xenopus* (*Silurana*) *calcaratus* Peters, 1875

Biafran Clawed Frog

*Silurana* new tetraploid 2 *sensu* Evans et al. (2004)

*Syntypes*.—ZMB 8255 (originally three specimens, only two adult females are known to be present in ZMB–ZMB 8255A and ZMB 74681 (formerly 8255B), ZMB 8326, originally two specimens, one adult female (ZMB 8326A) and one juvenile (ZMB 74682; formerly 8326B), ZMB 8328, originally five specimens, only four specimens are known to be present in ZMB, (ZMB 8328A-D, including one tadpole in metamorphosis), ZMB 8329 (one tadpole in metamorphosis), “Cameruns (Victoria)” [presently Limbe, Southwest Province, Republic of Cameroon], coll. Reichenow; based on Bauer et al. [56], verified and detailed by VG with help of F. Tillack (ZMB). Two syntypes were listed in Zoologischen Museums Greifswald [57], but no further details were provided. To stabilize the identity of the nomen, we designate ZMB 8255A (an adult female, SVL 54 mm) as the lectotype.

*Referred Specimens*.*—*Republic of Equatorial Guinea, Bioko Island: Bioko Norte Province: CAS 207615–19 (N 3.7110° E 8.6666°; ~55 m), 18 October 1998; Bioko Sur Province: Arena Blanca Road: CAS 207752–53 and 207755–58 (N 3.5201°, E 8.5859°; ~65 m), 207759–64 (N 3.5276°, E 8.5794°; ~30 m), 14 October 1998; coll. L. G. Henwood, J. V. Vindum. Republic of Cameroon: South West Region, Bakingili, lava flow: NMP6V 74630/1-4 (two subadults, probably females, and two adult males), 25 November 2009, NMP6V 74746 (adult female), 11 December 2005, (N 4.0689°, E 9.0684°, ~180 m), coll. V. Gvoždík.

*Diagnosis*.—*Xenopus calcaratus* is a tetraploid species with a burst-type call that exhibits all of the morphological features of subgenus *Silurana* described above. Most individuals are a medium to dark brown with a pale interocular bar (more distinct in the Bioko population), and a rostrum tending to darker brown coloration than the rest of the dorsum. Specimens from Bioko Island often exhibit small scattered and irregularly shaped dark brown spots on the dorsum and hindlimbs, which has not been recorded in Cameroonian specimens. *Xenopus calcaratus* differs from other species of *Silurana* in the following ways: from all species by unique nucleotide substitutions in mitochondrial and autosomal DNA (Figs 1 and 2 and S1 and S2); from *X*. *epitropicalis* by having a burst-type instead of a biphasic call [42], having shorter interpulse intervals, and having somewhat less pedal webbing pigmentation; from *X*. *tropicalis* by being tetraploid, having a burst-type instead of a trill-type call and having less intensity modulation in the call; and from the tetraploid new species of *Silurana* described below by fewer pulses in the burst-type call, by less defined lateral-line plaques, and by having large prominent dark brown spots on the dorsum in some specimens.

**Fig 6:**
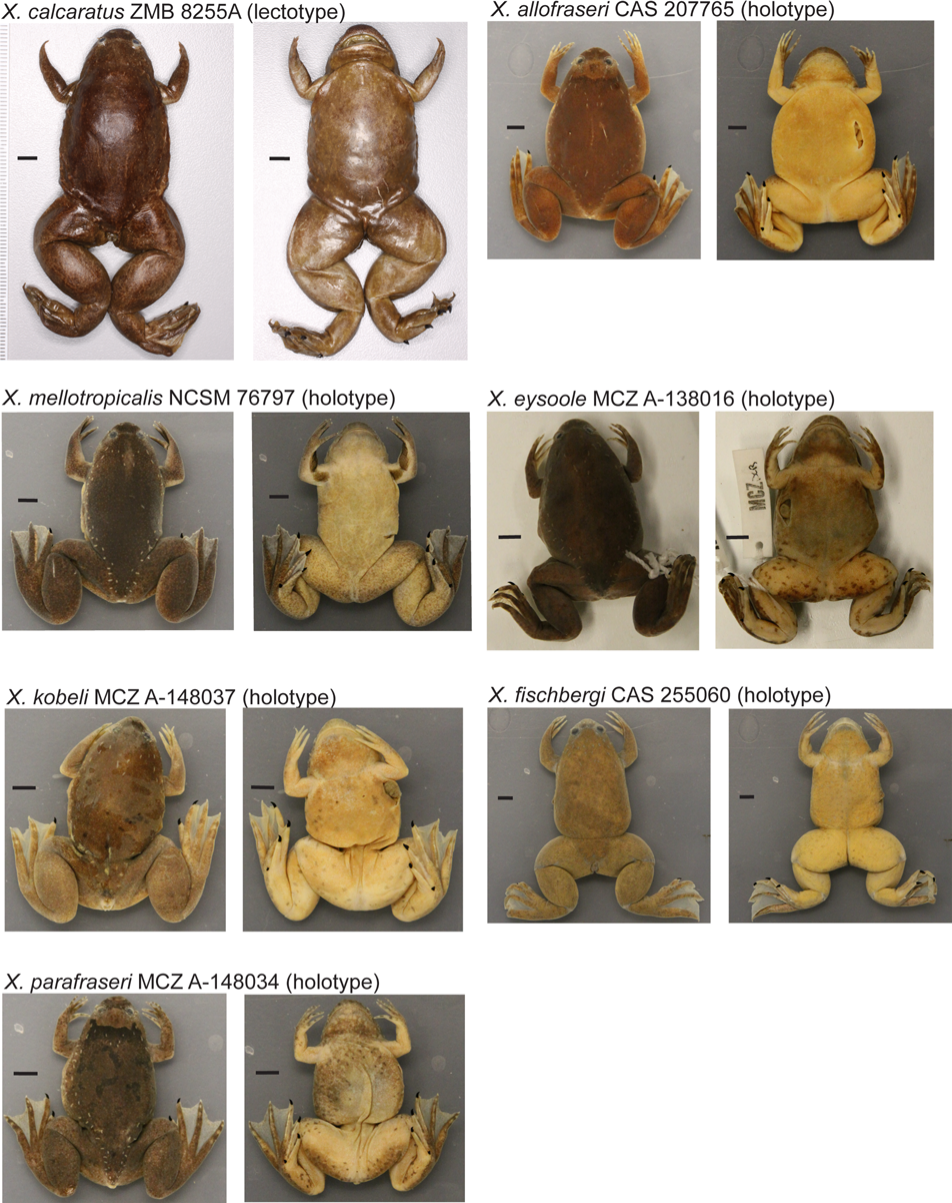
Pictures of holotypes. Pictures of type specimens of resurrected and new species including *X*. *calcaratus* ZMB 8255A (lectotype), *X*. *mellotropicalis* NCSM 76797 (holotype), *X*. *allofraseri* CAS 207765 (holotype), *X*. *eysoole* MCZ A-138016 (holotype), *X*. *kobeli* MCZ A-148037 (holotype), *X*. *parafraseri* MCZ A-148034 (holotype), and *X*. *fischbergi* CAS 255060 (holotype). Scale bar is 5 mm. This is a truncated version of Fig 6 and is meant for preview purposes. Please refer to S3–S5 Figs in the Supporting information for the full size version.

*Description of lectotype* (ZMB 8255A).*—*Proportions were estimated via photographs if no exact values are given in parentheses. Large-sized (SVL 53 mm), moderately robust female (Figs 6 and S3–S5; Table 1; determination of sex based on body size, protruding cloacal lobes, and lacking enlarged posteromedial processes of hyoid plate); rostral tip blunt and slightly squared in dorsal view; eyes weakly projecting beyond margins of orbit in dorsal view and slightly inset from dorsal margins of head in lateral view; subocular tentacle short, length slightly greater than half of eye diameter; eye diameter ~33% of interorbital distance, ~80% of eye–narial distance; internarial distance ~50% of interorbital distance; no vomerine teeth.

Skin generally smooth; small isolated punctiform asperities across dorsal surface of head, body, forelimbs, and hind limbs; few small tubercles on plantar surface; punctiform and closely spaced lateral-line plaques around eye; lateral-line plaques (18 on both sides) most prominent on dorsal and lateral surfaces and extending onto ventral surface; oral and ventral plaques difficult to observe.

*Measurements*.*—*Female specimens reach a maximum SVL of 59 mm (mean: 44 mm; *n* = 19, Table 2), but the size of males remains unclear because of the difficulty in determining sex for most specimens from Bioko. Two likely males from Bioko (CAS 207618, CAS 207756) have SVL of 45.2 and 46.1 mm, respectively. Additional measurements of the lectotype show that the crus (23.1 mm) is longer than the thigh (22.1 mm), the tarsus (16.7 mm), and the 4th toe (measured from the prehallux to toe tip, 15.7 mm).

*Coloration of lectotype (in alcohol)*.*—*Dorsum is medium-dark brown with few distinct spots that are most prominent on the hind limbs (Figs 6 and S3–S5). The darker coloration of the dorsum becomes somewhat paler anteriorly and there is a thin, pale, and indistinct interocular line. The venter ranges from a medium to pale brown, becoming darker near both the gular and inguinal regions; both the forelimbs and hind limbs are paler in coloration than the remaining venter. The lateral-line plaques are generally without pigmentation and thus appear pale in coloration.

**Fig 7:**
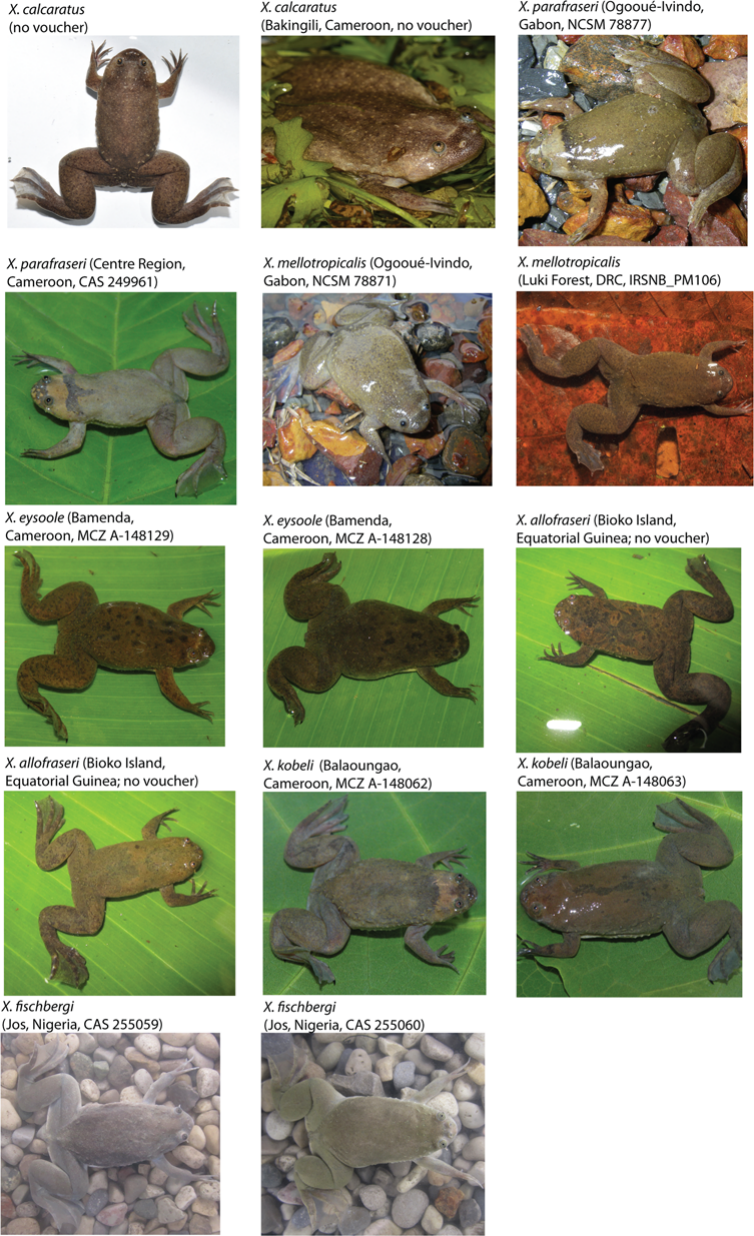
Pictures in life. Pictures of resurrected and new species in life. This is a truncated version of Fig 7 and is meant for preview purposes. Please refer to S6–S8 Figs in the Supporting information for the full size version.

*Coloration in life*.*—*Based on above specimens from mainland Cameroon, *X*. *calcaratus* ranges from medium grayish brown to dark gray, sometimes with dark brown spots on the dorsum (Figs 7 and S6–S8). The interocular bar tends to be pale gray, rather indistinct, and the rostrum is a somewhat darker coloration than the dorsum. The venter and ventral surface of the limbs are pale yellowish gray with indistinct marbling. The posterior parts of the venter, thighs, and throat may be darker in some individuals.

*Variation*.*—*Variation in the lateral-line based on the referred specimens above from Bioko Island (*n* = 17, given as mean and range): orbital– 10 (9–13); oral– 10 (9–12); medial– 16 (15–19); lateral– 18 (15–21); ventral– 17 (14–21) (Table 3).

**Fig 8:**
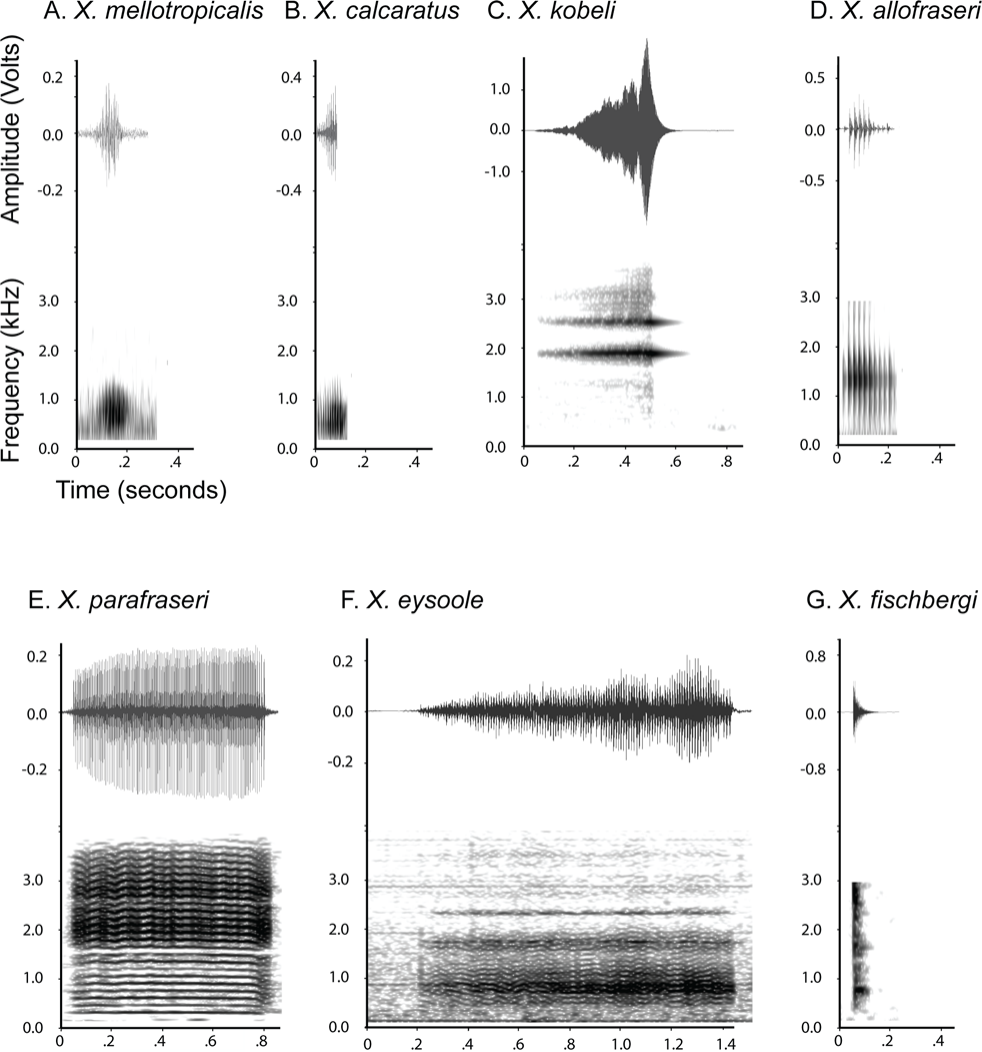
The male vocalizations of resurrected and new species. Axes are labeled only in panel A. For some of the recorded individuals, specimen IDs are available including *X*. *kobeli* (field ID: BJE 3073), *X*. *allofraseri* (MCZ A-148176), *X*. *parafraseri* (CAS 249961), *X*. *eysoole* (MCZ A-148129 or MCZ A-148130), and *X*. *calcaratus* (field ID: VG09-368 or VG09-369).

*Vocalization*.*—Xenopus calcaratus* has a burst-type call (Table 4, Fig 8). Similar to other species of *Silurana* [42], the call of *X*. *calcaratus* has only one dominant frequency.

**Table 4:** Vocal characteristics of the male advertisement call of new species of African clawed frog.

species / locality	Call Type	# calls	# pulses	DF1	DF2	IPI	IM
subgenus *Silurana*							
*X*. *calcaratus*	Burst	2	14.0 (3; 12–16)	675.5 (67; 628–723)	NR	10.0 (0; 10–10)	15.0 (3; 13–17)
*X*. *calcaratus*	Burst	2	7.5 (1; 7–8)	530.5 (13; 521–540)	NR	9.2 (1; 9–10)	6.5 (0; 6–6)
*X*. *calcaratus*	Burst	2	9.0 (3; 7–11)	588.5 (141; 489–688)	NR	9.8 (0; 10–10)	2.6 (2; 1–4)
*X*. *calcaratus*	Burst	4	12.4 (1; 11–14)	543.6 (50; 491–602)	NR	10.1 (0; 10–11)	7.3 (3; 4–12)
*X*. *mellotropicalis* *	Burst	11	83.0	553.0 (22)	744.0 (11)	11.0 (0)	10.0 (4)
*X*. *mellotropicalis* *	Burst	14	130.0	476.0 (4)	724.0 (14)	16.6 (3)	11.6 (6)
*X*. *mellotropicalis* *	Burst	34	297.0	648.0 (24)	NR	15.0 (1)	19.0 (10)
subgenus *Xenopus*							
*amieti* group							
*X*. *allofraseri*	Trill or Burst	13	10.2 (3; 3–10)	1328.3 (13; 1305–1357)	NR	20.4 (2; 18–26)	13.8 (12; 1–37)
*X*. *parafraseri*	Trill	2	77 (50; 42–112)	harmonic stack	NR	7.4 (1; 7–8)	5.4 (6; 1–10)
*X*. *parafraseri* *	Trill	3	381.0	harmonic stack	NR	6.6 (0)	111.9 (54.2)
*X*. *eysoole* / Bamenda	Trill	8	40.7 (39; 11–120)	1798.8 (48.5; 1744–1879)	2362.6 (25.6; 2412–2342)	10.1 (0.2; 10–10)	7.5 (3; 4–12)
*X*. *eysoole* / Nkambe	Burst	2	2.5(2; 1–4)	1634.5 (70; 1585–1684)	2179.0 (134; 2084–2274)	23.9	NR
*X*. *kobeli* / Bangwa (CAS 249997)	Trill	2	56.0 (8; 50–62)	1544.5 (21; 1530–1559)	2048.0 (11; 2040–2056)	18.3 (3; 16–20)	20.9 (4; 18–24)
*X*. *kobeli* / Meganme (field ID: BJE 3073)	Trill	4	29.3 (17; 9–43)	1916 (3; 1920–1912)	2567.5 (3; 2564–2570)	12.5 (6; 9–22)	14.5 (4; 12–21)
*muelleri* group							
*X*. *fischbergi* *	Click	138	1	1635 (8)	2538 (201)	NR	NR
*X*. *fischbergi* *	Click	119	1	1767 (12)	2839 (113)	NR	NR

Each row presents mean values across one or more calls within an individual, and includes the number of calls analyzed (#calls), the number (or average number) of pulses per call (# pulses), the first and second dominant frequencies (DF1 and DF2 respectively), the interpulse interval (IPI), and the intensity modulation (IM). When more than one call was analyzed, the standard devation and range are in parentheses following the average value for each parameter. For some species, calls from multiple localities were analyzed; these localities are indicated after the species name. Quantification of some parameters was not possible or ambiguous, not applicable because the call is a harmonic stack, and/or are not reported (NR). Data for most other species are available in Tobias et al. (2011). For *X*. *allofraseri* there were insufficient data to distinguish between a trill-type or burst-type call, but sufficient information to be sure it was not a click-type call.

* Data from Tobias et al. (2011); no standard deviations available for number of pulses; no ranges are available.

**Fig 9:**
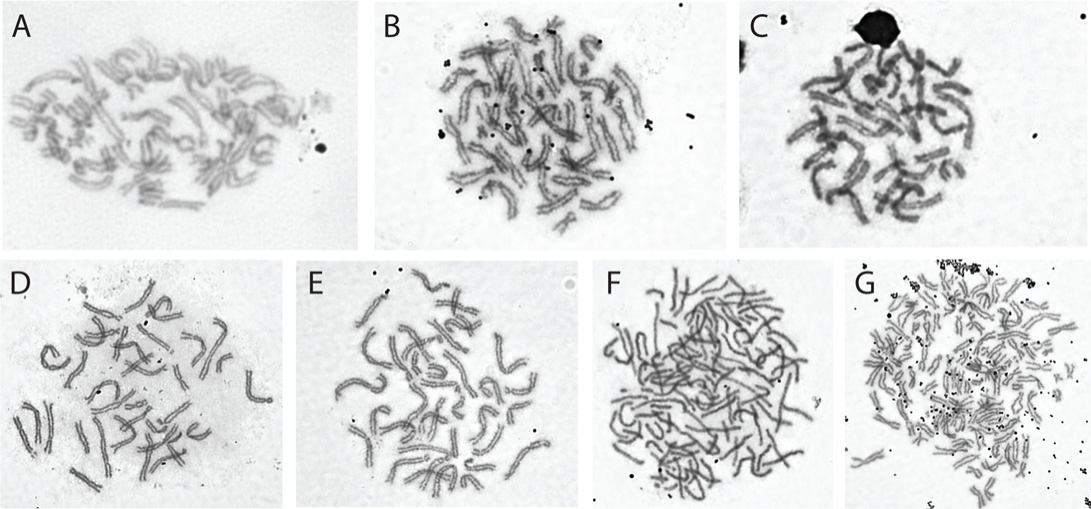
Karyotypes of new and resurrected species. (A) *X*. *calcaratus*, NMP6V 74746 (VG05-S; female) from Cameroon, (B) *X*. *mellotropicalis*, CAS 255058 (BJE 3652) from Republic of Congo, (C) *X*. *fischbergi* non-vouchered sample (BJE 3873), (D) *X*. *parafraseri*, CAS 249961 (BJE 3060) from Cameroon, (E) *X*. *allofraseri*, MCZ A-148162 (BJE 3486) from Bioko Island, Equatorial Guinea, (F) *X*. *kobeli*, MCZ A-148038 (BJE 3076) from Cameroon, (G) *X*. *eysoole*, MCZ A-148097 (BJE 3220), from Cameroon.

*Karyotype*.*—Xenopus calcaratus* is tetraploid with a karyotype of 2*n* = 4*x* = 40 (Fig 9).

**Fig 10:**
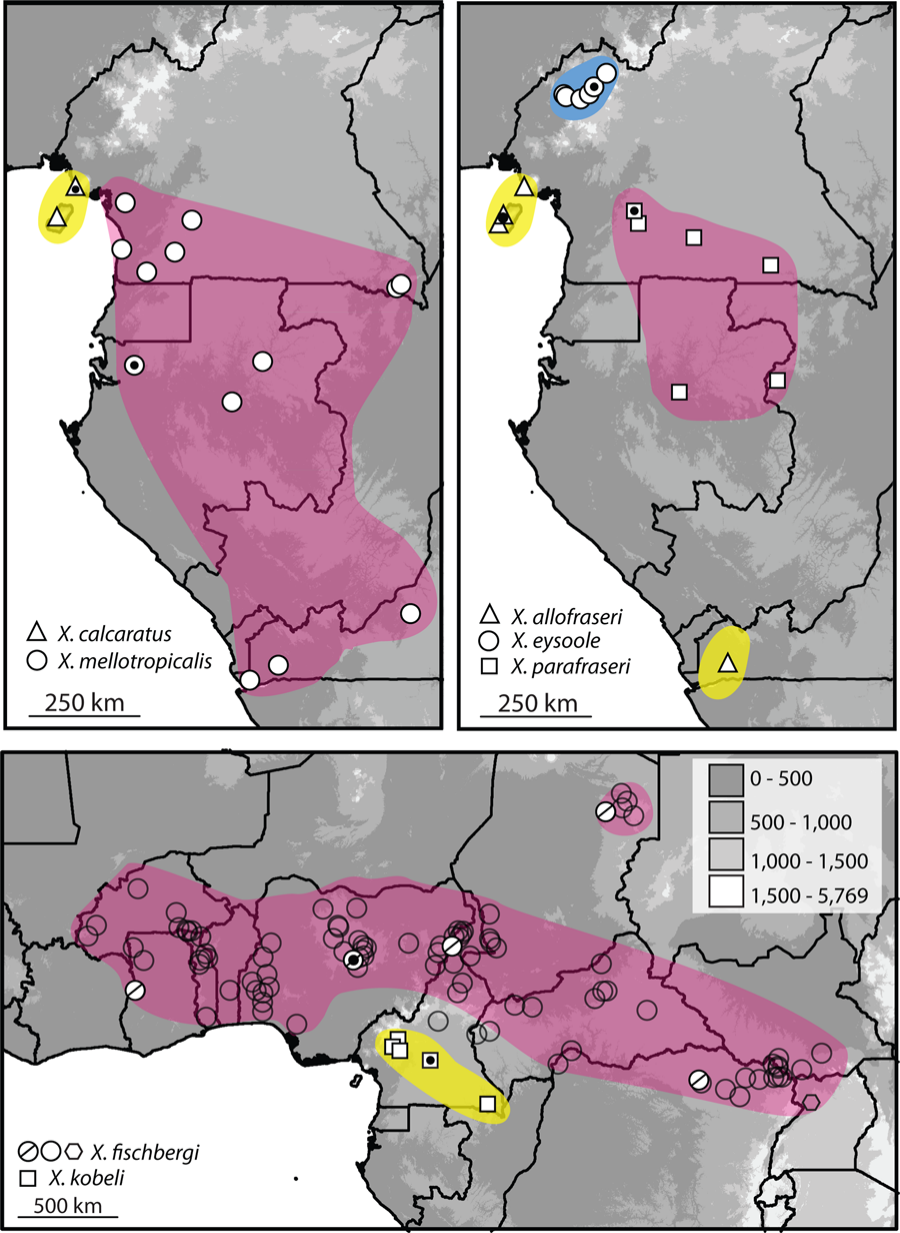
Distributions of new species. Type localities with black dots inside symbols. For *X*. *fischbergi* white circles with slashes indicate specimens from which we have genetic data (including the holotype), unfilled circles are specimens from Tinsley et al. [1] and those other collections from which we lack genetic data, including one field sample from Uganda–indicated by a hexagon–in which the *X*. *fischbergi-*specific parasite *Protopolystoma occidentalis* was detected (J. A. Jackson & RCT, unpublished).

*Habitat and range*.*—*Based on surveys of specimens with genetic data, this species is known only from low elevations on Bioko Island (Equatorial Guinea) and coastal Cameroon near Mt. Cameroon (Fig 10). In portions of both localities, *X*. *calcaratus* can be found syntopically with *X*. cf. *fraseri* 1, sensu [23], which is described as a new species below.

**Fig 11:**
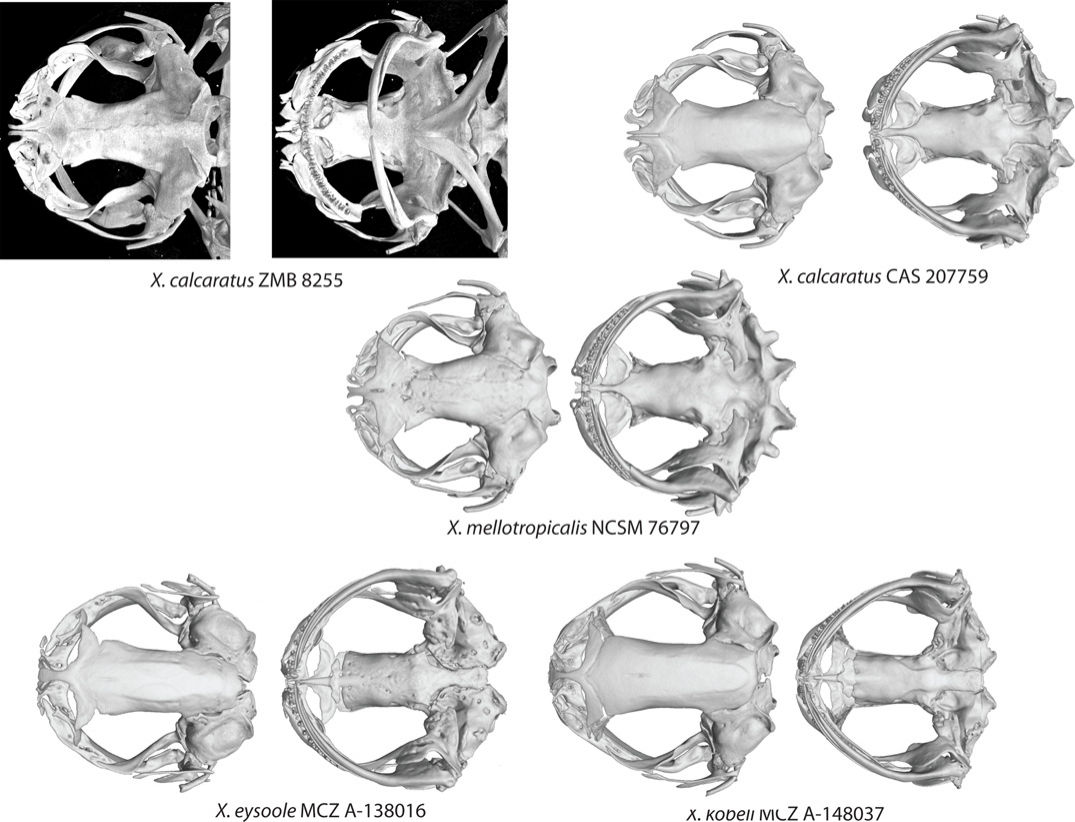
MicroCT scans of skulls of two tetraploids in subgenus *Silurana* and two dodecaploid in subgenus *Xenopus*. Dorsal view is on the left and ventral view is on the right, including the lectotype specimen of *Xenopus* (*Silurana*) *calcaratus* (ZMB 8255A) from Cameroon and a specimen from Bioko Island (CAS 207759), holotype of *X*. (*S*.) *mellotropicalis* (NCSM 76797), and the holotypes of the new dodecaploid species from subgenus *Xenopus*: *X*. *eysoole* (MCZ A-138016) and *X*. *kobeli* (MCZ A-148037). The type specimen of *X*. *calcaratus* was preserved with its mouth ajar.

*Remarks*.—*Xenopus calcaratus* was described by Peters [54] based on material collected at what is now Limbe (formerly Victoria) on the coast of the Republic of Cameroon. Müller [58] suggested that *X*. *calcaratus* should be considered a junior synonym of *X*. *tropicalis* based largely on the assumption that the type specimens of *X*. *tropicalis* represent larvae and metamorphs of the same species as the types of *X*. *calcaratus*. The type locality of *X*. *tropicalis* is Lagos in present-day Nigeria and all evidence suggests that populations of *Silurana* from western Nigeria and farther west in Africa are referable to *X*. *tropicalis* [59]. This tetraploid species found on Bioko Island and coastal Cameroon, including Limbe (Fig 10), is morphologically consistent with the types of *X*. *calcaratus* (Fig 11). Thus, we resurrect *Xenopus calcaratus* [54] from synonymy with *X*. *tropicalis* [49] for this distinct evolutionary lineage.

#### *Xenopus* (*Silurana*) *epitropicalis* Fischberg, Colombelli, & Picard 1982

Congolese Clawed Frog

*Holotype*.*—*BMNH 1982.462, female, Democratic Republic of Congo, “au confluent de la Funa et de la Kemi, à 8 km au sud du centre de Kinshasa (Zaïre),” [confluence of the Funa and Kemi rivers, 8 km south of the centre of Kinshasa (Zaire)]; 350 m; S 4.3° E 15.3°, August 1978, coll. V. Nzingula.

*Paratypes*.—BMNH 1982.463, female, and BMNH 1982.464–65, adult males, same collection information as holotype.

*Diagnosis*.—*Xenopus epitropicalis* is a tetraploid species with a biphasic-type call [42] that exhibits all of the morphological features of subgenus *Silurana* described above. It differs from other species of *Silurana* in the following ways: from all species by unique nucleotide substitutions in mitochondrial and autosomal DNA (Figs 1 and 2 and S1 and S2); from both *X*. *calcaratus* and the new tetraploid described below by having a biphasic call and longer interpulse intervals; and from *X*. *tropicalis* by being tetraploid, lacking a trill-type call, and having less intensity modulation in the call. Characters previously proposed as diagnostic between *X*. *epitropicalis* and *X*. *tropicalis*, including adult body size, number of lateral-line plaques around the eye, and coloration [15, 55] are not useful for distinguishing these two species because of overlapping patterns of variation (Table 3).

*Description of the holotype*.*—*Large-sized (SVL 68 mm), robust female (S9–S14 Figs; Table 1); rostral tip blunt and somewhat rounded in dorsal view; eyes not projecting beyond margins of orbit in dorsal view and projecting slightly beyond dorsal margin of head in lateral view; subocular tentacle short, length less than half eye diameter; eye diameter 37% of interorbital distance, 93% of eye–narial distance, and 1.5 times distance from naris to rostral tip; internarial distance 47% of interorbital distance; no vomerine teeth.

Skin smooth; small prominent asperities on snout and scattered over much of body and limbs, and strongly developed on the forelimbs; many small tubercles across plantar surface; punctiform, globular, and closely spaced lateral-line plaques around eye; lateral-line plaques most prominent on dorsal and lateral surfaces and extending onto ventral surface; oral and ventral plaques projecting from skin; counts of lateral-line rows as follows: orbital– 12, oral– 11, medial– 21, lateral– 23, ventral– 19.

Relative lengths of fingers: II > III ≈ I > IV; relative lengths of fingers when adpressed: II > III ≈ I > IV; thigh length 91% of crus length; relative length of toes: IV ≈ III > II ≈ V > I.

*Measurements*.*—*Female specimens reach a maximum SVL of 68 mm (mean: 56 mm; *n* = 12), and males reach a maximum SVL of 51 mm (mean: 43 mm; *n* = 12) (Table 2). The original description [55] gives the maximum SVL of 72 mm in females and 53 mm in males, both of which measurements are slightly larger than those of the specimens we examined. See S1 Table for more measurements.

*Coloration of holotype (in alcohol)*.*—*Dorsum is grayish medium brown with fine mottling of dark brown across dorsum and hindlimbs, becoming paler on the thigh and inguinal region (S9–S14 Figs). There is a pale gray blotch representing the incomplete interocular line across a darker gray rostrum. The venter and limbs are a uniform dusky gray cream, becoming darker gray towards the head. The plantar surface is uniformly grayish brown. The lateral-line plaques are generally without pigmentation and appear pale in coloration.

*Variation*.*—*Lateral-line counts based on the holotype (*n* = 1): orbital– 12; oral– 11; medial– 21; lateral– 23; ventral– 19 (Table 3).

*Vocalization*.*—Xenopus epitropicalis* has a biphasic call, which is a unique call type in the subgenus, and has longer interpulse intervals (~22 msec) than other species of *Silurana* (~10 msec) [42].

*Karyotype*.*—Xenopus epitropicalis* is tetraploid with a karyotype of 2*n* = 4*x* = 40 [51, 60].

*Habitat and range*.*—*Based on samples with genetic data, *X*. *epitropicalis* is known from near the type locality in Kinshasa, Democratic Republic of Congo, to the northeast of this locality along the Congo River near the confluence with the Kwa River, and from Pointe Noire, Republic of Congo [61], where it was recorded syntopically with the new tetraploid of *Silurana* described below. There are records that may be attributable to *X*. *epitropicalis*, though without genetic data, from northeastern Democratic Republic of Congo [1]. Most previous records of *X*. *epitropicalis*, including from Cameroon and Gabon [1], most likely represent the new tetraploid species of *Silurana* described below.

*Remarks*.*—*Mitochondrial DNA sequences from our *X*. *epitropicalis* sample (S2 Table) are derived from the lab colony in Geneva from which the species was described.

#### *Xenopus* (*Silurana*) *mellotropicalis*, new species

urn:lsid:zoobank.org:act:EA07F9C0-4426-464E-8785-055EB3893138

Gabonese Clawed Frog

*Xenopus “species nova VII” sensu* Graf & Fischberg [62]

*Xenopus* sp. nov. VII *sensu* Tymowska [51]

*Silurana paratropicalis* [nomen nudum] *sensu* Flajnik et al. [63], Sato et al. [64], Shum et al. [65], Herrmann [66], Salamone [67], Salamone & Montoya-Burgos [68], and Mecharksa *et al*. [69]

*Silurana* new tetraploid 1 *sensu* Evans et al. [23]

*Holotype*.*—*NCSM 76797 (field no. BLS 13506), male, Gabonese Republic, Estuaire Province, Monts de Cristal National Park, Kinguele, N 0.4536°, E 10.2781°, 75 m, 8 October 2009, coll. B. L. Stuart, R. C. Bell, P. Minko, T. Essone.

*Paratypes*.—Gabonese Republic: NCSM 78871, adult female, Ogooué-Ivindo Province, Rougier Gabon Forestry Concession, N 0.2018 E 12.2693, 221 m, 16 October 2011, coll. B. L. Stuart, R. C. Bell, T. Ogombet, U. Eyagui, P. Endazokou; NCSM 78881, juvenile, same collectors, N 0.0426 E 12.2983, 21 October 2011. Republic of Cameroon: Centre Region: NMP6V 74568, adult female, Ebogo, N 3.3913 E 11.4663, 628 m, 17 October 2009, coll. V. Gvoždík; Est Region: NMP6V 74718, subadult female, Kika, N 1.9419° E 15.6269°, 337 m, 30 May 2010, coll. V. Gvoždík, O. Kopecký; Sud Region: MHNG 2644.58 (AMNH 17288), male laboratory animal descended from animals collected in Nkoemvone, N 2.8800° E 11.1500°, ~575 m, unknown date, coll. H. R. Kobel; ZFMK 87790–1, sex unknown, Nkoelon, Campo Region, N 2.3500° E 10.6167°, 76 m, October 2007, coll. M. Barej, J. Wurstner; Democratic Republic of Congo: CAS 250558, female, Bas-Congo Province, Malemba, S 5.83°, E 12.57° (estimated), ~150 m, collection date unknown, coll. D. Rungger (specimen from captive population originally housed in Laboratoire de Génétique Animale et Végétale, Université de Genève; possibly wild caught).

*Referred Specimens*.—Democratic Republic of Congo: Bas-Congo Province, IRSNB (PM106), probably male, Luki Reserve, S 5.5963° E 13.1603°, ~220 m, 19 June 2012, IRSNB (PM119), male, Tsumba-Kituti, S 5.6581° E 13.1995°, ~255 m, 20 June 2012, coll. V. Gvoždík, Z. C. Kusamba, M. M. G. Collet, Z. T. Nagy.

*Diagnosis*.—*Xenopus mellotropicalis* is a tetraploid species with a burst-type call that exhibits all of the morphological features of subgenus *Silurana* described above (Fig 11). It differs from other species of *Silurana* in the following ways: from all species by unique nucleotide substitutions in mitochondrial and autosomal DNA (Figs 1 and 2 and S1 and S2); from *X*. *calcaratus* by more pulses in the call, more defined lateral-line plaques, and generally lacking prominent dark spots on the dorsum (common in the Bioko population of *X*. *calcaratus*); from *X*. *epitropicalis* by lacking a biphasic call, and having shorter interpulse intervals; from *X*. *tropicalis* by being tetraploid, by lacking a trill-type call and having less intensity modulation in the call, and generally lacking prominent dark spots on the dorsum. *Xenopus mellotropicalis* also differs from *X*. *tropicalis* and *X*. *epitropicalis* by the peptides present in its norepinephrine-stimulated skin secretions [70].

*Description of the holotype*.*—*Medium-sized (SVL 48 mm), moderately robust male (Table 1, Figs 6 and S3–S5); rostral tip blunt and rounded in dorsal view; eyes not projecting beyond margins of orbit in dorsal view and projecting slightly beyond dorsal margin of head in lateral view; subocular tentacle short, length less than half eye diameter; eye diameter 42% of interorbital distance, 83% of eye–narial distance, and 1.4 times distance from naris to rostral tip; internarial distance 46% of interorbital distance; no vomerine teeth.

Skin smooth; small prominent asperities on snout and scattered over much of body and limbs; small tubercles across plantar surface; punctiform but well separated lateral-line plaques around eye; lateral-line plaques most prominent on dorsal and lateral surfaces and extending onto the ventral surface, and oral and ventral plaques difficult to observe due to similarity of coloration with venter; counts of lateral-line rows as follows: orbital– 11, oral– 12, medial– 17, lateral– 17, ventral– 13; male nuptial pads are well developed appearing as dark keratinous patches on ventral surface of the arm and forearm, and extending along metacarpals and digits.

Relative lengths of fingers: II ≈ III > IV > I; relative lengths of fingers when adpressed: II > III > IV > I; thigh length 111% of crus length; relative length of toes: IV > V ≈ III > II > I; foot and toes along metatarsals and digits with scattered prominent pustules on ventral surfaces.

*Measurements*.*—*Female specimens reach a maximum SVL of 58 mm (mean: 53 mm; *n* = 14), and males reach a maximum SVL of 51 mm (mean: 45 mm; *n* = 12) (Table 2). See S1 Table for more measurements.

*Coloration of holotype (in alcohol)*.*—*Dark brownish gray on dorsum and limbs (Figs 7 and S6–S8). The darker coloration of the dorsum is somewhat paler on the anterior; there is a thin pale gray interocular line; the coloration anterior and posterior to the interocular line is similar. There are no prominent spots on the dorsum, but the pattern tends to be finely variegated. The venter is orange-gray and darker gray towards the head; variegations on the ventral thighs are darker than those on the venter.

*Coloration in life*.*—*Based on color photographs of NCSM 78871 (Fig 8), the dorsum of *X*. *mellotropicalis* is pale greenish and grayish brown in life with scattered medium variegations. The venter is a pale gray with hints of pale yellow blotches, especially posteriorly and on the ventral hind limbs.

*Variation*.*—*Variation in the lateral-line (*n* = 4, given as mean and range): orbital– 11.0 (10–12); oral– 11 (10–13); medial– 17 (16–19); lateral– 19 (16–19); ventral– 14 (10–18 (Table 3).

*Vocalization*.*—Xenopus mellotropicalis* has a burst-type call Table 4, Fig 8 [42].

*Karyotype*.*—Xenopus mellotropicalis* is tetraploid with a karyotype of 2*n* = 4*x* = 40, Fig 9 [60].

*Habitat and range*.*—*Based on surveys of specimens with genetic data, *X*. *mellotropicalis* is found in both disturbed and forested areas in Central Africa, including the Congo Republic [61], Cameroon, Gabon, and Democratic Republic of Congo (Fig 10). It is likely also found in mainland Equatorial Guinea and southwestern Central African Republic. It lives in sympatry with several *Xenopus* species, including *X*. *epitropicalis* in the Republic of Congo [61], as well as *X*. cf. *fraseri* 1, and *X*. cf. *fraseri* 2 sensu [23] in DRC and Cameroon, respectively; see below for descriptions of the latter two species.

*Etymology*.*—*For the species epithet, we have combined the Greek word *μέλλω* (mello), often interpreted as indicating that something is “about to” happen [71], to *tropicalis*, which forms part of the specific epithet of the other two other species in the subgenus *Silurana* (*X*. *tropicalis* and *X*. *epitropicalis*). This species epithet, which is an adjective, suggests the long delay in a formal description of this species that has been referenced in the literature for nearly thirty years beginning with Graf & Fischberg [62]. In 1993, a series of publications referred to this species as “*Xenopus paratropicalis*,” or “*Silurana paratropicalis*” but this is not a valid name, for review see [72]

*Remarks*.*—*In addition to wild-caught individuals with associated genetic data, we also have DNA sequence from CAS 250558, a specimen from the laboratory colony established in Geneva. Similarity to recently collected specimens confirms that previous authors have in fact published on the same entity that we describe here as *X*. *mellotropicalis*.

#### *Xenopus* (*Silurana*) *tropicalis* Gray 1864

Tropical Clawed Frog

*Syntypes*.*—*BMNH 1947.2.24.83–86, metamorphs and tadpoles, “West Africa, Lagos,” now Federal Republic of Nigeria, coll. R.B.N. Walter. To stabilize the identity of the nomen, we designate BMNH 1947.2.24.83 (a late stage metamorph) as the lectotype.

*Diagnosis*.—*Xenopus tropicalis* exhibits all of the morphological features of subgenus *Silurana* described above, and is diagnosable from other species in the subgenus by unique nucleotide substitutions in mitochondrial and autosomal DNA (mitochondrial DNA is paraphyletic; Figs 1 and 2 and S1 and S2), by having a trill-type call, and by being the only diploid in the genus. In addition, it differs from other species of *Silurana* by having higher intensity modulation (~38) of its call in contrast to other species (which is ~10), Table 4, [42]. As noted above, characters previously proposed as diagnostic between *X*. *epitropicalis* and *X*. *tropicalis*, including adult body size, number of lateral-line plaques around the eye, and coloration (Fischberg *et al*., 1982; Kobel *et al*., 1996), are not useful for distinguishing these two species.

*Comments on syntypes*.*—*The four syntypes are two late-stage tadpoles and two metamorphs in stages corresponding to NF 57–60 based on the staging developed by Nieuwkoop and Faber [73] for *X*. *laevis* (S9–S14 Figs). Two specimens, BMNH 1947.2.24.84 and 86 are incomplete due to damage of the posteriormost tail (respectively, NF 59 at 46 mm total length, and NF 58 at 37 mm total length). The latest stage individual (BMNH 1947.2.24.83) is at NF 59/60 with a total length of 54 mm, whereas the earliest stage specimen (BMNH 1947.2.24.85) has a total length of 63 mm. As indicated in the original description, these larval specimens have elongate barbels as is typical of species in the subgenus *Silurana*. The coloration of the latest stage individual (BMNH 1947.2.24.83) is medium brown with scattered darker brown variegations (S9–S14 Figs), with a venter that is pale creamy beige and a small stark white region at the opercular region. The specimen lacks pigmentation on the pedal webbing and has three dark keratinous pedal claws as well as a similar claw on the prehallux. The other specimens are largely consistent in appearance, and all appear to have faded in coloration over time. We designate the latest stage individual BMNH 1947.2.24.83 as the lectotype.

*Variation*.*—*Variation in the lateral-line based on specimens from across the geographic range of this species in West Africa (*n* = 15, given as mean and range): orbital– 10 (8–12); oral– 11 (9–12); medial– 18 (16–21); lateral– 20 (17–22); ventral– 18 (16–21).

*Vocalization*.*—Xenopus tropicalis* has a trill-type call with higher intensity modulation than in other species [42].

*Karyotype*.*—Xenopus tropicalis* is the only diploid species of *Xenopus* with a karyotype of 2*n* = 20 [51, 60].

*Habitat and range*.*—*Based on samples with available genetic data, *X*. *tropicalis* is widespread across West Africa, extending from Sierra Leone east to at least into western Cameroon [1, 59]. We lack genetic data for samples from central Cameroon, though previously Tinsley *et al*. [1] recognized the easternmost extent of this species as the Sanaga River.

### Subgenus *Xenopus* Wagler, 1827 [44]

Previous authors divided *Xenopus* into subgroups including the *laevis* subgroup (*X*. *laevis* sensu lato, *X*. *gilli*, *X*. *largeni*), the *muelleri* subgroup (*X*. *muelleri*, *X*. *borealis*, *X*. *clivii* and an undescribed species *X*. “new tetraploid” / *X*. “*muelleri* west” that is described below), the *fraseri* subgroup (*X*. *fraseri*, *X*. *pygmaeus*, *X*. *amieti*, *X*. *andrei*, *X*. *boumbaensis*, *X*. *ruwenzoriensis*), the *vestitus*-*wittei* subgroup (*X*. *vestitus*, *X*. *wittei*), and *longipes* subgroup (*X*. *longipes*) [15]. Phylogenetic analyses reported here and elsewhere, for example [16, 21] facilitate redefinition of these groups based on common ancestry (Figs 1–3 and S1 and S2). Apart from the monotypic *longipes* subgroup, monophyly is not strongly supported for any of these subgroups by either mitochondrial DNA or autosomal DNA. Monophyly of the *muelleri* subgroup is supported by autosomal DNA, but not mitochondrial DNA. Paraphyly of the *fraseri* and *vestitus*-*wittei* subgroups *sensu* Kobel et al. [15] is attributable to the reticulating evolutionary history of the allopolyploid species they contain. Uncertainty in the phylogenetic placement of *X*. *largeni* (Figs 2 and 3) means that monophyly is uncertain for the *laevis* subgroup *sensu* Kobel et al. [15].

We modify those traditional groupings in light of phylogenetic discoveries as well as recently described species. The groups that we recognize within the subgenus *Xenopus* are (1) the *amieti* species group (*X*. *amieti*, *X*. *andrei*, *X*. *boumbaensis*, *X*. *itombwensis*, *X*. *lenduensis*, *X*. *longipes*, *X*. *pygmaeus*, *X*. *ruwenzoriensis*, *X*. *vestitus*, *X*. *wittei*, and three new species described below), (2) the *laevis* species group (*X*. *gilli*, *X*. *laevis*, *X*. *petersii*, *X*. *poweri*, and *X*. *victorianus*; see Furman et al. [2]), and (3) the *muelleri* species group (*X*. *borealis*, *X*. *muelleri*, a new species described below, and possibly also *X*. *clivii*). The relationships of *X*. *fraseri* and the Ethiopian endemic *X*. *largeni* remain uncertain and we therefore do not assign them to a species group. The subgenus *Xenopus* can be differentiated from the four species in the subgenus *Silurana* by a number of morphological features (see above and Figs 4 and 5). Interestingly, patterns of parasite specificity match the species groups within *Xenopus* in that species of several parasite genera exclusively infect host species in either the *laevis*, *amieti*, or the *muelleri* species groups [74, 75]. For several parasites (including the monogenean *Protopolystoma* and the digenean *Dolfuschella*), there are significant morphometric and life cycle differences between samples from different parts of the geographical range of *X*. *laevis* sensu lato [75] that match phylogenetic divisions within this clade [2]. Within the *muelleri* species group, there are distinct species of *Protopolystoma*, *P*. *occidentalis* and *P*. *orientalis*, that are respectively host specific to *X*. *muelleri* and the new tetraploid species in this group described below [75, 76].

### Species in subgenus *Xenopus*

#### *Amieti* species group

The *amieti* species group comprises 14 species found across Central Africa, from Nigeria in the west to Uganda and Rwanda in the east, including three new species described below. Some species of this group are distinguished by being octoploid and dodecaploid (no other species group has species with these ploidy levels). Previously this group was referred to as the *fraseri* species group [15], but the phylogenetic affinities of *X*. *fraseri* remain uncertain (see below). We therefore do not recognize the *fraseri* species group and instead propose the *amieti* species group to include all of the species currently in the *fraseri* species group except *X*. *fraseri*, which we do not place in a species group for the time being.

We additionally place the *longipes* species group (*sensu* Kobel *et al*., 1996), which includes only *X*. *longipes*, into the *amieti* species group based on inferred evolutionary relationships [16, 20–23]. Because phylogenetic relationships among the tetraploid ancestors of some of the octoploid and dodecaploid species in the *amieti* species group also include ancestors of species (*X*. *vestitus*, *X*. *wittei*, *X*. *itombwensis*, and *X*. *lenduensis*) [16, 20–22] that were previously placed in the *vestitus-wittei* group [15], we place all of these species in an expanded *amieti* species group to reflect this shared evolutionary history.

In general, these medium to small-sized *Xenopus* species can be identified by the following combination of external morphological features: (1) unfused cloacal lobes; (2) prominent keratinous claw on the prehallux; (3) a skin ridge extending along the first pedal digit from the prehallux; (4) dorsal skin often with small spicules. The four species comprising what was previously recognized as the *vestitus*-*wittei* group [15] are distinguished by lacking the claw on the prehallux that is found in all other species in the *amieti* group. While unreceptive females in other species of subgenus *Xenopus* produce a release call when clasped by a male, females in the *amieti* species group do not [43].

#### *Xenopus (Xenopus) allofraseri*, new species

urn:lsid:zoobank.org:act:8A71C6BE-612D-4ECD-A5CE-0B31AC38E9AE

False Fraser’s Clawed Frog

*Xenopus* cf. *fraseri* 1 *sensu* Evans et al. (2004)

*Xenopus fraseri*-like Tinsley et al. [1]

*Holotype*.*—*CAS 207765 (field no. RCD 13495), female, Republic of Equatorial Guinea, Bioko Island, Bioko Sur Province, Arena Blanca road, N 3.5275°, E 8.5793°, ~30 m, 14 October 1998, coll. L. G. Henwood, J. V. Vindum (Figs 6 and S3–S5).

*Paratypes*.*—*CAS 207766–70, all probably females, same collection data as holotype. MCZ A-148161, 148163–64, 148169–71, 148173, 148175, 148177–78, females, Republic of Equatorial Guinea, Bioko Island, Bioko Sur Province, Comedor, N 3.3375° E 8.5873°, 1218 m, 11 November 2011, coll. B. J. Evans, I. M. Mohete; MCZ A-148162, 148166–68, 148172, 148174, 148176, males, same locality data.

*Referred Specimens*.*—*Republic of Cameroon: South-West Region, Bakingili, lava flow, N 4.0689°, E 9.0684°, ~180 m: NMP6V 73406/1–8, 74747/1–2, six males, three females, one juvenile, 11 December 2005; NMP6V 74627/1–5, three males, two females, 25 November 2009, coll. V. Gvoždík. Democratic Republic of Congo: Bas-Congo Province, IRSNB (PM107, 110, 113, 114), 3 males, 1 female, Luki Reserve, S 5.5963° E 13.1603°, ~220 m, 19 June 2012, coll. V. Gvoždík, Z. C. Kusamba, M. M. G. Collet, Z. T. Nagy.

**Fig 12:**
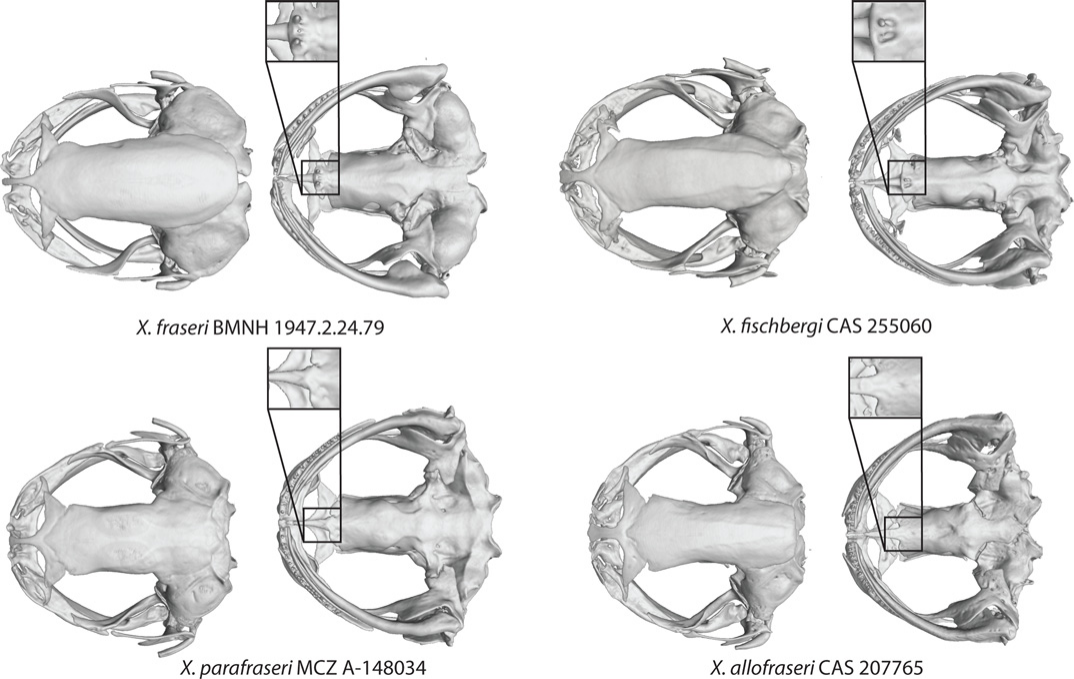
MicroCT scans of skulls of four tetraploids in subgenus *Xenopus*. Images are of a type specimen of *X*. *fraseri* (BMNH 1947.2.24.79) and holotypes of the new tetraploid species from subgenus *Xenopus*: *X*. *fischbergi* (CAS 255060), *X*. *allofraseri* (CAS 207765), *X*. *parafraseri* (MCZ A-148034). Each ventral view (on right for each species) and insert shows the presence of vomerine teeth (*X*. *fraseri* and *X*. *fischbergi*) or absence of vomerine teeth (*X*. *allofraseri* and *X*. *parafraseri*).

*Diagnosis*.*—Xenopus allofraseri* is a tetraploid species with mitochondrial and autosomal DNA that possesses unique nucleotide substitutions different from all other species (Figs 1 and 2 and S1 and S2) and a trill-type or burst-type call. *Xenopus allofraseri* is distinguished from the closely related tetraploid species *X*. *pygmaeus* because the latter is smaller and its call has a higher first dominant frequency. Another closely related tetraploid species–the new tetraploid species below–has a call with higher dominant frequencies (DF1 and DF2) (Table 4). In addition, the new tetraploid species described below is not known from low elevations (< 400 m) whereas *X*. *allofraseri* occurs above and below 400 m (S2 Table). *Xenopus allofraseri* differs from the other closely related species such as *X*. *longipes* and *X*. *pygmaeus* by being larger (Table 2), and having a trill-type or burst-type call in contrast to the biphasic-type call of *X*. *itombwensis*. Because *X*. *allofraseri* is tetraploid, it is differentiable from the new dodecaploid species described below. The presence of a prehallux claw in *X*. *allofraseri* further differentiates it from *X*. *itombwensis*, *X*. *lenduensis*, *X*. *vestitus*, and *X*. *wittei*. Both *X*. *allofraseri* and the other new tetraploid species described below were previously referred to as “*X*. *fraseri*-like” [1], yet, in contrast to *X*. *fraseri*, both new species lack vomerine teeth, Fig 12, see discussion of the syntypes below, [47].

*Description of the holotype*.*—*Medium-sized (SVL 48 mm), moderately robust female (Table 1, Figs 6 and S3–S5); rostral tip rounded in dorsal view; eyes projecting just beyond margins of orbit in dorsal view and beyond dorsal margin of head in lateral view; prominent subocular tentacle, length more than half eye diameter and extending nearly to upper lip; eye diameter 38% of interorbital distance, 82% of eye–narial distance, and approximately equal distance from naris to rostral tip; internarial distance 40% of interorbital distance; vomerine teeth absent.

Skin smooth and lacking asperities; lateral-line plaques most prominent on dorsal and lateral surfaces and extending onto the ventral surface; both horizontally and vertically oriented lateral-line stitches well-defined on dorsum and lateral surfaces of body, more difficult to discern on ventral surface except when darker venter coloration present.

Relative lengths of fingers: II > III ≈ IV > I; relative lengths of fingers when adpressed: II > III > I > IV; thigh length approximately equal to crus length; relative length of toes: IV > III > V > II > I; few scattered asperities on plantar surface.

*Measurements*.*—*Female specimens reach a maximum SVL of 48 mm (mean: 34 mm; *n* = 17), and males reach a maximum SVL of 38 mm (mean: 35 mm; *n* = 6) (Table 2). See S1 Table for more measurements.

*Coloration of holotype (in alcohol)*.*—*Dorsum medium brown with prominent and well defined pale creamy brown region extending from midpoint of eyes to posterior skull (Figs 6 and S3–S5). Neuromast “stitches” are unpigmented and distinct against dorsal coloration. Venter pale orange, and lacking dark melanocytes or spots on venter or ventral limbs. Pedal webbing unpigmented.

*Coloration in life*.*—*Based on color photographs of unvouchered specimens from Bioko Island (Figs 7 and S6–S8), the dorsum and limbs of *Xenopus allofraseri* range from medium to pale grayish brown with medium to dark brown markings. The Cameroonian specimens range from lacking pattern to sometimes patterned with dark brown markings. The venter is pale with yellowish marbling on the underside of the hind limbs. In the population from western Democratic Republic of the Congo, there is sometimes a pale bar behind the eyes that is bordered posteriorly by a darker coloration.

*Variation*.*—*Variation in the lateral-line (*n* = 18 except where noted, given as mean and range): orbital– 13 (11–15); oral– 13 (10–15); medial– 16 (13–17); lateral– 20 (18–23); ventral– 17 (11–15; *n* = 2).

*Vocalization*.*—Xenopus allofraseri* has a trill-type call (Table 4, Fig 8).

*Karyotype*.*—Xenopus allofraseri* is tetraploid with a karyotype of 2*n* = 4*x* = 36 (Fig 9).

*Habitat and range*.*—*Based on surveys of specimens with genetic data, *Xenopus allofraseri* occurs in disturbed and forested areas on Bioko Island and along the Atlantic coastal region of Cameroon and the Democratic of Republic of Congo (Fig 10). This species was found syntopically with *X*. *calcaratus* (Cameroon) and *X*. *mellotropicalis* (DRC).

*Etymology*.*—*For the species epithet, we have appended the Greek work *ἄλλος* (allos), meaning “other”, to *fraseri*. This taxon name, *Xenopus allofraseri*, maintains the historical taxonomic relationship of these populations to the taxon *X*. *fraseri* while recognizing that they are not the same evolutionary lineage.

#### *Xenopus (Xenopus) eysoole*, new species

urn:lsid:zoobank.org:act:D15EA055-1351-4AAC-92B3-BE546FF05C64

Bamiléké Clawed Frog

*Holotype*.*—*MCZ A-138016 (DCB 34586), adult female, Republic of Cameroon, North-West Region, Elak Oku village, southern face of Mt. Oku, N 6.2427º, E 10.5011º, 1870 m, 16 August 2006, D. C. Blackburn, K. S. Blackburn, P. Huang, and M. T. Kouete (Figs 6 and S3–S5).

*Paratypes*.*—*MCZ A-138017, adult female, same collection data as holotype. Republic of Cameroon: North-West Region: MCZ A-148094–5, female and male, 5 km east of Nkambe, N 6.5486° E 10.7600°, 1684 m, 29 October 2011, coll. B. J. Evans, D. M. Portik, S. B. Menzepoh; MCZ A-148127-8, MCZ A-148131, females, Bamenda fish pond farm, N 6.0143° E 10.2703°, 1441 m, 2 November 2011; MCZ A-148129-30, males, same locality data, coll. B. J. Evans, D. M. Portik, S. B. Menzepoh; NMP6V 74555, adult female, Babungo, N 6.0488° E 10.4259°, 1771 m, 22 December 2009, coll. R. Tropek, A. Kodádková; NMP6V 74745/1-2, females, Njikwa, Acha Tugi Mountains, N 6.0618° E 9.9158°, 1800 m, 23 January 2012, coll. R. Tropek, D. Leština.

*Diagnosis*.*—Xenopus eysoole* is a dodecaploid species, with either a trill-type or burst-type call, that exhibits all of the morphological features of the *amieti* species group described above. Phylogenetic analysis of mitochondrial and autosomal data (Figs 1 and 2 and S1 and S2) suggests this species may be the sister-species of *Xenopus longipes* (based on close phylogenetic relationships of mitochondrial DNA and homeologs α1, β1, α3, and β3), or that these two species have a more complicated but intermingled evolutionary history (based on a close phylogenetic relationship between homeolog α2 of *X*. *eysoole* and *X*. *amieti*). See Discussion for possible explanations for this and other phylogenetic inferences. *Xenopus eysoole* differs substantially from *X*. *longipes* by larger body size (e.g., maximum female SVL we measured is 34 mm in *X*. *longipes* and 52 mm in *X*. *eysoole*), having fused nasals–unfused in *X*. *longipes* [15], transverse processes of the fourth presacral vertebra that are curved posteriorly–uncurved in *X*. *longipes* [15], relatively smaller otic capsules–large in *X*. *longipes* [15], and a creamy white ventral coloration in contrast to the yellow or orange in *X*. *longipes*. *Xenopus longipes* further differs from *X*. *eysoole* in that many individuals have clumps of melanophores on the ventral surface, sometimes forming an anastomosing network, and less well-defined lateral-line stitches. In addition, *Xenopus eysoole* differs from the other species within the *amieti* species group by having a larger body size than *X*. *pygmaeus* and longer interpulse intervals than in *X*. *amieti* and *X*. *lenduensis*, and lower dominant frequencies than *X*. *ruwenzoriensis* and *X*. *amieti*. The presence of a prehallux claw in *X*. *eysoole* further differentiates it from *X*. *itombwensis*, *X*. *lenduensis*, *X*. *vestitus*, and *X*. *wittei*.

*Description of the holotype*.*—*Medium-sized (SVL 39 mm), moderately robust female (Table 1, Figs 6 and S3–S5); rostral tip blunt and rounded in dorsal view; eyes not projecting beyond margins of orbit in dorsal view and flush with dorsal margin of head in lateral view; subocular tentacle short, length less than half eye diameter; eye diameter 40% of interorbital distance, 87% of eye–narial distance, and 1.1 times distance from naris to rostral tip; internarial distance 40% of interorbital distance; no vomerine teeth (Fig 11).

Skin smooth; small asperities on snout and scattered over much of body and limbs (most evident when dry); lateral-line plaques prominent on head and dorsal and lateral surfaces; both horizontally and vertically oriented lateral-line stitches on dorsum well defined.

Relative lengths of fingers: III > IV > II > I; relative lengths of fingers when adpressed: III > II > I > IV; thigh length 93% of crus length; relative length of toes: IV > III > V > II > I; foot, but not toes, with many prominent pustules on ventral and lateral surfaces.

*Measurements*.*—*Female specimens reach a maximum SVL of 52 mm (mean: 50 mm; *n* = 7), and males reach a maximum SVL of 41 mm (mean: 40 mm; *n* = 3). See S1 Table for more measurements.

*Coloration of holotype (in alcohol)*.*—*The dorsum and limbs are a medium grayish brown and lacking prominent markings (Figs 6 and S3–S5). The venter is pale grayish cream with scattered orange and gray spots on the ventral thighs.

*Coloration in life*.*—*The dorsum and limbs of *Xenopus eysoole* is grayish brown with shades of green (Figs 7 and S6–S8); based on DCB’s field notes and photographs, the venter is a creamy white.

*Variation*.*—*Variation in the lateral-line based on type specimens at MCZ (*n* = 13 except where noted, given as mean and range): orbital– 13 (10–14); oral– 11 (9–13); medial– 20 (17–23); lateral– 20 (18–23); ventral– 16 (13–18, *n* = 4).

*Vocalization*.*—Xenopus eysoole* has either a trill-type or burst-type call (Table 4, Fig 8). These two call types may represent variation across populations, or differences in the social context or reproductive state when the calls were recorded.

*Karyotype*.*—Xenopus eysoole* is dodecaploid with a karyotype of 2*n* = 12*x* = 108 (Fig 9).

*Habitat and range*.*—Xenopus eysoole* is known from high elevations (~1400–2000 m) in the northern part of the Bamiléké Plateau in Cameroon, in the Bamenda-Banso Highlands (Fig 10). The type specimens were found in a small pool (approximately 1 m^2^; approximately 20 cm deep). This pool was located several meters from a fast-moving stream running through cultivated land in Elak Oku village. *Astylosternus rheophilus*, *Cardioglossa pulchra*, *C*. *schioetzi*, and *Phrynobatrachus steindachneri* [sensu 77] were found in a similar habitat at a nearby locality in Elak Oku village.

*Etymology*.*—*In consultation with the Fon of Oku and his advisors, we have chosen a word derived from the Oku language as the species epithet. Roughly translated, the word *eysoole* (pronounced “ee-su-lay”) means “it will jump so hold it tightly,” and should be treated as a noun in apposition.

*Remarks*.*—*Based on X-ray images, the holotype (MCZ A-138016) seem to have eaten or scavenged a similarly sized *Xenopus* specimen (likely the same species) before it was collected (S15 Fig), suggesting cannibalism in this species.

#### *Xenopus (Xenopus) fraseri* Boulenger 1905

Fraser’s Clawed Frog

*Dactylethra laevis* (part) Günther ([78]; p. 2)

*Xenopus muelleri* (part) Boulenger [79]

*Syntypes*.*—*BMNH 1947.2.24.78–79, (previously BMNH 52.2.22.23–24), probably subadults, possibly males, “probably from Nigeria or Fernando Po”, coll. “Mr. Fraser”. We designate the larger specimen (BMNH 1947.2.24.78) as the lectotype.

*Referred Specimen*.*—*Ghana: Northern Region, Wa: CAS 146198 (N 10.0585, W 2.5097°; 6 August 1975; coll. P. Williams.

*Diagnosis*.*—*Because of both the morphological distinctiveness of the types and uncertainty in the type locality of *X*. *fraseri*, we restrict usage of *X*. *fraseri* to the type specimens and possibly one referred specimen (see below). *Xenopus fraseri* is distinctive among other living species of *Xenopus* in the combination of vomerine teeth and a prehallux claw. The presence of vomerine teeth differentiates *X*. *fraseri* from all living species of *Xenopus* except *X*. *muelleri* and the new tetraploid relative of *X*. *muelleri* described below [48], whereas the presence of the prehallux claw differentiates *X*. *fraseri* from *X*. *muelleri* and from some species in the *amieti* species group, including *X*. *itombwensis*, *X*. *lenduensis*, *X*. *vestitus*, and *X*. *wittei*. Because the name is restricted to the type specimens there is no information on the karyotype, advertisement call, or molecular variation.

*Description of lectotype* (BMNH 1947.2.24.78).*—*Medium-sized (SVL 37 mm), somewhat robust, sex unknown (Table 1, Figs 12 and S9–S14); rostral tip rounded in dorsal view; eyes within margins of orbit in dorsal and lateral views; prominent subocular tentacle, length more than half eye diameter and extending nearly to upper lip; eye diameter 43% of interorbital distance, approximately equal to eye–narial distance, and approximately 1.2 times distance from naris to rostral tip; internarial distance 39% of interorbital distance; vomerine teeth present (Fig 12).

Skin smooth and lacking asperities; lateral-line plaques prominent on dorsal and lateral surfaces and easily visible on the ventral surface; both horizontally and vertically oriented lateral-line plaques are well defined on dorsum and lateral surfaces of body; specimen missing all keratinous claws on left foot and only maintaining claw on third pedal digit on right foot.

Relative lengths of fingers: III > IV ≈ II > I; relative lengths of fingers when adpressed: III > IV ≈ II > I; thigh length 90% of crus length; relative length of toes: III > IV > V > II > I; no asperities on plantar surface.

*Measurements*.*—*The other syntype (now the paralectotype), BMNH 1947.2.24.79 (sex unknown) is somewhat smaller at 33 mm SVL.

*Coloration of lectotype (in alcohol)*.*—*Dorsum pale medium brown (color significantly faded) without prominent markings (S9–S14 Figs). Neuromast “stitches” are unpigmented and distinct against dorsal coloration. Venter pale yellowish gray with mostly faded melanocytes.

*Variation*.*—*Variation in the lateral-line based on the lectotype and paralectotype (except when noted *n* = 3, given as mean and range): orbital– 13 (13–13, *n* = 1); oral– 14 (13–15); medial– 21 (21–21); lateral– 23 (21–25); ventral– 24 (24–24, *n* = 1). The paralectotype is similarly faded in coloration and missing all keratinous claws on both feet.

*Vocalization*.*—*Unknown.

*Karyotype*.*—*Unknown.

*Habitat and range*.*—*Boulenger [47] described this species based on specimens previously cited as *Xenopus muelleri* [79], and before that as *X*. *laevis*, which was *Dactylethra laevis* at that time [78]. These two specimens were collected in West Africa by Louis Fraser, “probably from Nigeria or Fernando Po.”

The collection locality for the original syntypes of *Xenopus fraseri* has long been uncertain [47]. As part of our work, we were able to more certainly establish the region from which these specimens probably were collected. The two specimens later described as *X*. *fraseri* were added to the catalog of the British Museum of Natural History (now the Natural History Museum, London) in 1852 (J. Streicher, pers. comm.), when their collector, L. Fraser was based in Ouidah, today’s southern Benin. The original registration numbers were 1852.2.22.23 and 1852.2.22.24 for 1947.2.23.78 and 1947.2.23.79 respectively. Although these specimens do not have unique locality information associated with them, other specimens in the Fraser collection catalogued at the same time are from four localities: “Fernando Po” (now Bioko Island, Equatorial Guinea), “Whidah” (now Ouidah, southern Benin), “Abomey” (southern Benin), and “Budagery.”

The last locality probably refers to Badagry, southwestern Nigeria, which was a former slave port located between Ouidah and Lagos that was under British control in the 1840s and 1850s [80]. Fraser was based in Ouidah between July 1851 and November 1852 and then Lagos [81], and Badagry is approximately halfway between these two towns. Other than Fernando Po, these localities are in or on the fringe of the Dahomey Gap, an area for which few collections exist for *Xenopus*. Furthermore, based on the shared presence of vomerine teeth (Fig 12) and a prehallux claw, we have identified another specimen that is probably assignable to *X*. *fraseri* (CAS 146198) from Wa, northern Ghana, which lies to the west of the Dahomey Gap but is also in Sahel habitat. For these reasons we conclude that the type locality of *X*. *fraseri* is most likely from southern Benin or southwestern Nigeria, and not from Bioko Island.

#### *Xenopus (Xenopus) kobeli*, new species

urn:lsid:zoobank.org:act:EFB69AA8-54A3-4052-B058-D64184E1FE3C

Kobel’s Clawed Frog

*Xenopus* sp. nov. VIII *sensu* Tymowska (1991)

*Holotype*.*—*MCZ A-148037 (BJE 3075), adult female, Republic of Cameroon, Centre Region, Meganme village, N 4.6116°, E 12.2254°, 637 m, 22 October 2011, coll. B. J. Evans, D. M. Portik, M. LeBreton (Figs 6 and S3–S5).

*Paratypes*.*—*Republic of Cameroon: Centre Province: MCZ A-148035, male, MCZ A-148036, MCZ A-148038-9, females, same data as holotype; MCZ A-148059, MCZ A-148063, females, Balaoungao, N 5.2043° E 10.4289°, 1516 m, 25 October 2011; MCZ A-148060–62, males, same locality data, coll. B. J. Evans, D.M. Portik, S. Menzepoh; Ouest Region: MCZ A-148065–66, males, N 5.5703°, E 10.6097°, 1127 m, 26 October 2011, coll. B.J. Evans, D.M. Portik, S. Menzepoh; Est Region: NMP6V 74714/1, subadult male, Malapa, N 2.1028°, E 15.3566°, 388 m, 31 May, 2010, coll. V. Gvoždík, O. Kopecký.

*Diagnosis*.*—*Analysis of mitochondrial data suggests that a portion of the allopolyploid genome of *X*. *kobeli* is closely related to *X*. *ruwenzoriensis* in the Albertine Rift and that the rest of its genome is most closely related to that of other species of the *amieti* species group in Cameroon. This species is thus distinguished from all others by unique nucleotide substitutions in mitochondrial and autosomal DNA and in the unique combination of ancestral genomes from which it is derived. Similar to *X*. *ruwenzoriensis*, *X*. *longipes*, and *X*. *eysoole* (described above), *X*. *kobeli* is distinguished from most closely related species by being dodecaploid. The trill-type call of *X*. *kobeli* distinguishes it from *X*. *ruwenzoriensis*, *X*. *amieti*, *X*. *lenduensis*, and *X*. *pygmaeus* Table 4,[42]. The fewer number of pulses in the call distinguishes *X*. *kobeli* from the trill-type calls of *X*. *vestitus* and its higher dominant frequency distinguishes it from the trills of *X*. *allofraseri* (described above) and *X*. *wittei* Table 4,[42]. *Xenopus kobeli* is distinguished from *X*. *eysoole* by having a trill-type instead of a burst-type call, and from the new tetraploid species in the *amieti* species group described below by fewer pulses per call. Both *X*. *longipes* and *X*. *pygmaeus* have smaller adult body size than *X*. *kobeli*; based on specimens we examined, maximum female SVL is 34 mm in *X*. *longipes* and 36 mm in *X*. *pygmaeus*, in comparison to 47 mm in *X*. *kobeli*. The presence of a prehallux claw in *X*. *kobeli* further differentiates it from *X*. *itombwensis*, *X*. *lenduensis*, *X*. *vestitus*, and *X*. *wittei*.

*Description of the holotype*.*—*Medium-sized (SVL 42 mm), moderately robust female (Table 1, Figs 6 and S3–S5); rostral tip rounded in dorsal view; eyes projecting just beyond margins of orbit in dorsal view and beyond dorsal margin of head in lateral view; subocular tentacle moderately long, length slightly more than half of eye diameter and extending half way to upper lip; eye diameter 38% of interorbital distance, 82% of eye–narial distance, and approximately equal distance from naris to rostral tip; internarial distance 40% of interorbital distance; vomerine teeth absent (Fig 11).

Skin smooth and lacking asperities; lateral-line stitches most prominent on dorsal and lateral surfaces and extending onto ventral surface; both horizontally and vertically oriented lateral-line stitches well defined on dorsum and lateral surfaces of body, but difficult to discern ventrally.

Relative lengths of fingers: II > III ≈ IV > I; relative lengths of fingers when adpressed: II > III > I > IV; thigh length approximately equal to crus length; relative length of toes: IV > III > V > II > I; few scattered asperities on plantar surface.

*Measurements*.*—*Female specimens reach a maximum SVL of 47 mm (mean: 44 mm; *n* = 6), and males reach a maximum SVL of 38 mm (mean: 35 mm; *n* = 6) (Table 2).

*Coloration of holotype (in alcohol)*.*—*Dorsum medium gray brown with a few dark brown irregularly shaped spots on the posterior dorsum (Figs 6 and S3–S5). Neuromast “stitches” are unpigmented and well defined against dorsal and lateral coloration (Figs 6 and S3–S5). Venter yellowish cream with many small and punctate orange spots and a few scattered patches of dark melanocytes on the venter and hind limbs. Many diffuse melanocytes on the plantar surface, but they are not developed into spots. Pedal webbing unpigmented. Pale gray interocular region, not well defined into an interocular bar.

*Coloration in life*.*—*Based on color photographs of MCZ A-148062–63 (Figs 7 and S6–S8), the dorsum and limbs of *Xenopus kobeli* are brownish gray with medium gray or brown markings, and sometimes with a pale creamy gray interocular region.

*Variation*.*—*Variation in the lateral-line based on specimens at MCZ (*n* = 12 except where noted, given as mean and range): orbital– 11 (10–12, *n* = 9); oral– 16 (14–19); medial– 19 (16–22); lateral– 15 (13–21, *n* = 4); ventral– 15 (13–21, *n* = 4).

*Vocalization*.*—Xenopus kobeli* has a trill-type call (Table 4; Fig 8).

*Karyotype*.*—Xenopus kobeli* is dodecaploid with a karyotype of 2*n* = 12*x* = 108 Fig 9, [51].

*Habitat and range*.*—*Some populations of *X*. *kobeli* are known from standing water on the southern Bamiléké Plateau at sites between 1100 and 1500 m (Fig 10), whereas others are known from similar habitats in central or southeastern Cameroon at lower elevations (~380 m).

*Etymology*.*—*This species epithet is a patronym in honor of Hans Rudolf Kobel for his contributions to our understanding of the genetics and diversity of *Xenopus* [82].

#### *Xenopus (Xenopus) parafraseri*, new species

urn:lsid:zoobank.org:act:7DD73695-4F8F-40BF-A96C-92448EA9D973

Upland Clawed Frog

*Xenopus* cf. *fraseri* 2 *sensu* Evans et al. [23]

*Xenopus fraseri*-like Tinsley et al. [1]

*Xenopus fraseri* Conlon et al. [83]

*Holotype*.*—*MCZ A-148034 (field no. BJE 3068), adult female, Republic of Cameroon, Centre Region, Mfoundi Department, Old Douala Road, N 3.7931°, E 11.4170°, 715 m, 21 October 2011, coll. B. J. Evans, M. T. Kouete, D. M. Portik (Figs 6 and S3–S5).

*Paratypes*.*—*Republic of Cameroon: Centre Region: MCZ A-148027, 148029, 148031–32, 148034, females, same locality data as holotype; MCZ A-148028, 148030, 148033, males, same locality data as holotype; NMP6V 74556/1, female, Ebogo, N 3.3913° E 11.4663°, 628 m, 18 October 2009, coll. V. Gvoždík; Est Region, coll. D. C. Blackburn, B. D. Freiermuth, G. F. M. Jongsma, M. T. Kouete, D. M. Portik, R. D. Tarvin; CAS 253332–33, Doumzok community forest, N 2.6445° E 14.0312°, 530 m, 13 June 2013; CAS 253366–70, Mebam community forest, N 2.6106° E 14.0234°, 550 m, 14 June 2013; Sud Region, coll. D. C. Blackburn, B. D. Freiermuth, G. F. M. Jongsma, M. T. Kouete, D. M. Portik, R. D. Tarvin; CAS 253589–91, Dja Faunal Reserve, Mekas, N 3.1738° E 12.5271°, 648 m, 21 June 2013; CAS 253609, Dja Faunal Reserve, near Mekas, N 3.1983° E 12.5228°, 23 June 2013. Gabonese Republic: NCSM 78872, Ogooué-Ivindo Province, N 0.0426° E 12.2983°, 17 October 2011, coll. B. L. Stuart, R. C. Bell, T. Ogombet, U. Eyagui, P. Endazokou; 78877–78, same collection data, 21 October 2011.

*Referred Specimens*.*—*Republic of Congo: Cuvette-Ouest Department: Ndjoko, Ondou Forest, N 0.2518° E 14.1612° (NMP6V 75140/1, 1 female), N 0.2686° E 14.1592° (NMP6V 75140/2-5, 4 juveniles), N 0.2677° E 14.1618° (NMP6V 75140/6–7, 2 juveniles), 420–425 m, 11–17 January 2012, coll. V. Gvoždík.

*Diagnosis*.*—Xenopus parafraseri* is a tetraploid species, with a trill-type call, that exhibits all of the morphological features of subgenus *Xenopus* described above. The two other closely related tetraploid species can be distinguished from *X*. *parafraseri* by unique nucleotide substitutions in mitochondrial and autosomal DNA (Figs 1 and 2 and S1 and S2), because *X*. *pygmaeus* is smaller and has a burst-type call, and *X*. *allofraseri* has a call with fewer pulses with longer interpulse intervals as well as lower dominant frequencies. Based on sequenced samples, our records of *X*. *parafraseri* are all from localities > 400 m, whereas those of *X*. *allofraseri* range from 2–1169 m (S2 Table). *Xenopus parafraseri* differs from the other species within the *amieti* species group by being larger than *X*. *longipes* and *X*. *pygmaeus* (Table 4), having a trill-type call in contrast to the burst-type call type of *X*. *amieti*, *X*. *lenduensis*, *X*. *pygmaeus*, and *X*. *ruwenzoriensis*, or the biphasic call type of *X*. *itombwensis*. The presence of a prehallux claw in *X*. *parafraseri* further differentiates it from *X*. *itombwensis*, *X*. *lenduensis*, *X*. *vestitus*, and *X*. *wittei*. Both *X*. *parafraseri* and *X*. *allofraseri* have previously been referred to as *X*. *fraseri*, yet both are different because of the presence of vomerine teeth in *X*. *fraseri* (see discussion of the *X*. *fraseri* type specimens above). *Xenopus parafraseri* is further distinguished from several species of *Xenopus* in the amino acid sequences of a PGLa and a CPF–RP peptide in epinephrine stimulated skin secretions [83].

*Description of the holotype*.*—*Medium-sized (SVL 41 mm), moderately robust female (Table 1, Figs 6 and S3–S5); rostral tip rounded in dorsal view; eyes projecting just beyond margins of orbit in dorsal view and beyond dorsal margin of head in lateral view; subocular tentacle moderately long, length slightly more than half of eye diameter and extending half way to upper lip; eye diameter 43% of interorbital distance, 86% of eye–narial distance, and 1.2 times the distance from naris to rostral tip; internarial distance 45% of interorbital distance; vomerine teeth absent (Fig 12).

Skin smooth and lacking asperities; lateral-line stitches most prominent on dorsal and lateral surfaces and extending onto the ventral surface; both horizontally and vertically oriented lateral-line stitches well-defined on dorsum and lateral surfaces of body, but difficult to discern ventrally.

Relative lengths of fingers: III > II ≈ IV > I; relative lengths of fingers when adpressed: III ≈ II > IV > I; thigh length 1.1 times crus length; relative length of toes: IV > III ≈ V > II > I; few scattered asperities on plantar surface.

*Measurements*.*—*Female specimens reach a maximum SVL of 42 mm (mean: 37 mm; *n* = 14), and males reach a maximum SVL of 38 mm (mean: 34 mm; *n* = 3). See S1 Table for more measurements.

*Coloration of holotype (in alcohol)*.*—*Dorsum medium grayish brown with prominent dark brown mottling behind head, with some darker mottling on limbs (Figs 6 and S3–S5). Well defined pale brown region extending from midpoint of eyes to posterior of skull, with a medium brown snout. Neuromast stitches are unpigmented and distinct against dorsal coloration. Venter pale dusky cream, with many scattered orangish brown melanocytes and dark brown spots across throat, venter, and hind limbs. Pedal webbing with scattered dark melanocytes.

*Coloration in life*.*—*Based on color photographs of NCSM 78877 and CAS 249961 (Figs 7 and S6–S8) and notes from other specimens, the dorsum and limbs of *Xenopus parafraseri* range from pale to olive-gray with a medium gray interocular bar and a creamy interocular region. A creamy occipital region bordered posteriorly by black markings is often present but uniformly colored specimens are also common.

*Variation*.*—*Variation in the lateral-line based on specimens from Gabon (except when noted *n* = 32, given as mean and range): orbital– 11 (9–13, *n* = 30); oral– 10 (8–12; *n* = 29); medial– 16 (11–19); lateral– 18 (15–21); ventral– 16 (14–19, *n* = 17).

*Vocalization*.*—Xenopus parafraseri* has a trill-type call (Table 4, Fig 8) [42].

*Karyotype*.*—Xenopus parafraseri* is tetraploid with a karyotype of 2*x* = 4*n* = 36 (Fig 9).

*Habitat and range*.*—Xenopus parafraseri* occurs in southern Cameroon, central and eastern Gabon, and northwestern Republic of Congo (Fig 10). Specimens in Cameroon were collected from pools of water in agricultural plots (CAS 253767–70) and forests, and found in syntopy with *X*. *mellotropicalis*. Specimens in the Congo were found in swampy areas in pristine primary rainforest.

*Etymology*.*—*For the species epithet, we have appended the Greek word *παρά* (para), meaning “near”, to *fraseri*. Both this taxon name, *Xenopus parafraseri*, and *X*. *allofraseri* are named to maintain the historical taxonomic relationship of these populations to the taxon *X*. *fraseri*, while recognizing that they are different and distinct evolutionary lineages.

#### *Muelleri* species group

Genetic data reveal that *X*. *muelleri*, *X*. *borealis*, and the tetraploid species described below comprise a well supported clade possessing unique nucleotide substitutions in mitochondrial and autosomal DNA differentiating it from other species in the subgenus *Xenopus* [9, 16, 20–23]. However, available data fail to provide strong statistical support for the placement of *X*. *clivii* that forms a clade with other members of the *muelleri* species group in gene trees of the tightly linked autosomal genes *RAG1* and *RAG2* [9, 16, 20–22], but not those based on mitochondrial DNA [23]. In general, these species can be diagnosed by the following combination of external morphological features: (1) large body size; (2) prominent prehallux that lacks a keratinous claw; (3) prominent and long subocular tentacle, sometimes as long as two-thirds of the eye diameter; (4) lower eyelid covering majority of eye; (5) lack of a skin ridge extending along the first pedal digit from the prehallux; (6) unfused cloacal lobes. In addition, species in the *muelleri* species group are diagnosable by being tetraploid (2*n* = 4*x* = 36). Henrici & Báez [48] noted that *X*. *muelleri* is the only living *Xenopus* species with vomerine teeth. However, specimens of the new species described below have vomerine teeth, and the type specimens of *X*. *fraseri* also have vomerine teeth, see above and Boulenger [47](Fig 12).

#### *Xenopus* (*Xenopus*) *fischbergi*, new species

urn:lsid:zoobank.org:act:35E35F7A-6D88-40B4-8698-C2138DE900E8

Fischberg’s Clawed Frog

*Xenopus* “*muelleri* West”[1, 15, 17]

*Xenopus* “new tetraploid” *sensu* Evans et al. (2004)

*Xenopus alboventralis* [nomen nudum] *sensu* Salamone [67], Salamone & Montoya-Burgos [68]

*Holotype*.*—*CAS 255060 (BJE 3806), female, from breeding colony established by specimens from Federal Republic of Nigeria, Plateau State, Jos, date of collection unknown, coll. D. Rungger, B. Colombelli, C. H. Thiébaud (Figs 6 and S3–S5).

*Paratypes*.—Nigeria: CAS 255059, 255061, females, same information as holotype MHNG 2644.060 (AMNH17296), male, same information as holotype. Republic of Ghana: UWBM 5964, subadult, Brong-Ahafo Region, Bui National Park, Lobia Stream, N 8.2908° W 2.2851°, 120 m, 14 May 2011, coll. A. D. Leaché, D. M. Portik; BMNH 1983.1501–1520, juveniles, water hole, Kologu village (1^st^ settlement north of White Volta river, Bolgatanga–Tamale Road), N 10.60° E 0.85° (estimated), ~170 m, 17 March 1979, coll. R. C. Tinsley.

*Referred Specimens*.*—*Republic of Cameroon: Extreme Nord Region: NMP6V 74744/1-2, juveniles, Djingliya, Mandara Mountains, N 10.85° E 13.85°, 550 m, October 2010, coll. R. Tropek and Z. Musilová.

*Diagnosis*.—*Xenopus fischbergi* is a tetraploid species with a click-type call. Similar to some other species of *Xenopus*, the clearest evidence for separate species status is based on molecular data (e.g. Figs 1 and 2 and S1 and S2). *Xenopus fischbergi* is also distinguished from *X*. *muelleri* in peptides present in norepinephrine-stimulated skin peptides [84]. This species is distinguished from the other species in the *muelleri* species group by smaller adult body size (based on specimens examined, maximum female SVL: *X*. *fischbergi*, 63 mm; *X*. *borealis*, 75 mm; *X*. *clivii* 78 mm, *X*. *muelleri*, 81 mm; Table 2). *Xenopus fischbergi* also differs from *X*. *borealis* by having a longer subocular tentacle and a less prominent prehallux. *Xenopus fischbergi* is further differentiated from *X*. *muelleri* by having a shorter subocular tentacle and a click-type call (burst-type call in *X*. *muelleri*) [42].

*Description of the holotype*.*—*Medium-sized (52 mm), robust female (Table 1, Figs 6 and S3–S5); rostral tip rounded in dorsal view; prominent eyes projecting beyond margins of orbit in dorsal view and beyond dorsal margins of head in lateral view; prominent subocular tentacle, approximately half eye diameter and extending halfway to upper lip; eye diameter 73% of interorbital distance, 2.6 times eye–narial distance, and 1.9 times distance from naris to rostral tip; internarial distance 61% of interorbital distance; one vomerine tooth (Fig 12).

Skin smooth and lacking asperities; lateral-line stitches most prominent on dorsal and lateral surfaces and extending onto the ventral surface; both horizontally and vertically oriented lateral-line stitches on dorsum well defined.

Relative lengths of fingers: II > III > I > IV; relative lengths of fingers when adpressed: II ≈ III > I ≈ IV; thigh length 104% of crus length; relative length of toes: IV > V > III > II > I; prominent metatarsal tubercle (lacking keratinous claw); lacking asperities on plantar surface.

*Measurements*.*—*Female specimens reach a maximum SVL of 63 mm (mean: 56 mm; *n* = 6), and males reach a maximum SVL of 52 mm (mean: 51 mm; *n* = 2) (Table 2). See S1 Table for more measurements.

*Coloration of holotype (in alcohol)*.*—*The dorsum and limbs are a fairly uniform pale greenish brown with some faint darker mottling and a somewhat paler snout (Figs 6 and S3–S5). There is no interocular bar. The venter is brownish cream with faint clusters of minute dark gray melanocytes, weakly organized into spots. The pedal webbing lacks pigmentation except for a few scattered dark melanocytes.

*Coloration in life*.*—*Based on color photographs of CAS 255059–60 (Figs 7 and S6–S8), the dorsum and limbs of *Xenopus fischbergi* range from pale to olive gray without prominent darker markings, interocular bar, or head coloration.

*Variation*.*—*Variation in the lateral-line based on the three CAS type specimens (CAS 255059–60) and UWBM 5964 (*n* = 4 except where noted; given as mean and range): orbital– 14 (12–15); oral– 15 (11–16); medial– 25 (24–26); lateral– 28 (26–31), ventral– 23 (22–24, *n* = 3). Of note is that CAS 255060 has a prominent vomerine tooth (Fig 12).

*Vocalization*.*—Xenopus fischbergi* has a click-type call (Table 4, Fig 8).

*Karyotype*.*—Xenopus fischbergi* is tetraploid with a karyotype of 2*x* = 4*n* = 36 (Fig 9).

*Habitat and range*.*—*Based on specimens with available DNA sequence data, it is clear that *X*. *fischbergi* has a wide range in western Africa, extending from northeastern Chad (Guelta d’Archei; AMNH 158377) to at least western Ghana (Bui National Park; UWBM 5964) and east to northeastern Democratic Republic of the Congo and western Uganda (Fig 10).

*Etymology*.*—*This species epithet is a patronym in honor of Michael Fischberg for his contributions to our understanding of development and speciation in *Xenopus* [82].

*Remarks*.*—*Two parasite species, *Protopolystoma occidentalis and P*. *orientalis*, are strictly host specific to *X*. *fischbergi* and *X*. *muelleri* respectively [75, 76]. That the range of *X*. *fischbergi* extends to Uganda is confirmed by the detection of *P*. *occidentalis* in a specimen from this region (J. A. Jackson & RCT, unpublished, Fig 10).

## Discussion

Using information from multiple data types, including molecular variation in mitochondrial and autosomal loci, external and internal morphology, vocalization, and karyotypes, we have identified and described six new species of African clawed frog (*X*. *allofraseri*, *X*. *eysoole*, *X*. *fischbergi*, *X*. *kobeli*, *X*. *mellotropicalis*, and *X*. *parafraseri*), resurrected one (*X*. *calcaratus*), refined the type locality and distribution of another (*X*. *fraseri*), and refined the species groups within subgenus *Xenopus* (three groups: *amieti*, *laevis*, and *muelleri* species groups). Thus, species diversity of *Xenopus* is one-third higher than previously documented (now 29 instead of 22 species). Analysis of the type specimens of *X*. *fraseri* identified a unique combination of morphological characteristics (vomerine teeth and a claw on the prehallux) that clearly distinguishes this species from all other species in the subgenus *Xenopus*, but the relationship of *X*. *fraseri* to the other species will remain unclear until new specimens with genetic data become available. All species examined thus far (including all described species except *X*. *longipes* and *X*. *fraseri*) are further distinguishable from one another by characteristics of the male advertisement call; some species such as *X*. *eysoole* also exhibit intraspecific variation in advertisement call (Table 4).

Molecular variation played a crucial role in identifying these new species. In addition to other parts of the mitochondrial (and autosomal) genome(s), we have obtained sequence data from two commonly sequenced mitochondrial genes, including a portion of the *16S* and *COI* gene of all extant species of *Xenopus* except *X*. *fraseri*. These data are publicly accessible (see GenBank accession numbers) and provide a relatively simple way to unambiguously identify almost all species (although see exceptions below), and to identify putatively new species in the future. Others have used these data extensively, for example to further characterize species distributions [85–88] and to study the origin of invasive populations of *Xenopus* [89]. Photos and recordings of male vocalizations of all new species and several previously described species are also publicly accessible on AmphibiaWeb [90]; this provides another useful resource for field-based species identification.

Increased understanding of species diversity in *Xenopus* is fundamentally important to answering broader questions related to genome duplication, gene silencing, and host-parasite co-evolution. Below we first provide remarks about several issues in *Xenopus* taxonomy, and then discuss broader implications of the discovery of the new species described here.

### Taxonomic remarks

Although *Xenopus* are easily distinguished from other frog genera, discriminating species based solely on morphological characters can be sometimes difficult because of low interspecific variation. The high similarity between mitochondrial DNA sequences of various species pairs (*X*. *boumbaensis* + *X*. cf. *boumbaensis* and *X*. *eysoole* + *X*. *longipes*) highlights the point that the delineation of *Xenopus* species often requires information from nuclear DNA [20, 23]. Specimens of *X*. cf. *boumbaensis* were recently found at the Muséum d’histoire naturelle de la Ville de Genève (MHNG 2644.082–4), and additional study of live animals from the source locality of Yaoundé, Cameroon would be useful to provide information (e.g., vocalization, karyotype) for the further characterization of this putative species. Similarly, molecular divergence of mitochondrial DNA of *X*. *eysoole* and *X*. *longipes* is modest. However, the morphological differences between this species pair are striking (for example, compare the robust *X*. *eysoole* in Figs 6 and S3–S5 to the thin *X*. *longipes* in S9–S14 Figs; both are adult females). Several other vocal and internal morphological characters, and possibly their evolutionary history (see below) distinguish these species.

Another new species of *Xenopus* was recently proposed to exist based on a complete mitochondrial DNA genome from the Asashima strain of *X*. *tropicalis* [91]. The mitochondrial sequence of this strain is identical to a sequence previously reported by Evans et al. [23] from a sample originating from Liberia [91]. However, nucleotide sequences from the autosomal gene *RAG1* of this Liberia sample are closely related to other samples of *X*. *tropicalis*, a result that does not support separate species status for the Asashima strain [22]. In this study, we also identified a diverged lineage of mitochondrial DNA in samples from northern Cameroon (labeled “new species?” in Figs 1 and S1). At this time we lack information from other data types (e.g., autosomal DNA, vocalization, karyotype). Similar to the Asashima strain of *X*. *tropicalis*, additional information is required to rigorously evaluate whether this lineage is part of another described species, or a new species.

Several species of African clawed frog exhibit substantial population structure that may warrant recognition as separate species, including *X*. *gilli* [92–94], *X*. *largeni*, and *X*. *clivii* [19]. Population structure in *X*. *laevis* sensu lato [15] was recently explored using sequence data from mitochondrial and autosomal genes [2]. This study concluded that this clade comprises four previously named species (*X*. *laevis*, *X*. *victorianus*, *X*. *petersii*, and *X*. *poweri*), and further identified differentiated populations in *X*. *poweri* and *X*. *laevis*, some or all of which may comprise separate species.

### Genome duplication in *Xenopus*

African clawed frogs are unusual among terrestrial vertebrates in the number of polyploid species and the high number of octoploid and dodecaploid species [95–97]. Phylogenetic analyses presented here permit new interpretations that both support and extend previous inferences, including strong support for monophyly of the subgenera *Silurana* and *Xenopus* [9, 10, 13, 14, 16, 20–23]. In general, the topologies of these phylogenies are similar to these previous studies (e.g. [21]), although posterior probabilities of the concatenated analysis of *RAG1* and *RAG2* are higher in some cases, permitting a more detailed resolution of putative evolutionary scenarios of bifurcating and reticulating speciation events in African clawed frogs (Fig 3). Our analysis of cloned homeologs of the linked immune system-related genes *RAG1* and *RAG2* supports the contention that several polyploidization events occurred in *Xenopus*, including at least one tetraploidization event in each subgenus, at least three octoploidization events, and at least four dodecaploidization events.

Evans *et al*. [21] inferred the previous existence of three tetraploid ancestors (ancestors A, B, and C; Fig 3) that contributed their genomes to extant octoploid and dodecaploid *Xenopus*, but that are nonetheless not represented by a known extant tetraploid species. These ancestors are the ‘lost’ ancestors of *Xenopus* octoploids and dodecaploids. It was proposed, for example, that tetraploid ancestors A and B experienced allopolyploidization to give rise to the most recent common ancestor of the octoploid sister species *X*. *vestitus* and *X*. *lenduensis* (with ancestor B being the maternal ancestor based on mitochondrial DNA), even though no tetraploid descendant of either the A or B ancestor is known [21].

In the analysis of concatenated *RAG1* and *RAG2* paralogs, close phylogenetic affinities between the α and β homeologs of *X*. *pygmaeus* (a tetraploid) and the α3 and β3 homeologs of *X*. *ruwenzoriensis* (a dodecaploid), respectively, indicate that dodecaploidization of *X*. *ruwenzoriensis* was independent from that of all other dodecaploids (Figs 2 and 3 and S2). We therefore name the most recent common ancestor (MRCA) of *X*. *pygmaeus* and the α3 and β3 homeologs of *X*. *ruwenzoriensis* ancestor D. Ancestor D is not a ‘lost ancestor’ because the extant *X*. *pygmaeus* is tetraploid. Relationships among mitochondrial DNA clades suggest that ancestor D was the paternal ancestor of *X*. *ruwenzoriensis* (indicated by a dotted line in Fig 3 connecting D to the dodecaploidization event that gave rise to *X*. *ruwenzoriensis*). Following similar reasoning, close phylogenetic relationships between the α and β homeologs of *X*. *allofraseri* and *X*. *parafraseri* (both tetraploids) with the α3 and β3 homeologs of *X*. *kobeli* and the α2 and β2 homeologs of *X*. cf. *boumbaensis* (both dodecaploids) support an independent dodecaploidization of these two dodecaploid species from all other dodecaploids. We therefore name the most recent common ancestor (MRCA) of *X*. *allofraseri*, *X*. *parafraseri*, and these dodecaploid homeologs ancestor E. Ancestor E is also not a ‘lost ancestor’ because the extant *X*. *allofraseri* and *X*. *parafraseri* are tetraploid. Mitochondrial DNA relationships (Figs 1 and S1) suggest that ancestor E was the paternal ancestor of *X*. *kobeli* and *X*. cf. *boumbaensis*. Interestingly, mitochondrial DNA of *X*. *kobeli* is more closely related to that of *X*. *ruwenzoriensis* than to *X*. cf. *boumbaensis*, indicating (when combined with information above) that dodecaploidization of each of these species occurred independently. We attribute the close evolutionary relationship between portions of the genome of *X*. *kobeli*, *X*. *ruwenzoriensis*, and several other octoploid species as evidence for recent ancestry with a tetraploid ancestor (ancestor F). Before ancestor F diversified into ancestors D and E, it either was involved with at least two octoploidization events (as the maternal ancestor based on mitochondrial DNA), or it gave rise to other ‘lost ancestor’ tetraploid species that participated in these octoploidization events.

Not surprisingly, the calibration point used for the age of the diversification of extant Xenopodinae had a substantial effect on the estimated divergence times within this clade. Age estimates recovered using the calibration point of [34] (Figs 1 and 2) are about half as old as those recovered using the older calibration point of [9] (S1 and S2 Figs). The calibration of [34] is based on fossil calibrations, whereas the calibration of [9] assumes that continental drift triggered divergence of *Pipa* from other pipids ~100 mya. Using five fossil calibration points as minimum node ages, Zhang *et al*. [98] recovered an even older estimate for the age of Xenopodinae than Bewick *et al*. [9], whereas Roelants *et al*. [99] recovered an estimate of the age of Xenopodinae that was between that of [34] and [9] using multiple calibration points including a more recent (86 mya) age for divergence of *Pipa* being triggered by continental drift. While it seems plausible that continental drift played a role in pipid diversification, whether it specifically triggered divergence of *Pipa* from the African pipids is not clear. That an ancestor of platyrrhine primates and an ancestor of caviomorph rodents managed to disperse to South America from Africa across the southern Atlantic Ocean after it formed, possibly via island hopping [100], opens the possibility that pipids also dispersed across this marine barrier [34]. Overall, the sensitivity of inferences to variation in datasets, calibration regimes, and methods suggests that we still have much to learn about the timing and triggers of pipid diversification.

Our summary phylogeny, which attempts to conservatively interpret nodes with weak support or phylogenetic discordance among genes (Fig 3), depicts a sister relationship between *X*. *eysoole* and *X*. *longipes*. However, a strict interpretation of phylogenetic relationships in autosomal DNA suggests an independent origin of *X*. *eysoole* and *X*. *longipes* because the α2 homeologs of *X*. *eysoole* and *X*. *amieti* are more closely related to each other than either is to that of *X*. *longipes*. Caveats to a strict interpretation of this phylogeny include that we are unable at this time to distinguish ancestral polymorphism from true orthologous relationships, that recombination between homeologs within species could introduce error into phylogenetic estimation, and that phylogenetic error and uncertainty could cause departures of the estimated phylogeny from the true phylogeny. These caveats notwithstanding, the inferences of lost ancestors argue for further fieldwork aimed at characterizing new species of *Xenopus*.

### Male advertisement call

Phylogenetic relationships estimated here also offer insights into the evolution of male advertisement calls in African clawed frogs. Tobias *et al*. [42] noted that all species within subgenus *Silurana* produce calls with one dominant frequency and that, with the exception of *X*. *parafraseri*, all species in subgenus *Xenopus* produce male advertisement calls with two dominant frequencies. Vocalizations of several new species that were not previously analyzed (*X*. *calcaratus*, *X*. *eysoole*, and *X*. *kobeli*) support these conclusions though the recording of *X*. *allofraseri* was of insufficient quality to unambiguously determine what the second dominant frequency is (Table 4). The call of *X*. *calcaratus* suggests that a burst-type call was the call type of the tetraploid ancestor of extant tetraploids in *Silurana*.

### Pseudogenization of RAG1

Phylogenetically biased pseudogenization of *RAG1* β homeologs was previously reported for several species of subgenus *Xenopus* [16, 21]. New sequences from *X*. *eysoole* and *X*. *kobeli* provide additional examples of pseudogenization of *RAG1* β homeologs: *RAG1* homeolog β1 and β3 of *X*. *eysoole* and *RAG1* homeolog β1 of *X*. *kobeli* each contain a premature stop codon (GenBank accession numbers KT728013, KT728013, and KT728014 respectively). The stop codon of *RAG1* homeolog β3 of *X*. *eysoole* is in a unique position and presumably independently evolved. The stop codon of *RAG1* homeolog β1 of *X*. *eysoole* is also present in *RAG1* homeolog β1 of *X*. *longipes*, and the stop codon of *RAG1* homeolog β1 of *X*. *kobeli* is also present in *RAG1* homeolog β1 of *X*. *ruwenzoriensis*. No evidence of pseudogenization (stop codons, frameshift mutations) was observed in *RAG2* in these species.

### Host-Parasite co-evolution: Influences of allopolyploid evolution

The parasite fauna of *Xenopus* is characterized by its extraordinary richness. Within metazoan parasites, for instance, there are over 25 genera from 7 major invertebrate groups; a richer assemblage than in most other anurans [101]. This diversity reflects a dual origin of the parasites: some (such as *Protopolystoma*, *Dollfuschella*, *Oligolecithus* and *Progonimodiscus*) are typical of anurans; others (including *Gyrdicotylus*, *Cephalochlamys* and the camallanid nematodes) are typical of fish, representing transfers associated with ecological overlaps in habitat and diet. For both subsets of parasites, the representatives infecting *Xenopus* have exceptional specializations such as the brood pouch and velum of the leech *Marsupiobdella*, the attachment organ and excretory system of the monogenean *Gyrdicotylus*, and the ereynetal organ of the mite, *Xenopacarus* [reviewed in 101].

In addition to parallel evolution of host and parasite [52], patterns of parasite infection are influenced by allopolyploid evolution of *Xenopus*. For example, species of *Cephalochlamys* occur in all tetraploid species of subgenus *Xenopus* so far examined, but not in octoploids, even when the octoploids co-occur with infected *X*. *victorianus* [a tetraploid; 74]. This is consistent with the possibility that increased gene dosage or inheritance of resistance genes with complementary functions in octoploids confers parasite resistance. Parasites from the genus *Protopolystoma* provide a counter-example of increased susceptibility of higher ploidy levels. Most species in this parasite genus infect only one anuran host species. However, the tetraploid species *X*. *victorianus* and *X*. *parafraseri* and the octoploid species *X*. *wittei* each are infected by two *Protopolystoma* species (*X*. *victorianus*: *P*. *xenopodis* and *P*. *microsclera*; *X*. *parafraseri*: *P*. *fissilis* and *P*. *ramulosus*; *X*. *wittei*: *P*. *fissilis* and *P*. *simplicis*) [102, 103]. That *P*. *fissilis* occurs in *X*. *parafraseri* and in *X*. *wittei* could represent shared inheritance of an ancestral susceptibility derived from a diploid ancestor [76]. Interestingly, in *X*. *victorianus* and *X*. *wittei*, although two parasite species occur side-by-side in the same host populations, they never co-occur as adults within the same host individuals [103].

The effects of host interspecies hybridization (not involving genome duplication) on susceptibility to parasite infection have been investigated in *X*. *laevis* and *X*. *muelleri* [104]. These host species each have species-specific *Protopolystoma* parasites and laboratory-generated F1 hybrids are also largely resistant [104]. This study illustrates a selective advantage of host hybridization for enhanced immune function to helminth parasites that extends to other important pathogens such as viruses and bacteria. This advantage could have facilitated the establishment of newly emerged polyploid species alongside their parental species in the same habitats.

### Central Africa: A species diversity hotspot for African clawed frogs

Over half of *Xenopus* species occur in Central Africa, including the six new species described here, the resurrected species *X*. *calcaratus*, and nine other previously known species: *X*. *amieti*, *X*. *andrei*, *X*. *boumbaensis*, *X*. *epitropicalis*, *X*. *fraseri*, *X*. *longipes*, *X*. *poweri*, *X*. *pygmaeus*, *X*. *tropicalis*. This list includes representatives of both subgenera, and each species group as newly defined (but not the Ethiopian endemic *X*. *largeni*). Three of these species (*X*. *epitropicalis*, *X*. *poweri*, and *X*. *pygmaeus*) have distributions centered in the Congo Basin, *X*. *fischbergi* has a large range over much of the northern Congo Basin, but the rest are probably endemic to the portion of Central Africa northwest of the Congo River.

What could explain this high species diversity in Central Africa? Persistent forest habitat [105–107] could have played a role in maintaining or augmenting species diversity of African clawed frog. Indeed, the Albertine Rift region also hosts a high species diversity of African clawed frogs, including several octoploids (four species) and a dodecaploid, and this region probably harbored forest habitat for an extended period [106, 107]. Another feature of the Central African *Xenopus* diversity is a large number of species with high ploidy levels; specifically three of the seven octoploid species and three of the four described dodecaploid species occur in Central Africa. These species are a result of multiple independent allopolyploidization events that combined a few ancestral genomes in several unique ways. The diversity of octoploid and dodecaploid species raises the question of whether allopolyploidization conferred a selective advantage for species in Central Africa, such as those related to immune function discussed above.

## Supporting Information

S1 Fig Analysis of mitochondrial DNA data using the calibration from [9]. Labeling follows Fig 1.
(TIF)

S2 Fig Analysis of mitochondrial DNA data using the calibration from [9]. Labeling follows Fig 2.
(TIF)

S3 Fig Full size version of Fig 6, part one of three. (TIF)

S4 Fig Full size version of Fig 6, part two of three. (TIF)

S5 Fig Full size version of Fig 6, part three of three. (TIF)

S6 Fig Full size version of Fig 7, part one of three. (TIF)

S7 Fig Full size version of Fig 7, part two of three. (TIF)

S8 Fig Full size version of Fig 7, part three of three. (TIF)

S9 Fig Photographs of type material from several previously described *Xenopus* taxa part one of six. Images include, from subgenus *Silurana*: *X*. *tropicalis* (BMNH 1947.2.24.83) and *X*. *epitropicalis* (BMNH 1982.462), from *amieti* species group: *X*. *amieti* (MHNG 2030.80), *X*. *andrei* (MHNG 2088.32), *X*. *boumbaensis* (MHNG 2080.31), *X*. *itombwensis* (MCZ A-138192), *X*. *longipes* (MHNG 2497.10), *X*. *ruwenzoriensis* (MHNG 2238.15), and *X*. *lenduensis* (MCZ A-139853), from *laevis* species group: *X*. *laevis sudanensis* (= *X*. *poweri*; MHNG 1017.74) and *X*. *laevis bunyoniensis* (= *X*. *victorianus*; MCZ A-14616), and *X*. *fraseri* (BMNH 1947.2.24.78). Scale bar is 5 mm.
(TIF)

S10 Fig Photographs of type material from several previously described *Xenopus* taxa part two of six. (TIF)

S11 Fig Photographs of type material from several previously described *Xenopus* taxa part three of six. (TIF)

S12 Fig Photographs of type material from several previously described *Xenopus* taxa part four of six. (TIF)

S13 Fig Photographs of type material from several previously described *Xenopus* taxa part five of six. (TIF)

S14 Fig Photographs of type material from several previously described *Xenopus* taxa part six of six. (TIF)

S15 Fig X-ray of *X*. *eysoole* holotype (MCZ A-138016). (TIF)

S1 Table Specimens examined and morphological data. (XLSX)

S2 Table Specimens for which sequence data were obtained. (XLSX)

S3 Table Information on CT scan settings (excluding *X*. *calcaratus* type). (XLSX)
