# Supplementary figures and images for: Genetics, Morphology, Advertisement Calls, and Historical Records Distinguish Six New Polyploid Species of African Clawed Frog (Xenopus, Pipidae) from West and Central Africa
Source: PLoS One. 2015 Dec 16;10(12):e0142823. doi: 10.1371/journal.pone.0142823 (PMC4682732; doi:10.1371/journal.pone.0142823)

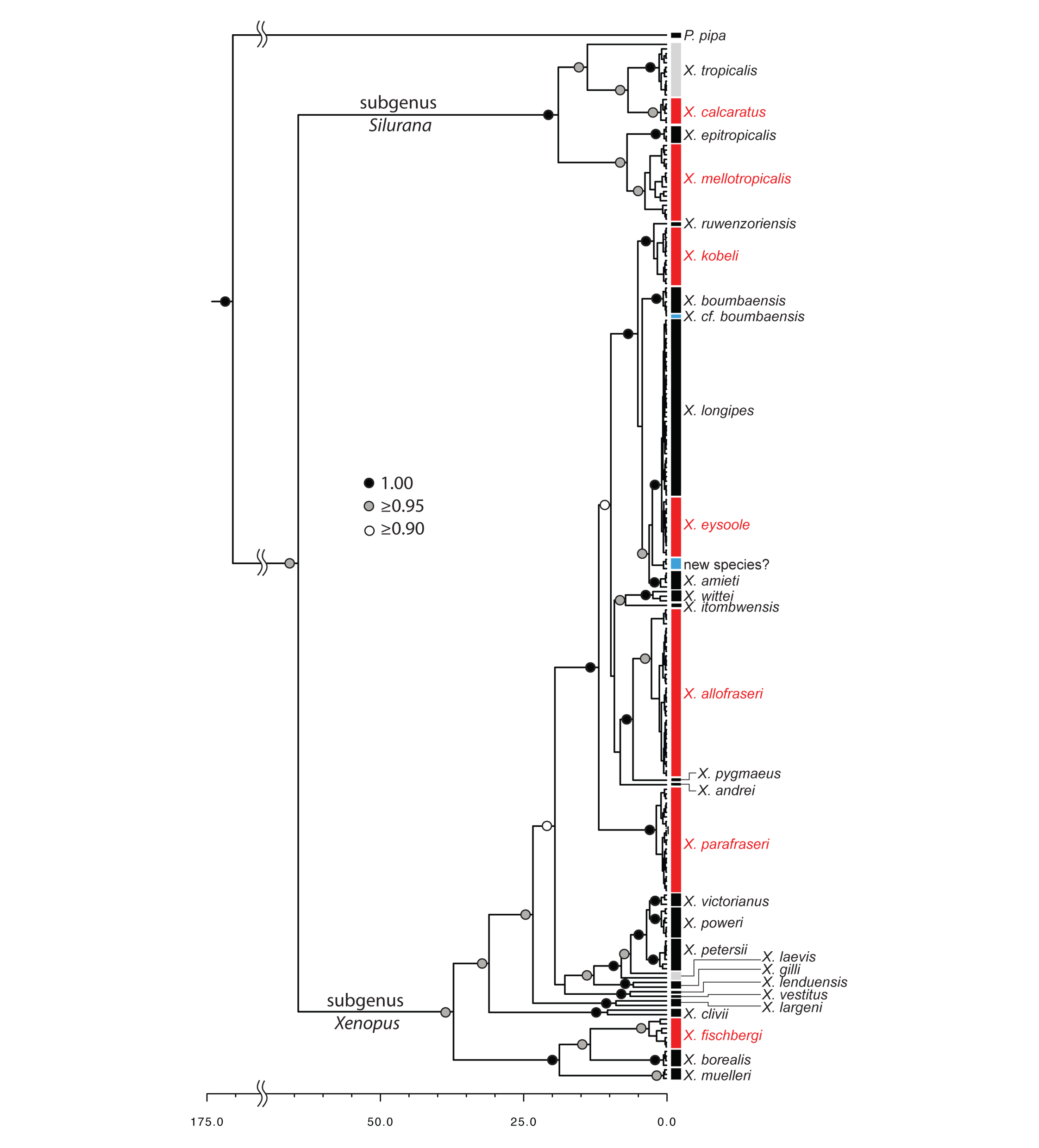

Supplement: S1 Fig — Labeling follows Fig 1. (TIF) [file pone.0142823.s001.tif]

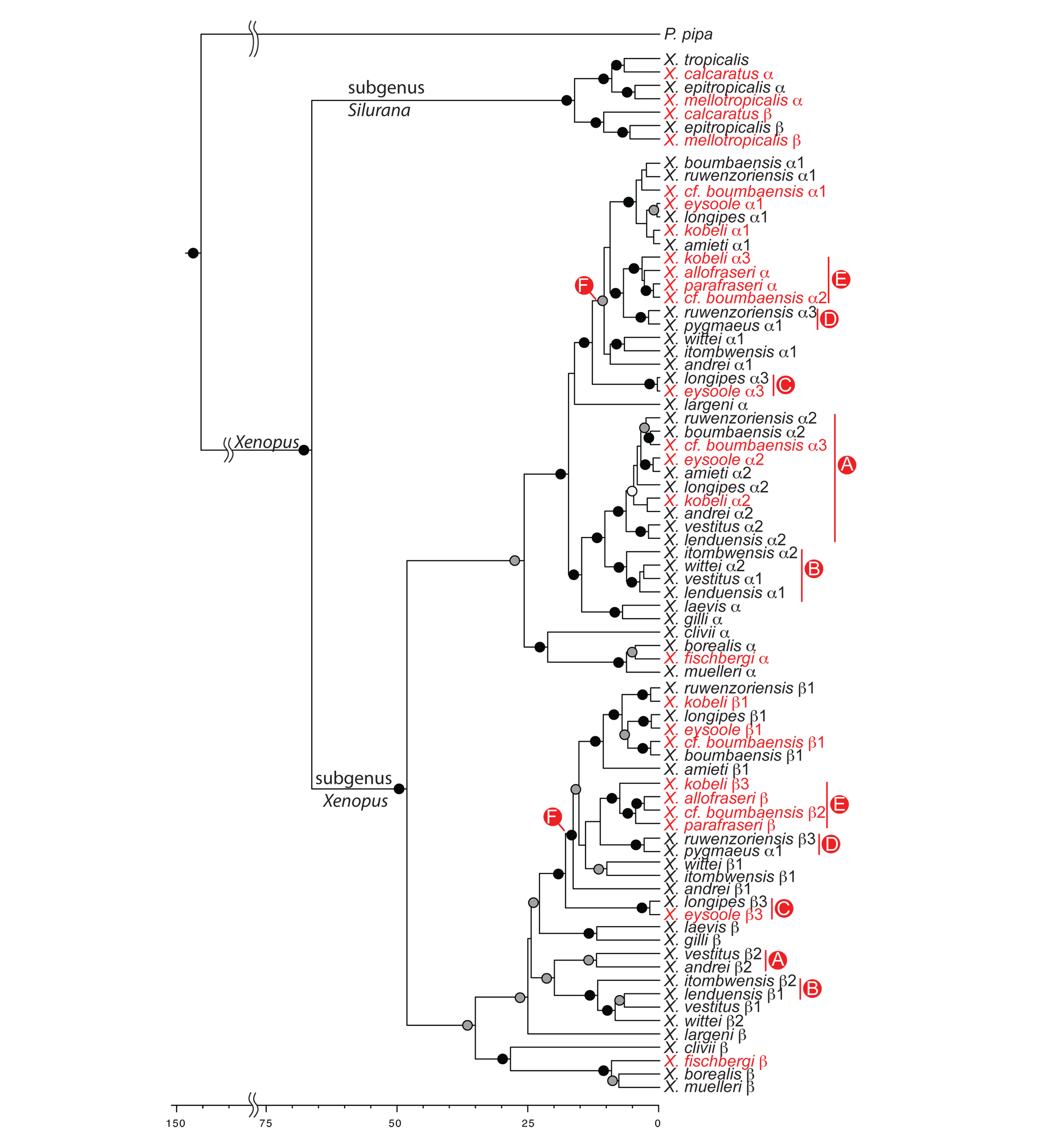

Supplement: S2 Fig — Labeling follows Fig 2. (TIF) [file pone.0142823.s002.tif]

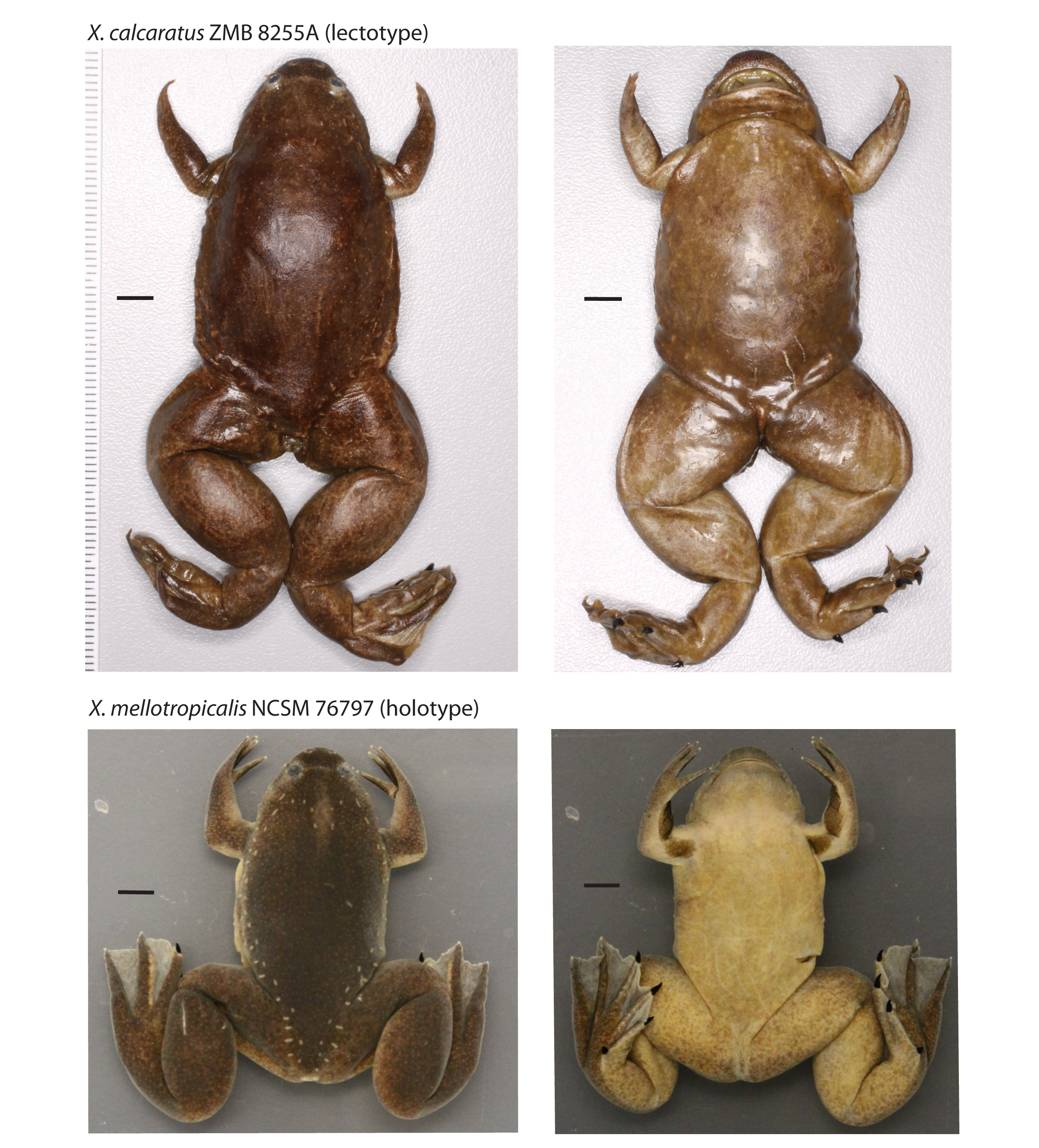

Supplement: S3 Fig — (TIF) [file pone.0142823.s003.tif]

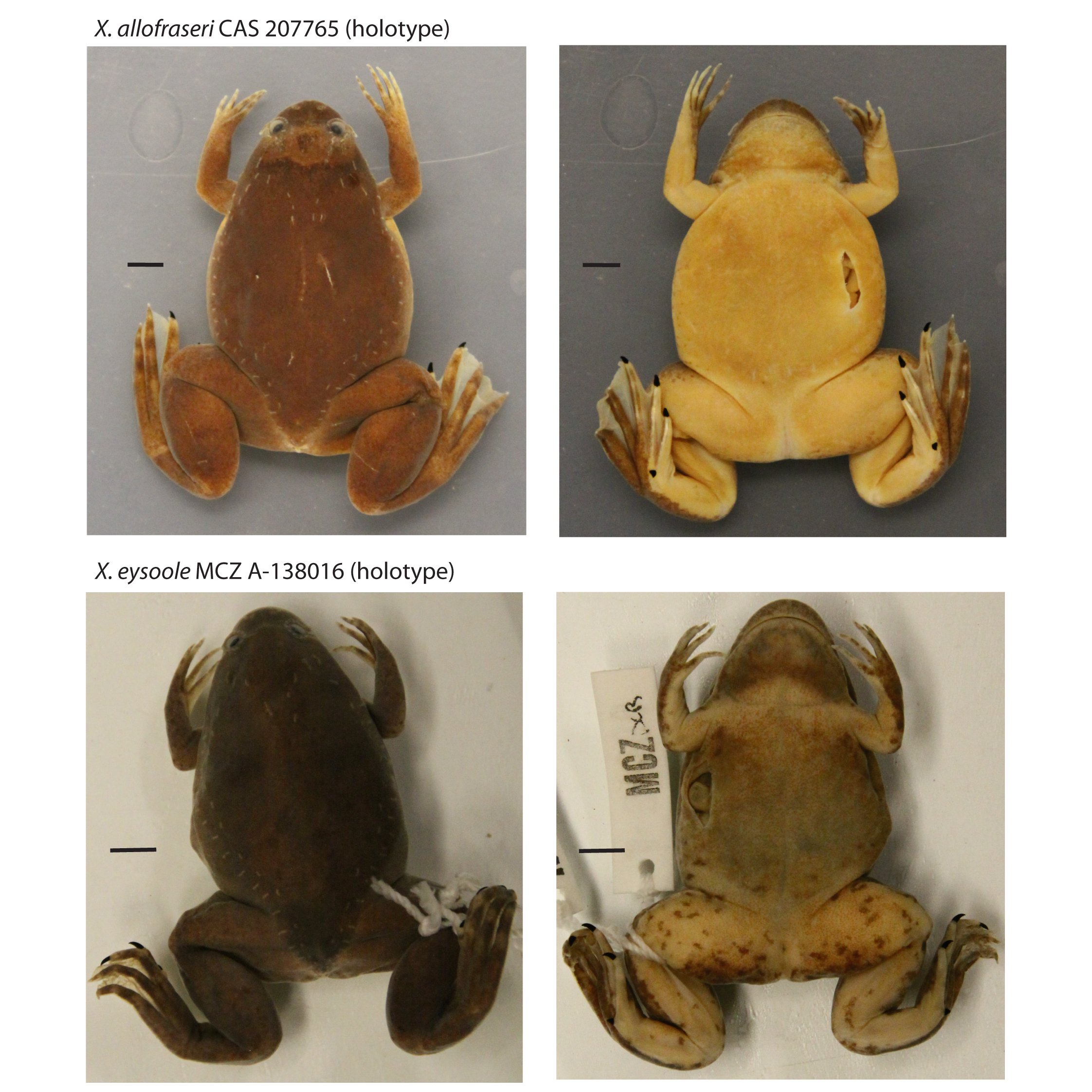

Supplement: S4 Fig — (TIF) [file pone.0142823.s004.tif]

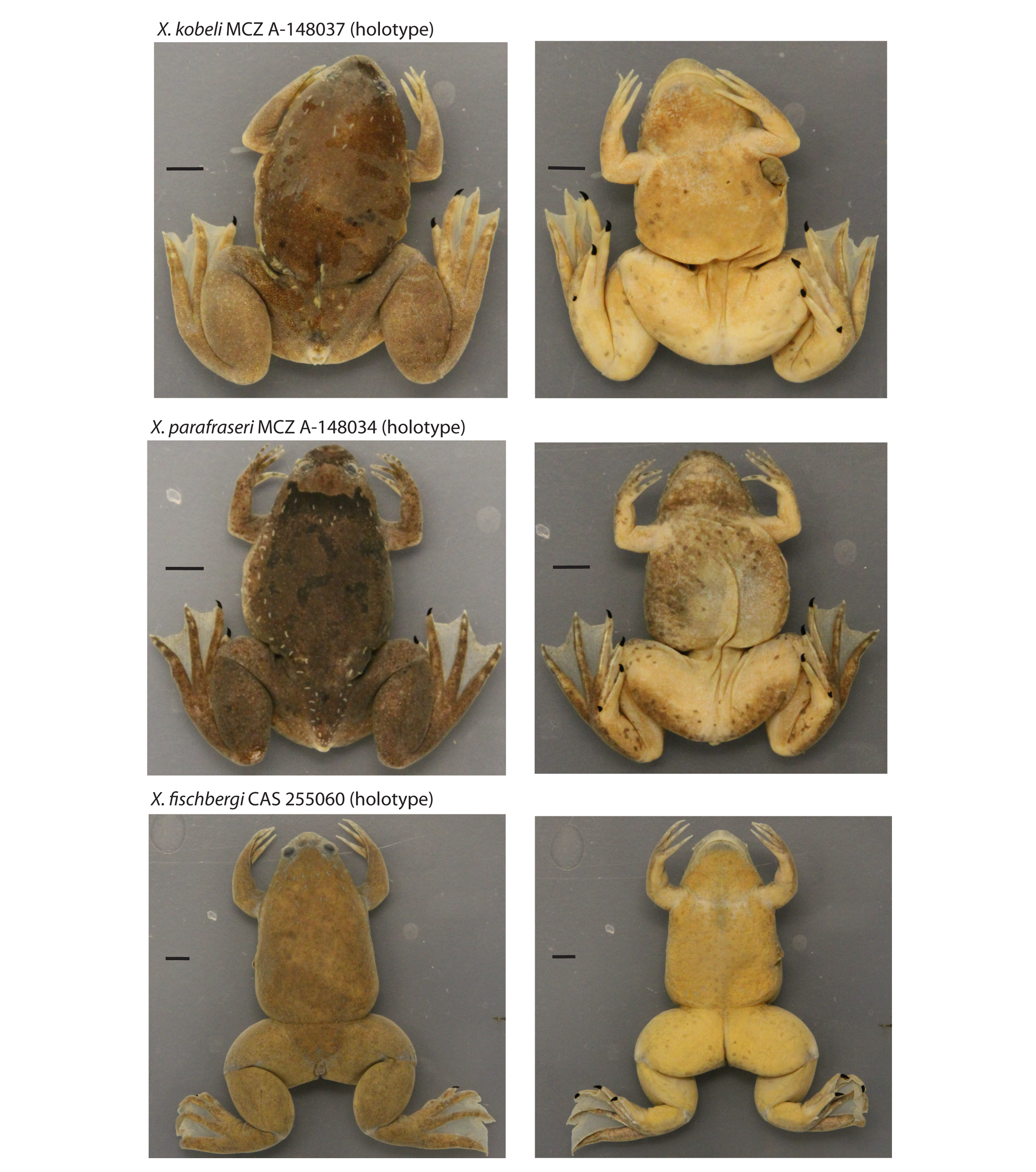

Supplement: S5 Fig — (TIF) [file pone.0142823.s005.tif]

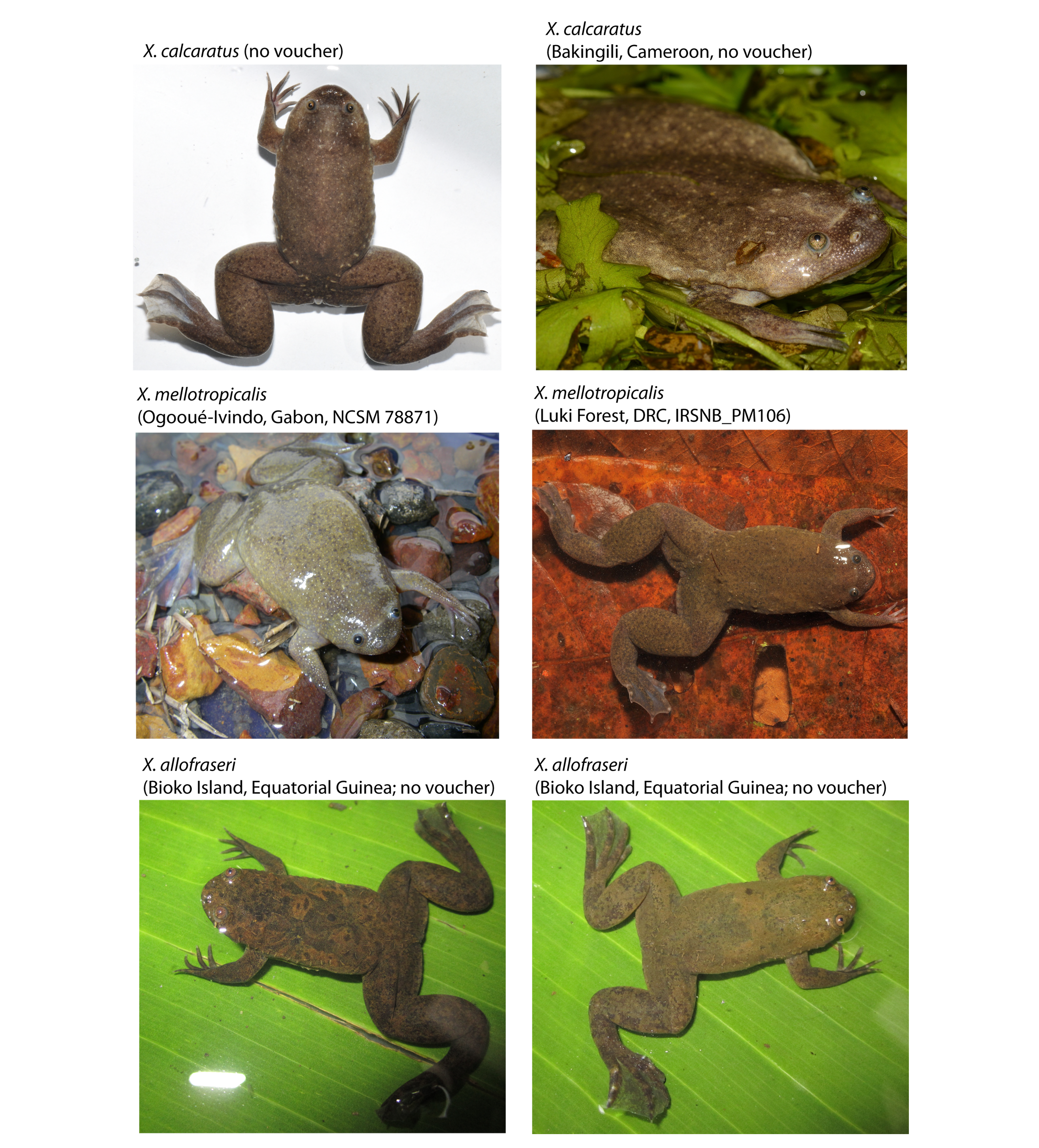

Supplement: S6 Fig — (TIF) [file pone.0142823.s006.tif]

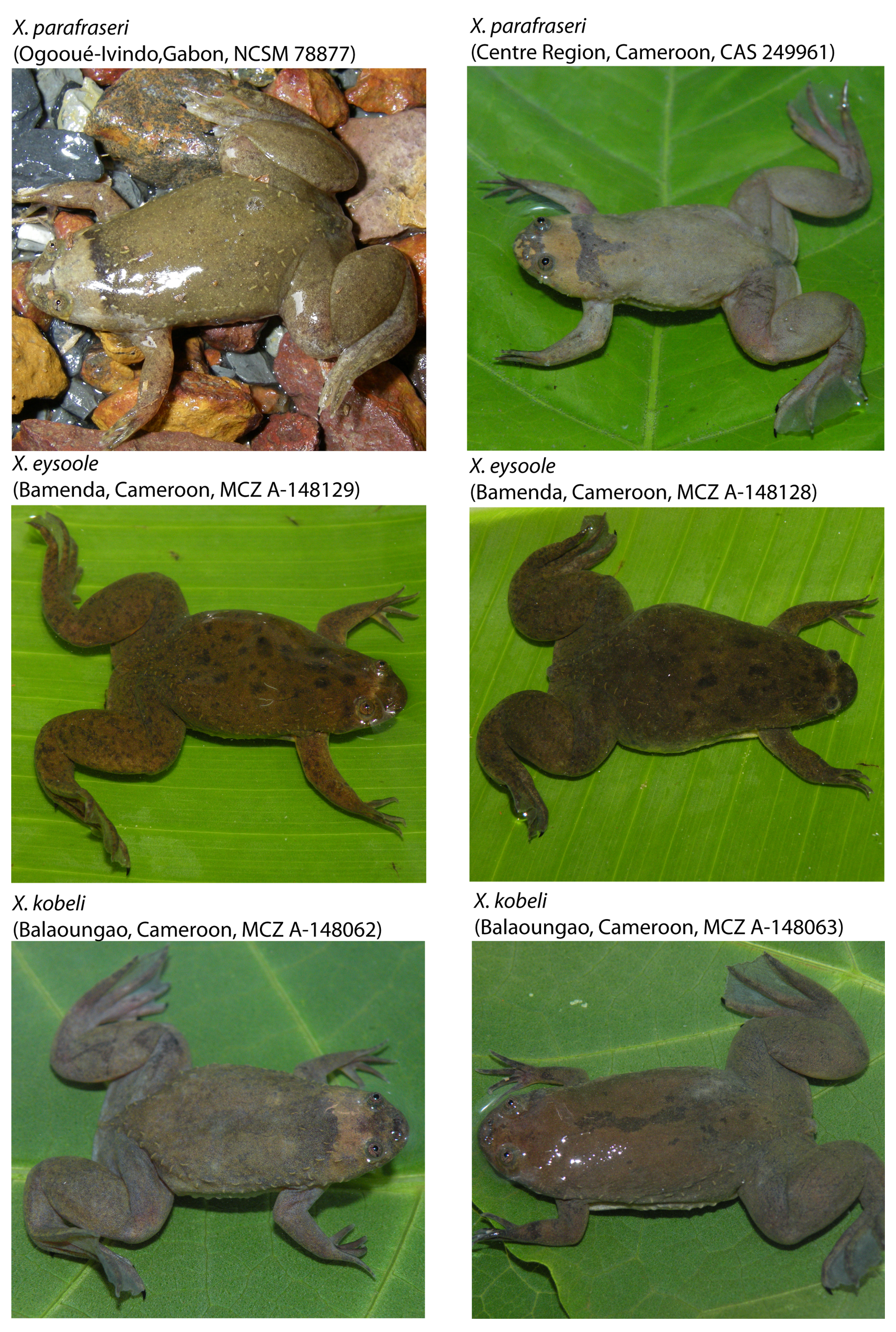

Supplement: S7 Fig — (TIF) [file pone.0142823.s007.tif]

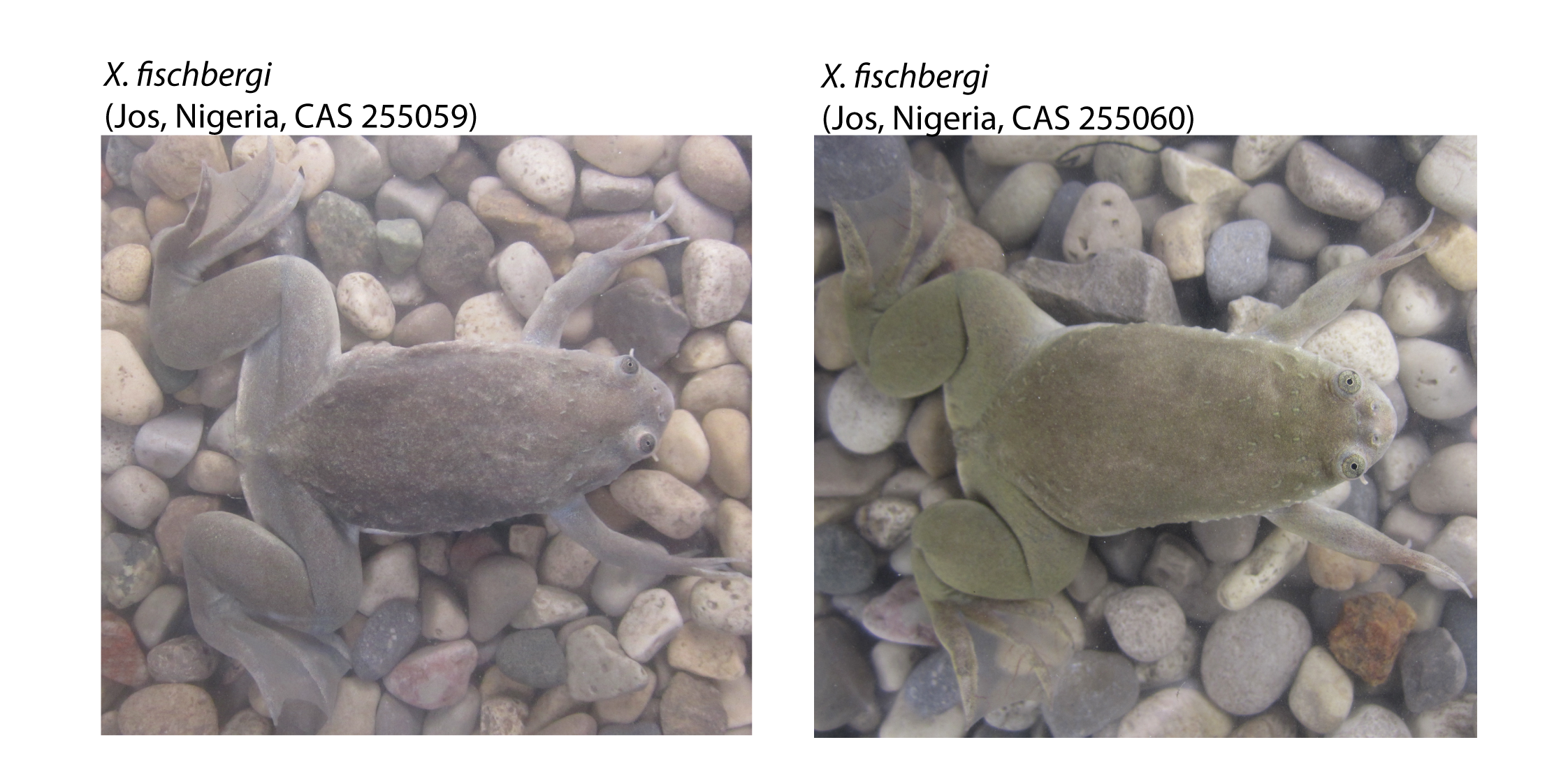

Supplement: S8 Fig — (TIF) [file pone.0142823.s008.tif]

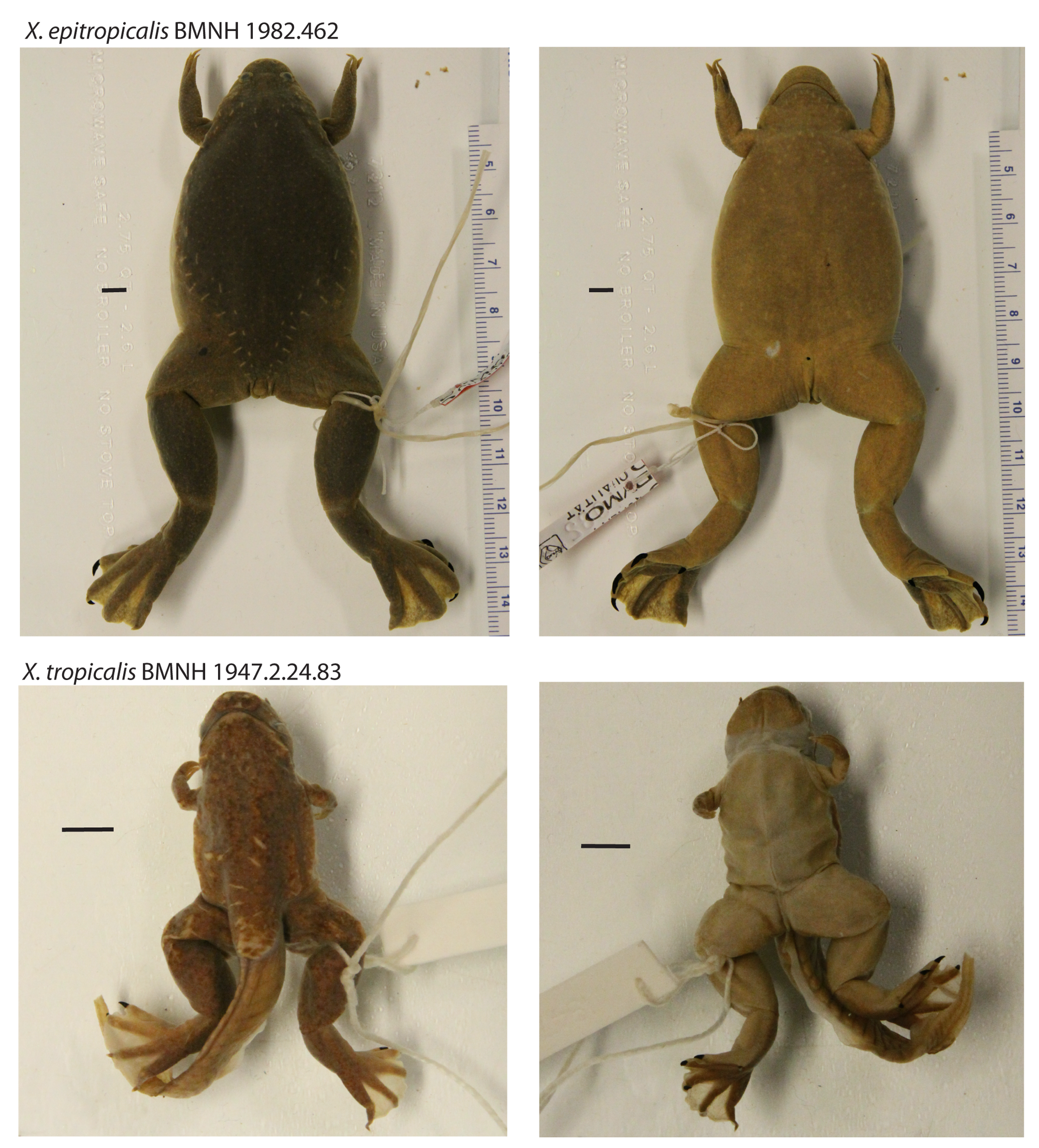

Supplement: S9 Fig — Images include, from subgenus Silurana: X. tropicalis (BMNH 1947.2.24.83) and X. epitropicalis (BMNH 1982.462), from amieti species group: X. amieti (MHNG 2030.80), X. andrei (MHNG 2088.32), X. boumbaensis (MHNG 2080.31), X. itombwensis (MCZ A-138192), X. longipes (MHNG 2497.10), X. ruwenzoriensis (MHNG 2238.15), and X. lenduensis (MCZ A-139853), from laevis species group: X. laevis sudanensis (= X. poweri; MHNG 1017.74) and X. laevis bunyoniensis (= X. victorianus; MCZ A-14616), and X. fraseri (BMNH 1947.2.24.78). Scale bar is 5 mm. (TIF) [file pone.0142823.s009.tif]

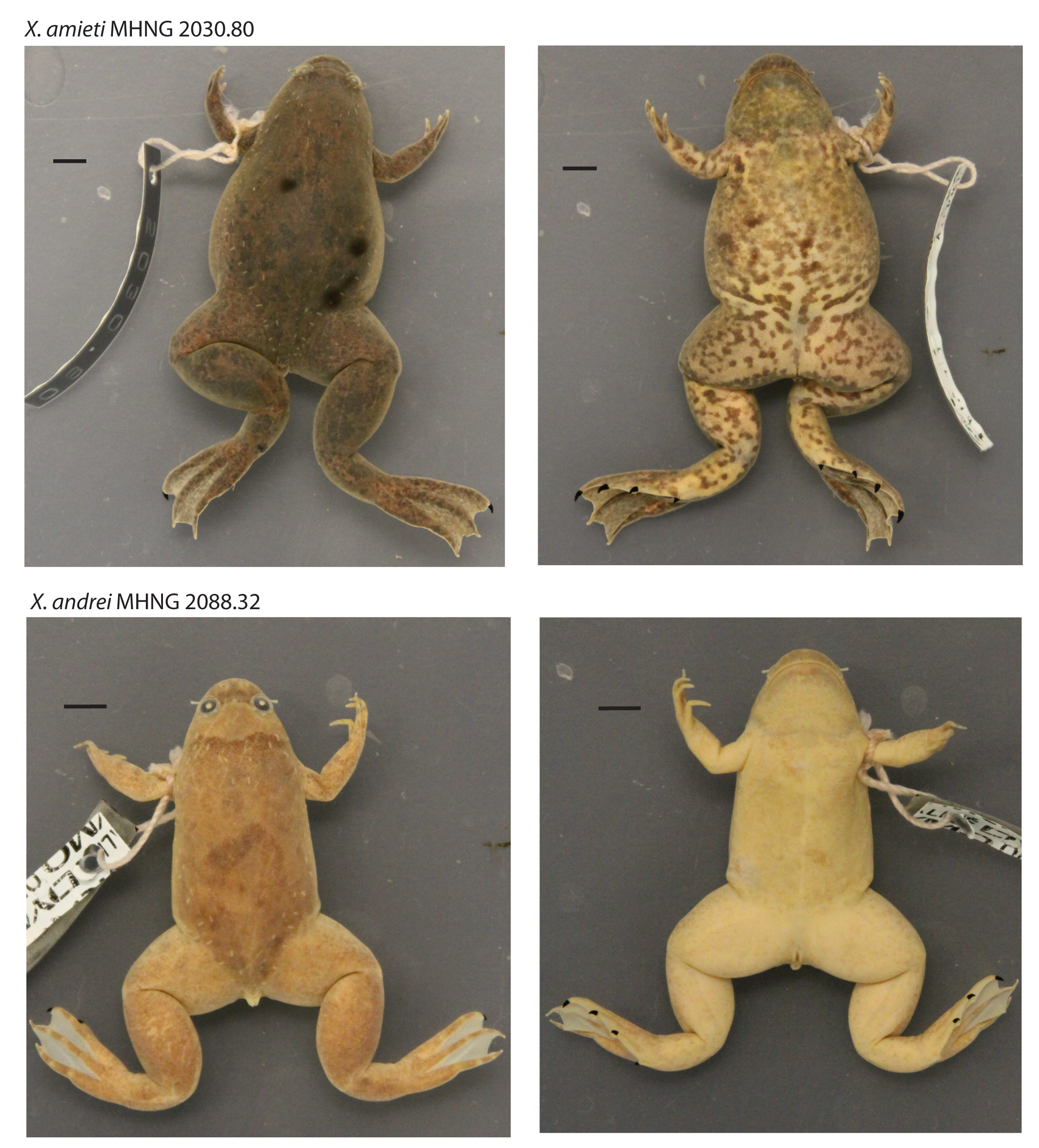

Supplement: S10 Fig — (TIF) [file pone.0142823.s010.tif]

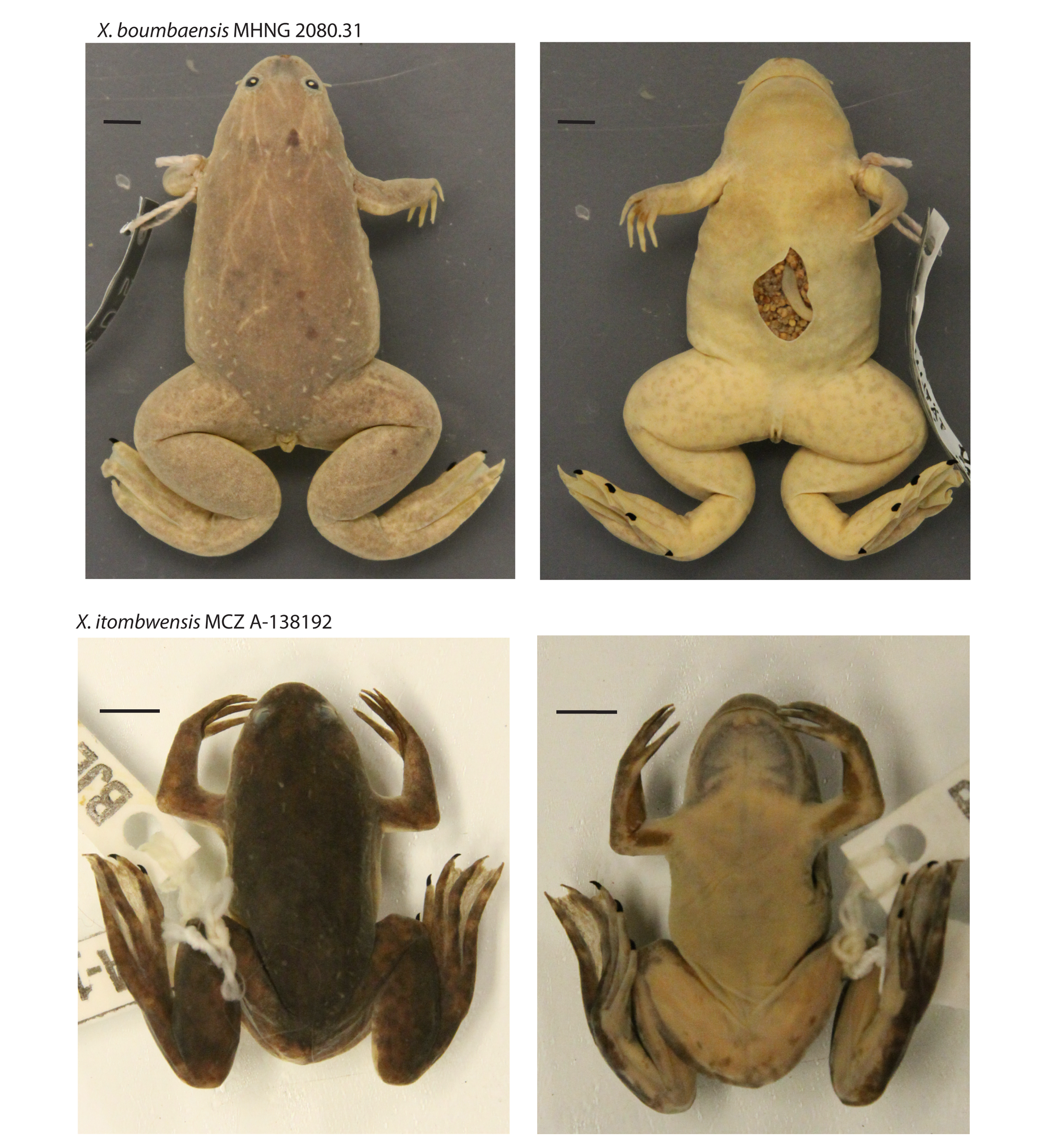

Supplement: S11 Fig — (TIF) [file pone.0142823.s011.tif]

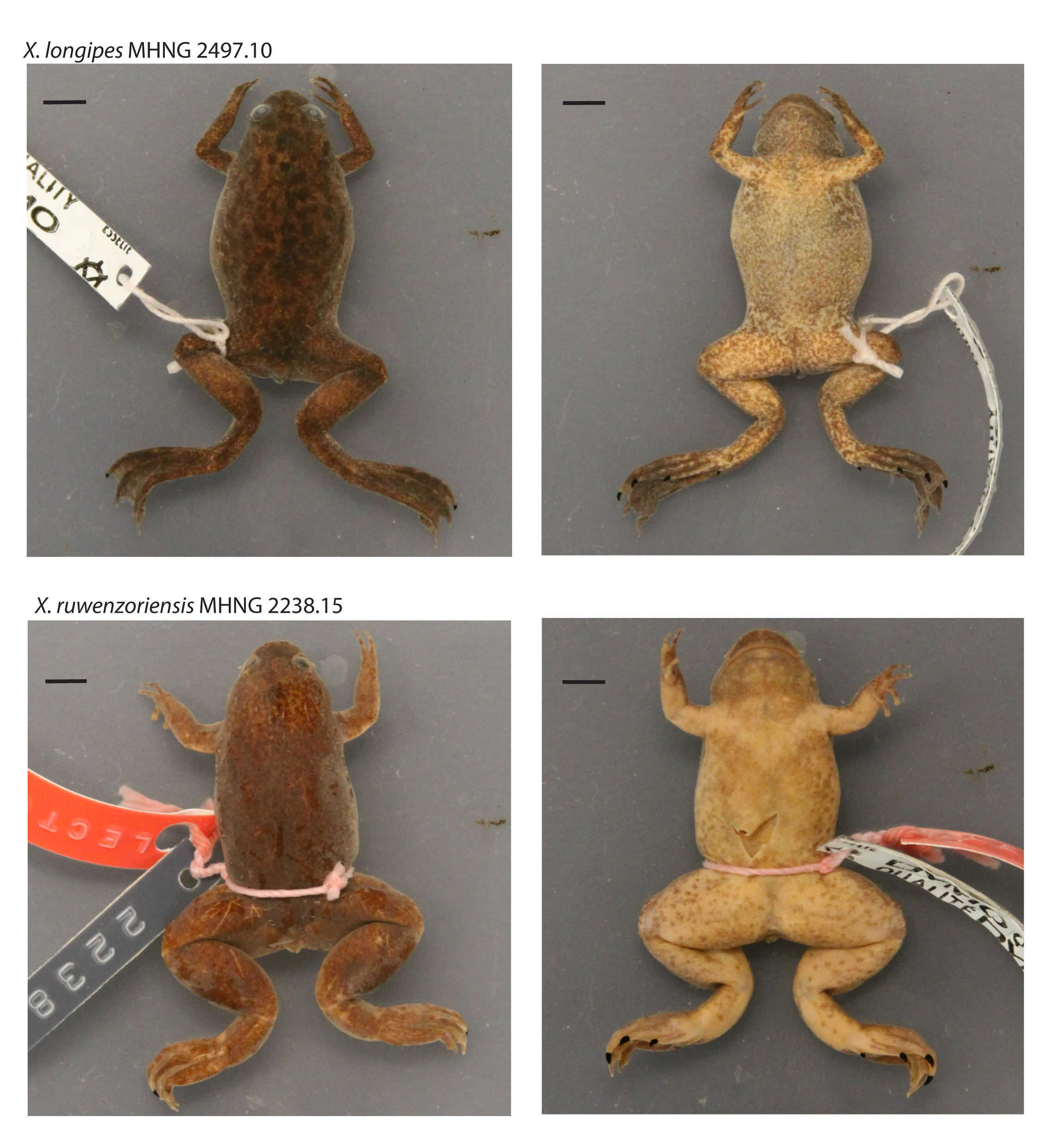

Supplement: S12 Fig — (TIF) [file pone.0142823.s012.tif]

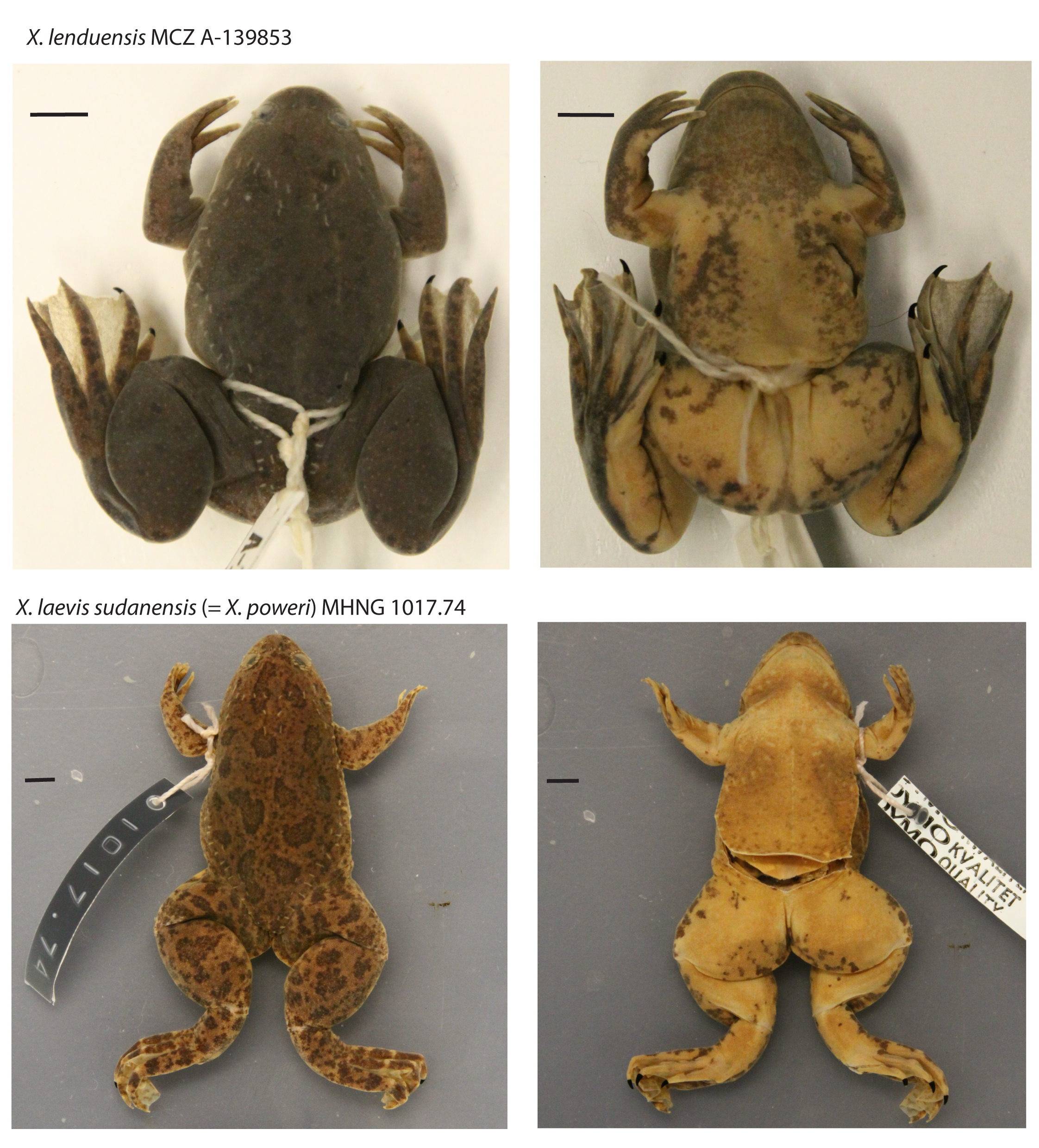

Supplement: S13 Fig — (TIF) [file pone.0142823.s013.tif]

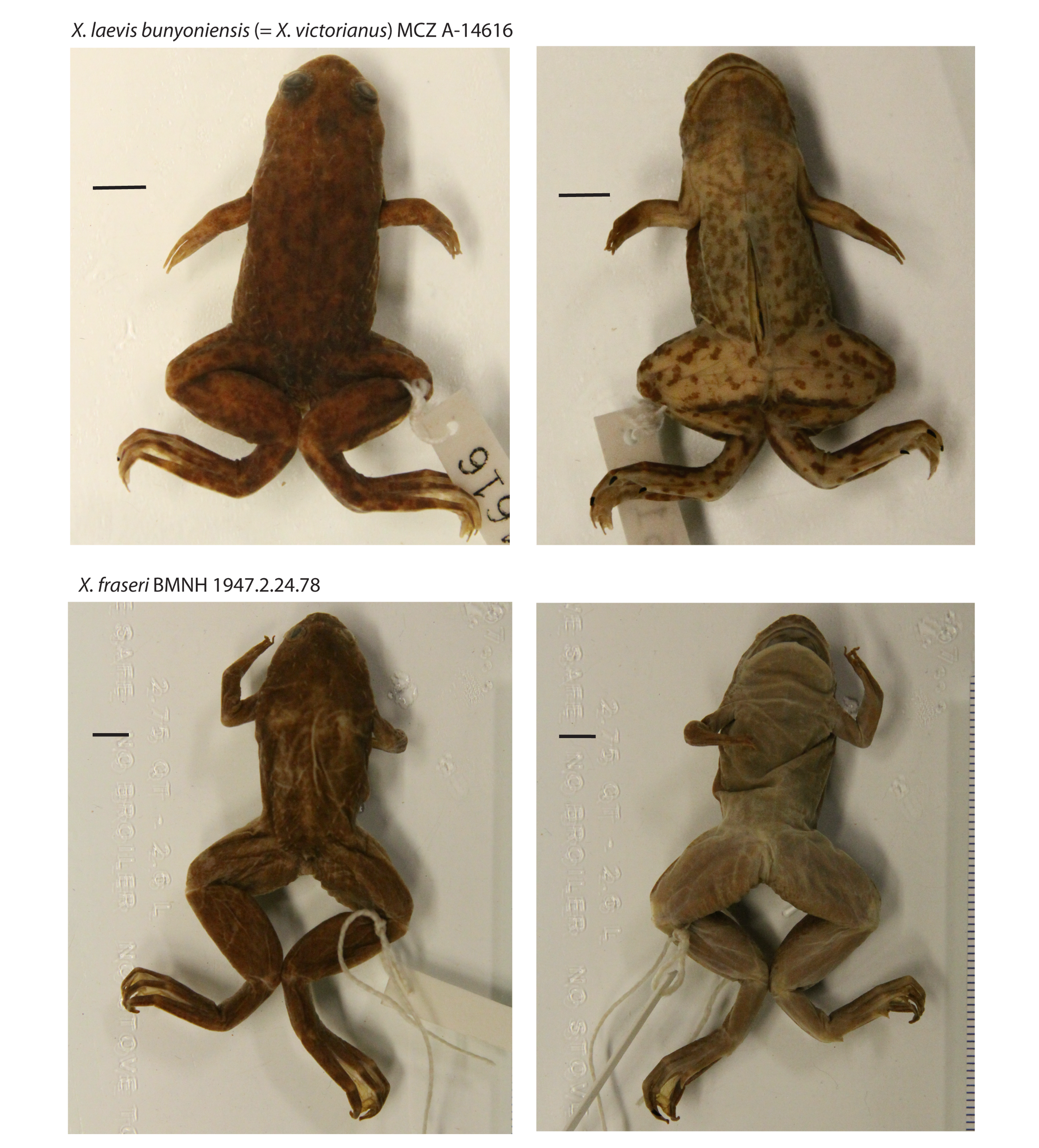

Supplement: S14 Fig — (TIF) [file pone.0142823.s014.tif]

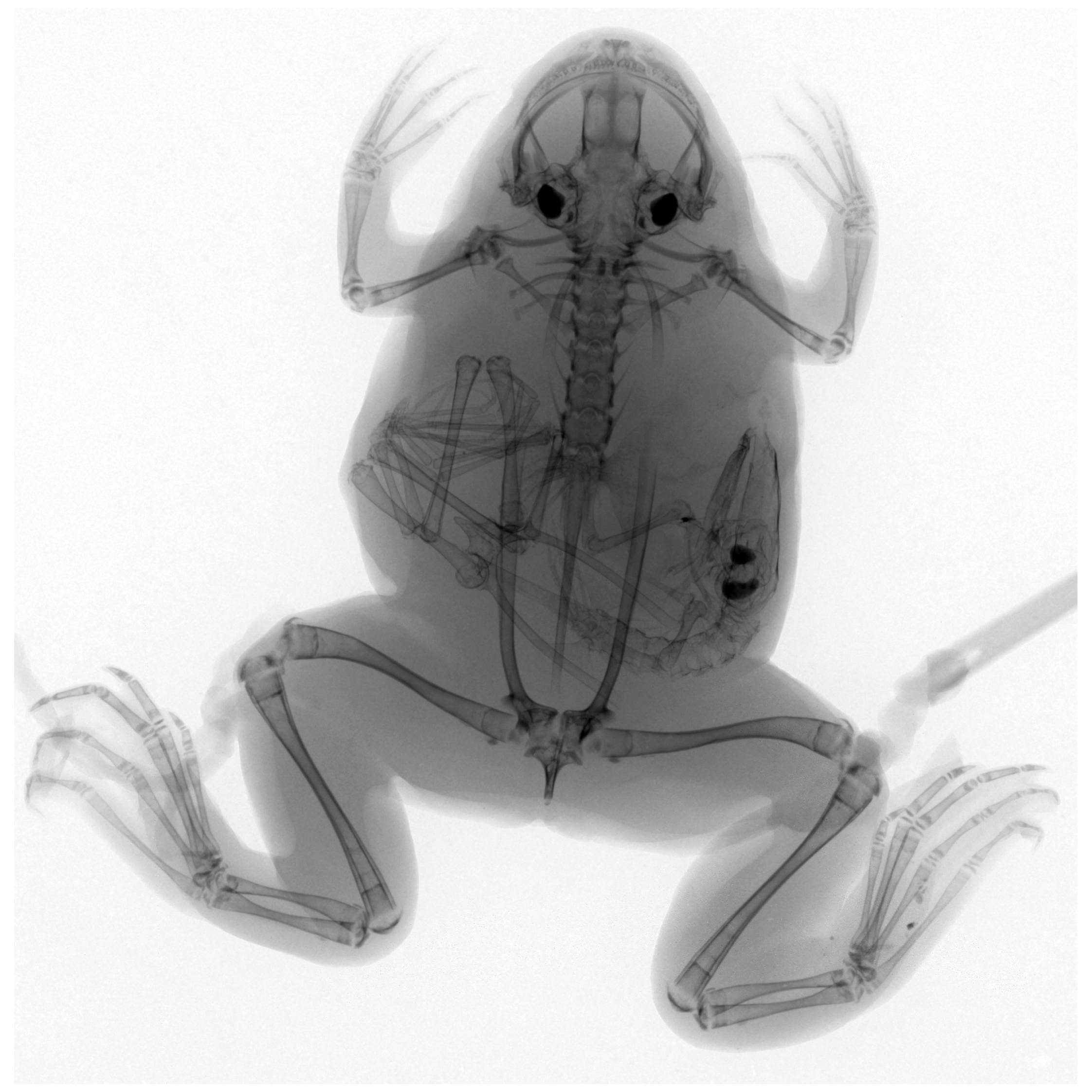

Supplement: S15 Fig — (TIF) [file pone.0142823.s015.tif]
